# Denigrins H–L: Sulfated Derivatives of Denigrins D and E from a New Zealand *Dictyodendrilla* c.f. *dendyi* Marine Sponge

**DOI:** 10.3390/md22050231

**Published:** 2024-05-20

**Authors:** Lauren Gris, Michèle R. Prinsep, Linda M. Peters, Christopher N. Battershill

**Affiliations:** 1 Chemistry and Applied Physics, School of Science, University of Waikato, Private Bag 3105, Hamilton 3240, New Zealand; lg139@students.waikato.ac.nz; 2 Biomedical, Molecular and Cellular Biology, School of Science, University of Waikato, Private Bag 3105, Hamilton 3240, New Zealand; linda.peters@waikato.ac.nz; 3 School of Science, University of Waikato Coastal Marine Field Station, 58 Cross Road, Sulphur Point, Tauranga 3110, New Zealand; christopher.battershill@waikato.ac.nz

**Keywords:** denigrin, sponge, pyrrole, pyrrolone, sodium sulfate, alkaloids

## Abstract

Five new sulfated arylpyrrole and arylpyrrolone alkaloids, denigrins H–L (**1**–**5**), along with two known compounds, dictyodendrin B and denigrin G, were isolated from an extract of a New Zealand *Dictyodendrilla* c.f. *dendyi* marine sponge. Denigrins H–L represent the first examples of sulfated denigrins**,** with denigrins H and I (**1**–**2**), as derivatives of denigrin D, containing a pyrrolone core, and denigrins J–L (**3**–**5**), as derivatives of denigrin E (**6**), containing a pyrrole core. Their structures were elucidated by interpretation of 1D and 2D NMR spectroscopic data, ESI, and HR-ESI-MS spectrometric data, as well as comparison with literature data. Compounds **1–5**, along with six known compounds previously isolated from the same extract, showed minimal cytotoxicity against the HeLa cervical cancer cell line.

## 1. Introduction

**Figure 1:**
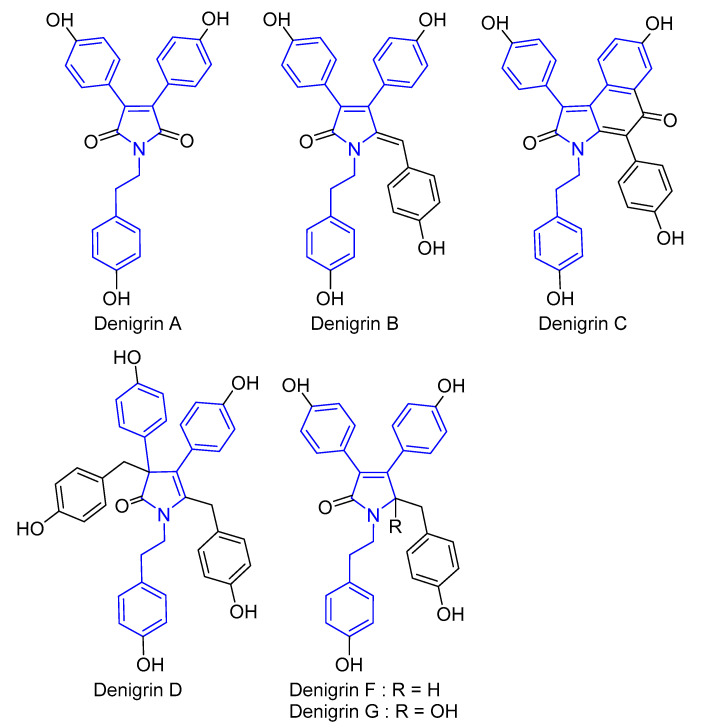
Structures assigned to the previously reported denigrins with the general structure of denigrin type compounds displayed in blue.

Marine sponges are an important source of biologically active secondary metabolites containing unique structures and displaying great diversity [1,2]. These metabolites include the denigrins, a rare class of highly substituted pyrrole and pyrrolidone alkaloids, to date only described from marine sponges. The first three denigrins A–C were isolated in 2014 by Kumar et al. with the aim of identifying new antitubercular agents from the Indian marine sponge *Dendrilla nigra* (Figure 1). Denigrin C exhibited moderate activity against *Mycobacterium tuberculosis* (IC_50_ 4 μg/mL) [3]. The total synthesis of denigrins A and B was achieved in three and five steps, respectively, from maleic anhydride by Karak et al. in 2018 [4]. In 2020, further denigrins D–G were isolated from a collection of *Dactylia* sp. nov. from the Maldives, as a means to establish potential new inhibitors of the oncogenic PAX3-FOXO1 fusion gene (Figure 1) [5]. The structure elucidation of denigrin D required the use of ^1^H−^15^N HMBC and LR-HSQMBC NMR experiments on account of the proton-deficient nature of the denigrins. In 2022, the total synthesis of denigrin E from *p*-anisaldehyde and its oxidative conversion into denigrin D, through a unique rearrangement, was reported by Chen et al. [6]. The authors confirmed the structures of both natural products and report obtaining denigrin D with 61% yield when denigrin E was reacted with *t*-BuOOH at 80 °C in the presence of Mo(CO)_6_. 

We have previously investigated the New Zealand nudibranch *Goniobranchus aureomarginatus* and its feeding preferences, utilizing two marine sponges, *Dysidea teawanui* sp. nov [7] and *Dictyodendrilla* c.f. *dendyi* [8] (the latter previously referred to as an undescribed Dictyodendrillid sponge). Feeding-choice experiments indicated a strong preference for *D.* c.f. *dendyi* [9]. The predator–prey relationship was further established by isolating six known alkaloids, dictyodendrins C, D, and F, denigrin E, dactypyrrole A, and lamellarin O1 from *D.* c.f. *dendyi*, with dictyodendrins C and F also isolated from the nudibranch extract [9]. Following this study, we further investigated the *D.* c.f. *dendyi* methanol/dichloromethane crude extract, with a focus on the minor compounds, and wish to report the isolation and structure elucidation of denigrins H–L (**1**–**5**), five new sulfated derivatives of either denigrin D or E, as well as two known compounds, dictyodendrin B and denigrin G. The ^13^C NMR assignments and spectrum of denigrin E (**6**) in CD_3_OD are also reported, due to compounds **3**–**5** being closely related to **6**, which was previously fully characterized in pyridine-*d_5_* [5], with only the ^1^H NMR spectrum reported in CD_3_OD [9].

## 2. Results and Discussions

### 2.1. Structure Elucidation

**Figure 2:**
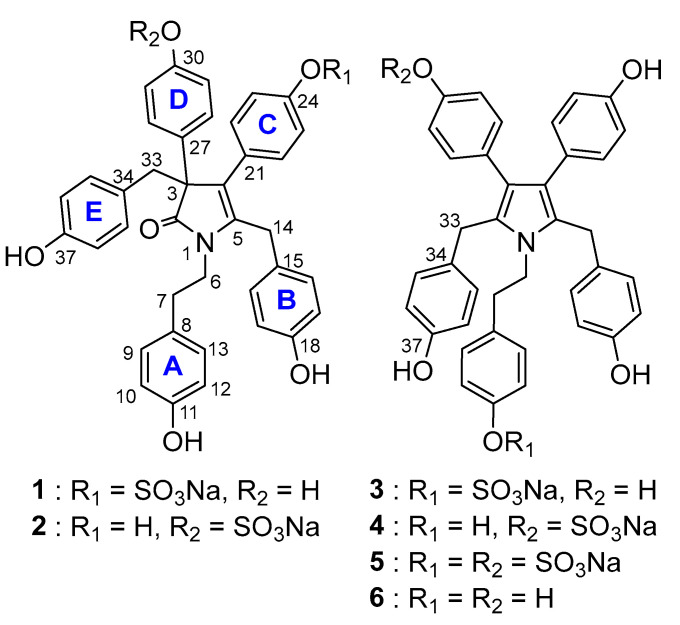
Chemical structures of compounds **1**–**6**.

Following our prior investigation of the *D.* c.f. *dendyi* sponge (order: Dendroceratida, family: Dictyodendrillidae), the methanol/dichloromethane crude extract was further investigated. Repeated reversed-phase bench column chromatography followed by repeated size exclusion chromatography and C18 reversed-phase HPLC afforded five new alkaloids, denigrins H (**1**, 1.2 mg), I (**2**, 0.8 mg), J (**3**, 2.1 mg), K (**4**, 2 mg), and L (**5**, 1.2 mg), all sulfated derivatives of either denigrin D or E (Figure 2).

Denigrin H (**1**) was isolated as a light yellow, amorphous solid exhibiting UV absorption maxima at 226 and 279 nm. The IR spectrum showed strong absorptions for the hydroxy (3369 cm^−1^) and carbonyl (1663 cm^−1^) groups. HR-ESI-MS yielded the [M − Na]^−^ ion at *m/z* 678.1803, corresponding to a molecular formula of C_38_H_32_NO_9_S. The ^1^H NMR and HSQC spectroscopic data of **1** (Table 1) displayed eight methylene signals (δ_H_ 2.19, 2.70, 3.09, 3.11, 3.28, 3.41, 3.64, 3.77) representing four methylene groups and ten doublets (δ_H_ 6.34, 6.64, 6.65, 6.705, 6.712, 6.72, 6.79, 6.87, 7.07, 7.25) accounting for a total of 20 aromatic protons, indicating five *para*-substituted benzene rings, with each doublet displaying a peak shape characteristic of magnetic inequivalence for *para*-disubstituted benzene. The ^1^H NMR spectrum of **1** was analogous to that of denigrin D recorded in CD_3_OD [5], except for two aromatic doublets shifted downfield to 6.79 and 7.07 ppm, instead of within the 6.56–6.71 ppm region, with the COSY spectrum indicating that the protons represented by these doublets were mutually coupled. By comparison with the ^1^H NMR data of dictyodendrin D [10] (a closely related alkaloid), these δ_H_ shifts are characteristic of the presence of a sulfate group in the *para*-position of a benzene ring, consistent with the 102-mass difference between **1** and denigrin D [5]. The ^13^C NMR and HSQC spectroscopic data (Table 2) showed the presence of twenty-eight carbon signals, comprising one carbonyl signal (δ_C_ 183.6), twelve quaternary sp^2^ carbon resonances (δ_C_ 124.8, 128.2, 128.4, 130.8, 131.7, 132.0, 140.1, 152.8, 157.1, 157.3, 157.6, 158.1), one quaternary sp^3^ carbon signal (δ_C_ 62.3), ten protonated sp^2^ carbon signals (δ_C_ 115.8, 116.2, 116.8, 116.9, 122.1, 129.0, 130.0, 130.1, 130.9, 132.5), and four methylene carbon resonances (δ_C_ 31.0, 34.9, 38.5, 44.4). The HSQC data determined that the aromatic doublet at δ_H_ 7.07 represented the proton attached to the carbon at δ_C_ 122.2, consistent with a carbon in the α-position to a sulfate group [10], and the HMBC correlation H-22,26/C-24 established that the carbon at δ_C_ 152.9 was attached to a sulfate group [10]. The COSY and HMBC key correlations were analogous to those of denigrin D [5] (Table S1) and confirmed a tyramine unit, two *para*-hydroxybenzyl units, and two *para*-hydroxyphenyl units attached to a pyrrolidone core. In a similar way to denigrin D, the assignments of the four *para*-phenol groups and a *para*-substituted phenyl group were established by combined analysis of characteristic ^13^C chemical shifts, COSY couplings, and HMBC correlations. The HMBC correlations H-35,39/C-33 and H-33/C-2, C-3, C-4, established the presence of ring E, part of a *para*-hydroxybenzyl unit, at C-3, while the correlation H-28,32/C-3 established that ring D, a *para*-hydroxyphenyl group, was also attached at C-3. The COSY correlation H-6/H-7 and the HMBC correlations H-9,13/C-7 and H-6/C-2 confirmed ring A as part of a tyramine moiety attached to N-1, while the HMBC correlations H-16,20/C-14 and H-14/C-5 established ring B as part of the second *para*-hydroxybenzyl unit, attached at C-5. Finally, the chemical shifts of protons and carbons in ring C and the HMBC correlations of H-22,26/C-24 confirmed that the *para*-substituent was a sulfate. Additional HMBC correlations to C-4 confirmed the attachment of ring C at C-4 and thus that **1** was the 24-sulfate of denigrin D. Measurement of the optical rotation of **1** resulted in a value of 0°, suggesting that **1** is present as a racemic mixture, consistent with the results reported for denigrin D [5].

Denigrin I (**2**) was obtained as a light, yellow amorphous solid exhibiting UV absorption maxima at 225 and 278 nm. The IR spectrum showed similar strong absorptions to those of **1** for the hydroxy (3369 cm^−1^) and carbonyl (1663 cm^−1^) groups. HR-ESI-MS returned the [M − Na]^−^ ion at *m/z* 678.1807, corresponding to a molecular formula of C_38_H_32_NO_9_S and indicating that **2** had an identical molecular formula to **1**. The ^13^C NMR and HSQC spectroscopic data (Table 2) indicated the presence of twenty-eight signals, including one carbonyl signal (δ_C_ 182.9), twelve quaternary sp^2^ carbon resonances (δ_C_ 124.9, 126.1, 128.40, 128.41, 130.8, 137.9, 138.7, 153.5, 157.1, 157.2, 157.58, 157.63), one quaternary sp^3^ carbon signal (δ_C_ 62.4), ten protonated sp^2^ carbon resonances (δ_C_ 115.7, 116.0, 116.2, 116.8, 122.8, 128.8, 130.1, 130.3, 131.0, 132.6), and four methylene carbon signals (δ_C_ 31.1, 34.9, 38.6, 44.3). The ^13^C NMR spectrum was almost superimposable on that of **1** (SI Figure S1c,d), with the two signals at δc 122.8 and 153.5 ppm indicating that **2** also contained a sulfate group. As for **1**, the ^1^H NMR and HSQC spectroscopic data of **2** (Table 1) displayed eight methylene signals (δ_H_ 2.18, 2.69, 3.08, 3.09, 3.29, 3.41, 3.68, 3.79) accounting for the four methylene groups and ten aromatic doublets, accounting for a total of 20 protons, indicating five *para*-substituted benzene groups (δ_H_ 6.34, 6.56, 6.63, 6.65, 6.66, 6.68, 6.706, 6.714, 7.39, 7.42), with each doublet displaying a peak shape characteristic of magnetic inequivalence for *para*-disubstituted benzene. The COSY and HMBC key correlations were analogous to those of **1** (Table S1) and confirmed a tyramine unit, two *para*-hydroxybenzyl units, one *para*-hydroxyphenyl unit, and an additional *para*-substituted phenyl unit attached to a pyrrolidone core identically to **1**. The COSY spectrum indicated that the two most downfield signals, characteristic of a sulfated benzene ring, were mutually correlated, and the HMBC correlations of H-28,32/C-3 and H-28,32/C-30 revealed attachment of the sulfated ring D at C-3, indicating that **2** is the 30-sulfate of denigrin D. Measurement of the optical rotation of **2** also resulted in a value of 0°, suggesting that like **1**, **2** is present as a racemic mixture, consistent with the results reported for denigrin D [5].

Denigrin J (**3**) was isolated as a dark green, amorphous solid exhibiting UV absorption maxima at 224 and 273 nm. The IR spectrum showed strong absorptions for the hydroxy (3246 cm^−1^) group with the absence of a carbonyl absorption. HR-ESI-MS yielded the [M − Na]^−^ ion at *m/z* 662.1857, corresponding to a molecular formula of C_38_H_32_NO_8_S. The combined ^13^C NMR and HSQC data (Table 2) indicated the presence of seventeen carbon signals, including eight quaternary sp^2^ carbon resonances (δ_C_ 123.4, 128.3 129.7, 132.8, 136.6, 152.6, 156.1, 156.7), six protonated sp^2^ carbon signals (δ_C_ 115.6, 116.3, 122.5, 130.0, 130.6, 132.5) and three methylene carbon signals (δ_C_ 30.7, 37.7, 47.1). The ^13^C NMR spectroscopic data was almost superimposable on those of denigrin E (**6**) recorded in CD_3_OD (Table 2, SI Figure S6a,b), indicating that the two compounds were closely related, with the key differences being one protonated sp^2^ signal shifted downfield to δc 122.5 ppm instead of 116 ppm, and one quaternary sp^2^ carbon signal at δc 152.6 ppm, instead of δc 157.0 ppm, indicative of a sulfur-bearing carbon. As for **1**, these δ_C_ shifts were characteristic of the presence of a sulfate group in the *para*-position of a benzene ring, consistent with the 102-mass difference between **3** and denigrin E. The ^1^H NMR (Table 1) and HSQC spectra of **3** showed three signals for methylene protons (δ_H_ 2.45, 3.59, 3.85) and six aromatic doublets (δ_H_ 6.61, 6.69, 6.78, 6.92, 6.93, 7.13), with each doublet displaying a peak shape characteristic of magnetic inequivalence for a *para*-disubstituted benzene. The integration of the aromatic protons, allowing that the doublets at 6.92 and 6.93 were integrated together, revealed a ratio of 2:2:1:4:1, respectively, indicating the symmetry of the molecule. The δ_H_ of the doublets at 7.13 and 6.78 ppm were a close match with the aromatic protons on the sulfated benzene ring of dictyodendrin D [10]. The COSY and HMBC key correlations were analogous to those of denigrin E [5] (SI Table S3) and confirmed a tyramine unit, two *para*-hydroxybenzyl units, and two *para*-hydroxyphenyl units attached to a pyrrole core. The HMBC correlation H-22,26/C-4 (H-28,32/C-3) established the *para*-hydroxyphenyl groups at C-3 and C-4, while the HMBC correlations of H-16,20/C-14 (H35,39/C-33) and H-14-C-5 (H-33/C-2) revealed that the *para*-hydroxybenzyl groups were substituted at C-2 and C-5. The COSY correlation H-6/H-7 and the HMBC correlations of H-7/C-9,13 and H-9,13/C-11 indicated the presence of the sulfate group in the *para*-position of the tyramine unit, consistent with the symmetry of the molecule and indicating that **3** is the 11-sulfate of **6**. 

Denigrin K (**4**) was purified as a light green, amorphous solid with identical UV absorption maxima to **3**, IR absorption for an hydroxy (3365 cm^−1^) group, and HR-ESI-MS returned the [M − Na]^−^ ion at *m/z* 662.1855, corresponding to a molecular formula of C_38_H_32_NO_8_S and indicating that **4** had an identical molecular formula to **3**. The ^13^C NMR spectroscopic data (Table 2) showed the presence of twenty-eight signals, suggesting a breach in the symmetry of the molecule. The combined ^13^C NMR and HSQC data confirmed the presence of fourteen quaternary sp^2^ carbon signals (δ_C_ 122.9, 123.4, 128.68, 128.73, 129.4, 130.94, 132.7, 132.8, 135.2, 151.6, 156.2, 156.68, 156.70, 157.0), ten protonated sp^2^ carbon resonances (δ_C_ 115.7, 116.1, 116.30, 116.32, 121.9, 129.87, 129.92, 130.92, 132.0, 132.5), and four methylene carbon signals (δ_C_ 30.57, 30.63, 37.6, 47.3). Two signals characteristic of the presence of a sulfate group were present at δc 121.9 and 151.6 ppm. The ^1^H NMR (Table 1) and HSQC spectra of **4** showed the same three signals for methylene protons (δ_H_ 2.45, 3.58, 3.80) and ten aromatic doublets (δ_H_ 6.62, 6.63, 6.66, 6.70, 6.70, 6.89, 6.91, 6.92, 7.06, 7.12) accounting for 20 protons, indicating that each of the aromatic substituents was *para*-substituted, with each doublet displaying a peak shape characteristic of such magnetic inequivalence. The COSY and HMBC key correlations were analogous to those of **3** (SI Table S3) and confirmed the same tyramine unit, two *para*-hydroxybenzyl units, one *para*-hydroxyphenyl unit, and an additional *para*-substituted phenyl unit linked identically to the pyrrole core. The HMBC correlations of H-28,32/C-3 and H-28,32/C-30 revealed the presence of the sulfate group in the *para*-position of the D ring, consistent with the absence of symmetry and indicating that **4** is the 30-sulfate of **6**. 

Denigrin L (**5**) was obtained as a light blue, amorphous solid exhibiting similar UV absorption maxima and IR absorptions to those of **3** and **4**. HR-ESI-MS returned the [M-Na]^-^ ion at *m/z* 764.1225, corresponding to a molecular formula of C_38_H_31_NO_11_S_2_Na. The ^13^C NMR spectroscopic data (Table 2) showed twenty-seven signals indicating a non-symmetrical compound. The combined ^13^C NMR and HSQC data confirmed the presence of fourteen quaternary sp^2^ carbon resonances (δ_C_ 123.0, 123.5, 128.6, 128.7, 129.3, 132.6, 132.7, 135.1, 136.5, 151.7, 152.5, 156.3, 156.78, 156.83), ten protonated sp^2^ carbon signals (δ_C_ 115.8, 116.37, 116.41, 121.9, 122.5, 129.96, 130.00, 130.4, 132.0, 132.6), and three methylene carbon resonances (δ_C_ 30.7, 37.6, 47.2). The two protonated sp^2^ signals at δc 122.5 and 121.9 and the two quaternary sp^2^ carbon signals at δ_C_ 152.5 and 151.7 ppm indicated the presence of two sulfate groups, consistent with a mass difference of 102 between **3** and **5**. The ^1^H NMR (Table 1) and HSQC spectra of **5** contained four signals for methylene protons (δ_H_ 2.45, 3.61, 3.87, 3.94) and eight aromatic doublets (δ_H_ 6.63, 6.708, 6.710, 6.80, 6.95, 7.09, 7.14, 7.14), confirming the lack of symmetry of the compound, despite the doublet at 6.95 integrating for six protons, with each doublet displaying a peak shape characteristic of magnetic inequivalence for a *para*-substituted benzene. The COSY and HMBC key correlations were analogous to those of **3** and **4** (SI Tables S3 and S4) and confirmed the same tyramine unit, two *para*-hydroxybenzyl units, one *para*-hydroxyphenyl unit, and an additional *para*-substituted phenyl unit linked identically to the pyrrole core. The HMBC correlations of H-28,32/C-3 and H-28,32/C-30 revealed the presence of one sulfate group in the *para*-position of the D ring and the H-7/C-9,3 and H-9,13/C-11 corelations placed the second group in the *para*-position of the A ring; therefore, **5** is the 11- and 30-disulfated analogue of **6**.

The two known compounds, dictyodendrin B and denigrin G, were also identified from the *D.* c.f. *dendyi* extract, with denigrin G isolated as a mixture with dactylpyrrole A, previously reported from the *D*. c.f. *dendyi* extract [9]. Both known compounds displayed NMR and ESI-MS spectroscopic data matching closely with literature values [10,11].

### 2.2. Cytotoxicity Assay

Compounds **1**–**5**, along with six known compounds, namely dictyodendrin C, D, and F, denigrin E, dactylpyrrole A, and lamellarin O1, previously isolated from the same *D*. c.f. *dendyi* sponge extract [9], were assessed for cytotoxic activity towards the human cervical cancer cell line HeLa using an MTT assay. Dictyodendrin B and denigrin G were not present in sufficient quantity to be tested. The tested compounds showed minimal activity (SI Table S6). Dictyodendrin F was the most active of these compounds (IC_50_ 48 µM) but the activity was still only at a level considered inactive by widely accepted standards [2]. Dictyodendrin F has previously been shown to exhibit minimal activity against the P-glycoprotein (P-gp) overexpressing multi-drug resistant variant SW620 Ad300 (IC_50_ > 30 µM), and weak cytotoxicity against the colorectal cancer cell line SW620 (IC_50_ 8.5 µM), showing increased selectivity for the SW620 cell line [11]. Lamellarin O1 was also previously shown to exhibit minimal cytotoxic activity against both SW620 and SW620 Ad300 cells lines (IC_50_ > 30 μM) [12]. 

### 2.3. Oxidative Rearrangement of Denigrin E Derivatives

Pyrrole cores are known to undergo rapid oxidation in the presence of air, leading to a wide array of possible rearrangements [13,14]. In our case, it was observed that **4** underwent a rearrangement within a few days of being dissolved in CD_3_OD and stored in an NMR tube, likely due to exposure to air and possibly to light. The mixture obtained contained in part *p*-hydroxybenzaldehyde, formed by oxidation of the hydroxybenzyl groups in positions 14 and 33, with ESI-MS and ^1^H NMR spectroscopic data analogous to those reported in the literature in CD_3_OD [15]. The oxidative conversion of denigrin E into denigrin D was first reported in 2022 by Chen et al. [6]. The authors examined several oxidants and reported obtaining denigrin D with 61% yield when denigrin E was reacted with *t*-BuOOH at 80 °C in the presence of Mo(CO)_6_. Interestingly, when denigrin E permethyl ether was treated with MoOPH, denigrin D permethyl ether was obtained in 14% yield, along with variable quantities of *p*-anisaldehyde. In our case, traces of the [M + Na]^+^ ion at 724 in positive ion mode and the [M − Na]^−^ ion at 678 in negative ion mode were detected in the decomposition mixture by ESI-MS, possibly corresponding to the formation of **1** and/or **2**. However, neither of the two products could accurately be detected in the mixture by ^1^H NMR spectroscopy, possibly due to low yield of such rearrangements from simple exposure to air. 

Since **3** and **5** both contain a pyrrole core and possess structures closely related to **4**, both could potentially undergo similar oxidative rearrangement. To prevent this, while further purifying the *D*. c.f. *dendyi* extracts, fractions that contained **3**, **4**, or **5** were stored under nitrogen and kept in solution for a minimal amount of time. 

### 2.4. Occurrence of the Sulfate Moiety in Sponge Metabolites

Despite the reasonably high concentration of inorganic sulfate in seawater (~28.7 mM) [16], there have been relatively few reports of secondary metabolites containing a sulfate moiety from sponges. Only 209 sulfated compounds have been reported from sponges, with the majority of these being either sulfated terpenes (75), sterol sulfates (76), or alkaloids (28) [17]. One other sulfated metabolite, a sterol sulfate, has been reported from a New Zealand sponge [18]. Prior to the work described here, pyrrole or pyrrolone alkaloids containing sulfate groups have only been reported from four sponge species. Besides the dictyodendrins mentioned earlier, obtained from *Dictyodendrilla verongiformis* [10], the [2-amino-3-(3′,4′-dihydroxyphenyl) propionic acid] (DOPA)-derived baculiferins A-O were isolated from the Chinese sponge *Iotrochota baculifera* [19], fasciospongines A-C, 19-oxofasciospongine A, and 25-hydroxyhalisulfate 9 were obtained from a Paluan *Fasciospongia* sp. [20,21], and 14-O-sulfate massadine was obtained from an Australian *Axinella* sp. [22].

## 3. Materials and Methods

### 3.1. General Experimental Procedures

UV spectra were measured with an Agilent Cary 300 UV-Vis spectrophotometer (Agilent Technologies, Santa Clara, CA, USA). IR spectra were recorded with a Jasco FT/IR-6X spectrometer (JASCO International Co., Ltd., Tokyo, Japan) using an ATR unit. NMR experiments were performed on a JEOL ECZR 600 MHz spectrometer (JEOL Ltd., Tokyo, Japan) and are referenced to CD_3_OD (^1^H: 3.31 ppm, ^13^C: 49.0 ppm). Delta NMR Data Processing Software (v6.2.0) was used for spectroscopic analyses. The baselines of the ^13^C NMR spectra of **2**, **4**, and **5** were corrected with a polynomial correction and the zero-fill value was increased from 1 to 30 to resolve close signals. The baseline of the ^13^C NMR spectrum of **1** was corrected with a piecewise linear correction. The line broadening on the ^13^C NMR spectrum for **4** was reduced from 2 Hz to 1 Hz to enable resolution of the signal at 130.9 ppm and that on the ^13^C NMR spectrum for **2** was also reduced from 1.4 Hz to 0.4 Hz to enable resolution of the signals at 128.40 and 128.41 ppm. ESI and HR-ESI-MS mass spectra were recorded using a Bruker Daltonics MicrOTOF electrospray ionization mass spectrometer (Bruker, Bremen, Germany). Sodium formate solution was used for calibration. Solid samples were dissolved in a few drops drop of MeOH, and one drop of each sample was added to an Eppendorf tube pre-filled with 1.5 mL of MeOH. The samples were centrifuged before use to ensure separation of undissolved solids. Spectra were recorded with a capillary exit voltage of 150 V in positive ion mode and −150 V in negative ion mode. High performance liquid chromatography (HPLC) was performed using a Shimadzu system (Shimadzu Australasia, Auckland, New Zealand) equipped with a SCL-40 system controller, a LC-40D XS solvent delivery module, a SIL-40C XS auto sampler, a CTO-40C column oven, and an SPD-M40 UV−vis detector, operating under LabSolutions software version 5.118. A Phenomenex Luna C18 column (5 μ, 100 Å, 150 × 4.6 mm, kept at 40 °C) with a gradient elution of H_2_O/MeOH (60:40–47:53, *v*/*v*, containing 0.1% FA) over 34 min at 1 mL/min was used for HPLC purification. Formic acid for analysis (98–100%, ACS) was supplied by Scharlau. Lyophilization of the crude extracts utilized a BÜCHI lyophilizer Lyovapor L-200 (BÜCHI Labortechnik AG, Flawil, Swizerland). Solvent was removed under reduced pressure using a BÜCHI Rotavapor *011* Rotary Evaporator (BÜCHI Labortechnik AG, Flawil, Swizerland). Condenser system combined with a Thermo Haake K10 circulating chiller. Solvents used for general purposes were purchased as drum grade and distilled in the laboratory. Water used for chemical analyses was type 1 grade. The reversed phase purifications were carried out with C18 YMC-gel ODS-A (YMC America, Devens, MA, USA) and gel filtrations were carried out on Sephadex LH-20 (Sigma, St Louis, MO, USA). 

### 3.2. Animal Material

*Dictyodendrilla* c.f. *dendyi* was collected at depths between two to six meters using free diving and scuba diving between March and December 2022 at Dive Crescent (37°40′44.6″ S 176°10′15.8″ E), a sponge meadow within Tauranga Harbour, New Zealand, and kept frozen at −20 °C until extraction. The sponge displays skeletal similarities to *Dictyodendrilla dendyi* (Bergquist, 1996) that has been recorded from the North Island of New Zealand [8] and is pending full taxonomic investigation. A voucher specimen is kept at the University of Waikato Coastal Marine Field Station in Tauranga, New Zealand.

### 3.3. Cell Line and Cell Culture

The human cervical cancer cell line, HeLa, was purchased from the American Tissue Culture Collection (ATCC) (Number CCL-2). The cells were grown in Minimum Essential Medium (MEM, Gibco) supplemented with 100× units of penicillin/streptomycin (Gibco, (Life Technologies Corporation, Grand Island, NY, USA) and 10% foetal bovine serum (FBS, Gibco). Culturing was carried out using T-25 and T-75 treated flasks (Biofil) in a standard humidified incubator (Sanyo™) at 37 °C with 5% CO_2_. Subculturing of cells was performed every 4 days when near confluency..

### 3.4. MTT Cytotoxicity Assay

The MTT assay was used to evaluate the cytotoxicity of compounds against cancer cells by two independent experiments. Near-confluent cells in log-phase were harvested and seeded evenly in 96-well cell culture plates (Biofil) at a density of 1.5 × 10^5^ cells per well, each well containing 200 μL cell culture medium. The plates were then incubated for 24 h (37 °C; 5% CO_2_) to allow for cell adhesion. The media was aspirated, and the cells were washed with 200 μL PBS (Gibco) before adding 150 or 180 μL of adjusted media to the cell wells. Compounds were dissolved in 5% aqueous DMSO (*v*/*v*) and diluted within the range of 125–2000 µM. Aliquots (20 or 50 µL) of each dilution, or of 5% aqueous DMSO for the control wells, were added in duplicate. After 68 h of incubation (37 °C; 5% CO_2_), the plates were spun at 80 rcf for five minutes at room temperature before replacing the media with 200 µL of fresh media and 10 µL of 5 mg/mL MTT reagent (Sigma, St. Louis, MO, USA) in PBS. Following MTT addition, the plates were incubated for an additional 4 h (37 °C ; 5% CO_2_). The plates were then spun again for 5 min, and the MTT media was replaced with 100 µL of prewarmed solubilizing solution, made up by dissolving 10 g of SDS in 23 mL sterile type 1 water, followed by the addition of 1 mL of 1M HCl and glacial acetic acid to adjust the pH to 4.7. Formazan crystals were dissolved by shaking on an Alphatech Torrey Pines orbital mixer for 5 min, and the plates were further incubated for 30 min. Once homogenized, the samples were spectrophotometrically measured on a plate reader (Bio-Rad 680) at 570 nm, with a background absorbance reading at 655 nm. The IC_50_ value was obtained graphically with Microsoft Office Excel version 2404 as the concentration of the compound required for 50% inhibition of the cancer cells. 

### 3.5. Extraction and Isolation

A portion of the frozen *D.* c.f. *dendyi* sponge (80 g) was soaked overnight in MeOH/CH_2_Cl_2_ (3:1), then blended, and filtered under vacuum. The extraction steps were repeated several times with a 1 h soaking time until the filtrate was colorless. The filtrates were combined, and the solvent was removed under reduced pressure. The crude extract was then lyophilized to afford 3.1 g of black powder, which was purified by bench column chromatography on reversed-phase C18 with a steep, stepped gradient from H_2_O to MeOH to CH_2_Cl_2_. The fraction that eluted with H_2_O/MeOH (3:7) (77 mg) was further purified separately on Sephadex LH-20 with 100% MeOH. Several fractions were then recombined and purified further on Sephadex LH-20 with H_2_O/MeOH (1:1) to afford denigrin L (**5**, 1.2 mg). 

Several minor compounds were also detected by ESI-MS, and to afford their isolation and characterization, a larger scale extraction of the *D.* c.f. *dendyi* sponge (417 g) was carried out, following identical steps as described above, to yield 13.2 g of crude extract. The extract was divided into three equal parts and each part was purified separately by bench column chromatography on reversed-phase C18 with a steep, stepped gradient from H_2_O to MeOH to CH_2_Cl_2_. Fractions that eluted with similar H_2_O/MeOH ratios were then recombined, leading to seven different fractions (A–G) that were purified separately by bench column chromatography on reversed-phase C18 with a steep, stepped gradient from H_2_O to MeOH to CH_2_Cl_2_. Early eluting fractions from each C18 column (from H_2_O/MeOH 6:4 to H_2_O/MeOH 3:7) were then further purified separately by Sephadex LH-20 with H_2_O/MeOH (1:1). Similar fractions were recombined and further purified by Sephadex LH-20 with H_2_O/MeOH (7:3) (23 mg) to afford a mixture of **1** and **2** (3.4 mg). This mixture was then purified by HPLC using a Phenomenex Luna C18 column (5 μ, 100 Å, 150 × 4.6 mm, kept at 40 °C) with a gradient elution of H_2_O/MeOH (60:40–47:53, *v*/*v*, containing 0.1% FA) over 34 min at 1 mL/min to obtain denigrin H (**1**, 1.2 mg) and I (**2**, 0.8 mg). From the initial seven fractions, fraction A, which eluted with H_2_O/MeOH (1:1) (537 mg), was further purified by bench column chromatography on reversed-phase C18 with a steep, stepped gradient as above. The fraction that eluted with H_2_O/MeOH (4:6) (57.3 mg) was purified on Sephadex LH-20 with H_2_O/MeOH (1:1) to afford denigrin J (**3**, 2.1 mg). Two later eluting fractions from the same Sephadex LH-20 column were recombined and purified further by Sephadex LH-20 with H_2_O/MeOH (3:7) to afford dictyodendrin B (0.2 mg), along with a mixture (1.5 mg) of denigrin G and dactylpyrrole A in two later eluting fractions. 

Of the initial seven fractions, fraction B, which also eluted with H_2_O/MeOH (1:1) (233 mg), was further purified by bench column chromatography on reversed-phase C18 with a similar gradient. Three fractions that eluted from H_2_O/MeOH (1:1) to H_2_O/MeOH (3:7) were recombined and purified on Sephadex LH-20 with H_2_O/MeOH (1:1) to afford denigrin K (**4**, 2 mg). Fractions containing either **3**, **4**, or **5** were stored dry in vials conditioned under nitrogen gas. For NMR analysis, the compounds were dissolved in CD_3_OD, placed into NMR tubes conditioned under nitrogen gas, and kept in solution for a minimal amount of time.

Denigrin H (**1**): light yellow amorphous solid; [α]^20^_D_ 0° (MeOH); UV (MeOH) *λ*_max_ (log *ε*) 226 nm (4.42), 279 (4.09) nm; IR (MeOH) ν_max_ 3369, 1663, 1590, 1515, 1442, 1351, 1238, 1175 cm^−1^; ^1^H NMR, Table 1; ^13^C NMR, Table 2; ESI(+)MS *m/z* 724 [M + Na]^+^; ESI(−)MS *m/z* 678 [M − Na]^−^, 700 [M − H]^−^, 1379 [2M − Na]^−^; HR-ESI-MS *m/z* 678.1803 [M − Na]^−^ (calcd for C_38_H_32_NO_9_S, 678.1803).

Denigrin I (**2**): light yellow amorphous solid; [α]^20^_D_ 0° (MeOH); UV (MeOH) *λ*_max_ (log *ε*) 225 nm (4.38), 278 (4.00) nm; IR (MeOH) ν_max_ 3369, 1665, 1593, 1514, 1440, 1353, 1240, 1173 cm^−1^; ^1^H NMR, Table 1; ^13^C NMR, Table 2; ESI(+)MS *m/z* 724 [M + Na]^+^; ESI(−)MS *m/z* 678 [M − Na]^−^, 700 [M − H]^−^; HR-ESI-MS *m/z* 678.1807 [M − Na]^−^ (calcd for C_38_H_32_NO_9_S, 678.1803).

Denigrin J (**3**): dark green amorphous solid; UV (MeOH) *λ*_max_ (log *ε*) 224 nm (4.53), 276 (4.10) nm; IR (MeOH) ν_max_ 3246, 1594, 1510, 1436, 1360, 1230, 1169 cm^−1^; ^1^H NMR, Table 1; ^13^C NMR, Table 2; ESI(+)MS *m/z* 708 [M + Na]^+^; ESI(−)MS *m/z* 662 [M − Na]^−^; HR-ESI-MS *m/z* 662.1857 [M − Na]^−^ (calcd for C_38_H_32_NO_8_S, 662.1849).

Denigrin K (**4**): light green amorphous solid; UV (MeOH) *λ*_max_ (log *ε*) 224 nm (4.40), 277 (4.05) nm; IR (MeOH) ν_max_ 3365, 1594, 1513, 1431, 1356, 1238, 1171 cm^−1^; ^1^H NMR, Table 1; ^13^C NMR, Table 2; ESI(+)MS *m/z* 708 [M + Na]^+^; ESI(−)MS *m/z* 662 [M − Na]^−^; HR-ESI-MS *m/z* 662.1855 [M − Na]^−^ (calcd for C_38_H_32_NO_8_S, 662.1849).

Denigrin L (**5**): light blue amorphous solid; UV (MeOH) *λ*_max_ (log *ε*) 223 nm (4.35), 277 (4.00) nm; IR (MeOH) ν_max_ 3367, 1579, 1508, 1414, 1355, 1236, 1169 cm^−1^; ^1^H NMR, Table 1; ^13^C NMR, Table 2; ESI(+)MS *m/z* 810 [M + Na]^+^; ESI(−)MS *m/z* 370 [M/2 − Na]^−^; 764 [M − Na]^−^; HR-ESI-MS *m/z* 764.1225 [M − Na]^−^ calcd for C_38_H_31_NO_11_S_2_Na, 764.1242).

## 4. Conclusions

Five new sulfated arylpyrrole and arylpyrrolone alkaloids, denigrins H–L (**1–5**), along with two known compounds, dictyodendrin B and denigrin G, were isolated from an extract of a New Zealand *D*. c.f. *dendyi* marine sponge. Denigrins H–L (**1–5**) represent the first examples of sulfated denigrins, belonging to a rare class of highly substituted pyrrole and pyrrolidone alkaloids. Compounds **1**–**5**, along with dictyodendrins C, D, and F, denigrin E, dactylpyrrole A, and lamellarin O1 were inactive towards the human cervical cancer cell line HeLa. 

## Figures and Tables

**Table 1:** ^1^H NMR data of compounds **1**–**5** in CD_3_OD ^a^.

	1	2	3	4	5
No.	δ_H_, Mult (J in Hz)	δ_H_, Mult (J in Hz)	δ_H_, Mult (J in Hz)	δ_H_, Mult (J in Hz)	δ_H_, Mult (J in Hz)
6	3.41, m 3.11 ^b^, m	3.41, m 3.08 ^b^, m	3.59, m	3.58, m	3.61, m
7	2.70, m 2.19, ddd (13.2, 8.9, 4.7)	2.69, m 2.18, ddd (13.1, 8.7, 4.8)	2.45, m	2.45, m	2.45, m
9/13	6.712 ^c^, d (8.5)	6.706 ^c^, d (8.4)	6.78, d (8.5)	6.66, d (9.0)	6.80, d (8.6)
10/12	6.65, d (8.5)	6.65, d (8.6)	7.13, d (8.4)	6.63, d (8.3)	7.14 ^d^, d (8.6)
14	3.77, d (17.0) 3.28 ^e^, m	3.79, d (16.9) 3.29 ^e^, m	3.85, s	3.80, s	3.94, s
16/20	6.34, d (8.4)	6.34, d (8.3)	6.93, d (8.6)	6.89 ^f^, d (8.3)	6.95 ^g^, d (8.6)
17/19	6.705 ^c^, d (8.6)	6.714 ^c^, d (8.4)	6.69, d (8.5)	6.70 ^h^, d (9.0)	6.710 ^c, i^, d (8.6)
22/26	6.79, d (8.9)	6.66, d (8.9)	6.92, d (8.6)	6.92, d (9.0)	6.95 ^g^, d (8.6)
23/25	7.07, d (8.9)	6.56, d (8.7)	6.61, d (8.7)	6.62, d (8.3)	6.63, d (8.7)
28/32	7.25, d (8.8)	7.42, d (9.1)	6.92, d (8.6)	7.06, d (8.6)	7.09, d (8.8)
29/31	6.87. d (8.8)	7.39, d (9.0)	6.61, d (8.7)	7.12, d (8.3)	7.14 ^d^, d (8.6)
33	3.64, d (12.9) 3.09 ^b^, d (12.7)	3.68, d (12.9) 3.09 ^b^, d (12.7)	3.85, s	3.80, s	3.87, s
35/39	6.72, d (8.7)	6.68, d (8.6)	6.93, d (8.6)	6.91 ^f^, d (8.3)	6.95 ^g^, d (8.6)
36/38	6.64, d (8.6)	6.63, d (8.7)	6.69, d (8.5)	6.69 ^h^, d 8.3)	6.708 ^c, i^, d (8.6)

^a^ recorded at 600 MHz, ^b^ signal overlap, ^c^ values given to 3 decimal places to separate signals, ^d, f–i^ assignment interchangeable, ^e^ signal partially obscured.

**Table 2:** ^13^C NMR data of compounds **1**–**6** in CD_3_OD ^a^.

	1	2	3	4	5	6
No.	δ_C_, Type	δ_C_, Type	δ_C_, Type	δ_C_, Type	δ_C_, Type	δ_C_, Type
2	183.6, C	182.9, C	128.3, C	128.73 ^b, c^, C	128.6 ^d^, C	128.3, C
3	62.3, C	62.4, C	123.4, C	122.9, C	123.0, C	123.3, C
4	124.8, C	124.9, C	123.4, C	123.4, C	123.5, C	123.3, C
5	139.9, C	138.7, C	128.3, C	128.68 ^b, c^, C	128.7 ^d^, C	128.3, C
6	44.4, CH_2_	44.3, CH_2_	47.1, CH_2_	47.3, CH_2_	47.2, CH_2_	47.3, CH_2_
7	34.9, CH_2_	34.9, CH_2_	37.7, CH_2_	37.6, CH_2_	37.6, CH_2_	37.7, CH_2_
8	130.8, C	130.6, C	136.6, C	130.94 ^c^, C	136.5, C	131.0, C
9/13	130.9, CH	131.0, CH	130.6, CH	130.92 ^c^, CH	130.4, CH	130.9, CH
10/12	116.2, CH	116.2, CH	122.5, CH	116.1, CH	122.5, CH	116.1, CH
11	157.1, C	157.1, C	152.6, C	157.0, C	152.5, C	157.0, C
14	31.0, CH_2_	31.1, CH_2_	30.7, CH_2_	30.63 ^c, e^, CH_2_	30.7, CH_2_	30.7, CH_2_
15	128.2, C	128.40 ^c, f^, C	132.8, C	132.7 ^g^, C	132.7 ^h^, C	132.9, C
16/20	130.1, CH	130.1, CH	130.0, CH	129.92 ^c, i^, CH	130.00 ^c, j^, CH	129.9, CH
17/19	116.9, CH	116.8, CH	116.3, CH	116.32 ^c, k^, CH	116.41 ^c, l^, CH	116.3, CH
18	157.3, C	157.2, C	156.7, C	156.68 ^c, m^, C	156.83 ^c, n^, C	156.7, C
21	131.7, C	126.1, C	129.7, C	129.4, C	129.3, C	129.7, C
22/26	130.0, CH	130.3, CH	132.5, CH	132.5, CH	132.6, CH	132.5, CH
23/25	122.1, CH	116.0, CH	115.6, CH	115.7, CH	115.8, CH	115.6, CH
24	152.8, C	157.63 ^c^, C	156.1, C	156.2, C	156.3, C	156.1, C
27	132.0, C	137.9, C	129.7, C	135.2, C	135.1, C	129.7, C
28/32	129.0, CH	128.8, CH	132.5, CH	132.0, CH	132.0, CH	132.5, CH
29/31	116.8, CH	122.8, CH	115.6, CH	121.9, CH	121.9, CH	115.6, CH
30	158.1, C	153.5, C	156.1, C	151.6, C	151.7, C	156.0, C
33	38.5, CH_2_	38.6, CH_2_	30.7, CH_2_	30.57 ^c, e^, CH_2_	30.7, CH_2_	30.7, CH_2_
34	128.4, C	128.41^c, f^, C	132.8, C	132.8 ^g^, C	132.6 ^h^, C	132.9, C
35/39	132.5, CH	132.6, CH	130.0, CH	129.87 ^c, i^, CH	129.96 ^c, j^, CH	129.9, CH
36/38	115.8, CH	115.7, CH	116.3, CH	116.30 ^c, k^, CH	116.37 ^c, l^, CH	116.3, CH
37	157.6, C	157.58 ^c^, C	156.7, C	156.70 ^c, m^, C	156.78 ^c, n^, C	156.7, C

^a^ recorded at 150 MHz, ^b, d–n^ assignment interchangeable, ^c^ values given to 2 decimal places to separate signals.

## Data Availability

Data is contained within the article or Supplementary Materials.

## Supplementary Materials

The following supporting information can be downloaded at: https://www.mdpi.com/article/10.3390/md22050231/s1. Tables S1–S5: NMR data of compounds **1**–**5**; Table S6: IC_50_ values for cytotoxicity assay; Figures S1–S5: 1D and 2D NMR spectra and HR-ESI-MS data of compounds **1**–**5**; Figure S6: ^1^H and ^13^C NMR spectrum of compound **6**; Figures S7–S11: ^1^H NMR spectra of dictyodendrin C, D, and F, dactylpyrrole A, and lamellarin O1.
