# Supplementary material for: Denigrins H–L: Sulfated Derivatives of Denigrins D and E from a New Zealand Dictyodendrilla c.f. dendyi Marine Sponge
Source: Mar Drugs. 2024 May 20;22(5):231. doi: 10.3390/md22050231 (PMC11122787; doi:10.3390/md22050231)
Supplement: Supplementary file 1 [file marinedrugs-22-00231-s001.zip › marinedrugs-3013313-supplementary.pdf]

**Electronic Supplementary Information**

Marine Drugs

# Denigrins H-L: Sulfated Derivatives of Denigrins D and E from a New Zealand *Dictyodendrilla* c.f. *dendyi* Marine Sponge

Lauren Gris[1], Michèle R. Prinsep[1*], Linda M. Peters[2] and Christopher N. Battershill[3]

[1] *Chemistry and Applied Physics, School of Science, University of Waikato, Private Bag 3105, Hamilton 3240, New Zealand.*

[2] *Biomedical, Molecular and Cellular Biology, School of Science, University of Waikato, Private Bag 3105, Hamilton 3240, New Zealand.*

[3] *University of Waikato Coastal Marine Field Station, 58 Cross Road, Sulphur Point, Tauranga 3110, New Zealand.*

[*]Corresponding author: michele.prinsep@waikato.ac.nz

Contents

**Table S1**. $^{1}$H and $^{13}$C NMR data of denigrin H (**1**)....4

**Table S2.** $^{1}$H and $^{13}$C NMR data of denigrin I (**2**)....5

**Table S3.** $^{1}$H and $^{13}$C NMR data of denigrin J (**3**)....6

**Table S4.** $^{1}$H and $^{13}$C NMR data of denigrin K (**4**)....7

**Table S5**. $^{1}$H and $^{13}$C NMR data of denigrin L (**5**) ....8

**Figure S1a.** $^{1}$H NMR (methanol-d4, 600 MHz) spectrum of denigrin H (**1**) ....9

**Figure S1b.** $^{13}$C NMR (methanol-d4, 150 MHz) spectrum of denigrin H (**1**) ....10

**Figure S1c.** $^{13}$C NMR (methanol-d4, 150 MHz) spectrum expansion 1 of denigrin H (**1**)....11

**Figure S1d.** $^{13}$C NMR (methanol-*d4*, 150 MHz) spectrum expansion 2 of denigrin H (**1**) ....12

**Figure S1e.** COSY (methanol-*d4*) spectrum of denigrin H (**1**) ....13

**Figure S1f.** COSY (methanol-*d4*) spectrum of denigrin H (**1**)....14

**Figure S1g.** gHSQC (methanol-*d4*) spectrum of denigrin H (**1**)....15

**Figure S1h.** gHMBC (methanol-*d4*) spectrum of denigrin H (**1**)....16

**Figure S1i.** gHMBC (methanol-*d4*) spectrum expansion of denigrin H (**1**)....17

**Figure S1j**. HRESIMS spectrum of denigrin H (**1**) in negative ion mode ........ 18
**Figure S2a.** $^{1}$H NMR (methanol-*d4*, 600 MHz) spectrum of denigrin I (**2**) ........ 19
**Figure S2b.** $^{13}$C NMR (methanol-*d4*, 150 MHz) spectrum of denigrin I (**2**) ........ 20
**Figure S2c.** $^{13}$C NMR (methanol-*d4*, 150 MHz) spectrum expansion of denigrin I (**2**) ........ 21
**Figure S2d.** COSY (methanol-*d4*) spectrum of denigrin I (**2**) ........ 22
**Figure S2e**. gHSQC (methanol-d4) spectrum of denigrin I (**2**) ........ 23
**Figure S2f.** gHMBC (methanol-*d4*) spectrum of denigrin I (**2**) ........ 24
**Figure S2g.** gHMBC (methanol-*d4*) spectrum expansion of denigrin I (**2**) ........ 25
**Figure S2h**. HRESIMS spectrum of denigrin I (**2**) in negative ion mode ........ 26
**Figure S3a.** $^{1}$H NMR (methanol-*d4*, 600 MHz) spectrum of denigrin J (**3**) ........ 27
**Figure S3b.** $^{13}$C NMR (methanol-*d4*, 150 MHz) spectrum of denigrin J (**3**) ........ 28
**Figure S3c.** $^{13}$C NMR (methanol-*d4*, 150 MHz) spectrum expansion of denigrin J (**3**) ........ 29
**Figure S3d.** COSY (methanol-*d4*) spectrum of denigrin J (**3**) ........ 30
**Figure S3e.** gHSQC (methanol-*d4*) spectrum of denigrin J (**3**) ........ 31
**Figure S3f.** gHMBC (methanol-*d4*) spectrum of denigrin J (**3**) ........ 32
**Figure S3g.** gHMBC (methanol-*d4*) spectrum expansion of denigrin J (**3**) ........ 33
**Figure S3h**. HRESIMS spectrum of denigrin J (**3**) in negative ion mode ........ 34
**Figure S4a.** $^{1}$H NMR (methanol-*d4*, 600 MHz) spectrum of denigrin K (**4**) ........ 35
**Figure S4b.** $^{13}$C NMR (methanol-*d4*, 150 MHz) spectrum of denigrin K (**4**) ........ 36
**Figure S4c.** $^{13}$C NMR (methanol-*d4*, 150 MHz) spectrum expansion 1 of denigrin K (**4**) ........ 37
**Figure S4d.** $^{13}$C NMR (methanol-*d4*, 150 MHz) spectrum expansion 2 of denigrin K (**4**) ........ 38
**Figure S4e.** COSY (methanol-*d4*) spectrum of denigrin K (**4**) ........ 39
**Figure S4f.** gHSQC (methanol-*d4*) spectrum of denigrin K (**4**) ........ 40
**Figure S4g.** gHMBC (methanol-*d4*) spectrum of denigrin K (**4**) ........ 41
**Figure S4h.** gHMBC (methanol-*d4*) spectrum expansion of denigrin K (**4**) ........ 42
**Figure S4i**. HRESIMS spectrum of denigrin K (**4**) in negative ion mode ........ 43
**Figure S5a.** $^{1}$H NMR (methanol-*d4*, 600 MHz) spectrum of denigrin L (**5**) ........ 44
**Figure S5b.** $^{13}$C NMR (methanol-*d4*, 150 MHz) spectrum of denigrin L (**5**) ........ 45
**Figure S5c.** $^{13}$C NMR (methanol-*d4*, 150 MHz) spectrum expansion of denigrin L (**5**) ........ 46
**Figure S5d.** COSY (methanol-*d4*) spectrum of denigrin L (**5**) ........ 47
**Figure S5e.** gHSQC (methanol-*d4*) spectrum of denigrin L (**5**) ........ 48
**Figure S5f.** gHMBC (methanol-*d4*) spectrum of denigrin L (**5**) ........ 49
**Figure S5g.** gHMBC (methanol-*d4*) spectrum expansion of denigrin L (**5**) ........ 50

**Figure S5h**. HRESIMS spectrum of denigrin L (**5**) in negative ion mode ................................................51
**Figure S6a.** $^{1}$H NMR (methanol-*d4*, 600 MHz) spectrum of denigrin E (**6**)............................................52
**Figure S6b.** $^{13}$C NMR (methanol-*d4*, 150 MHz) spectrum of denigrin E (**6**)...........................................53
**Figure S6c.** $^{13}$C NMR (methanol-*d4*, 150 MHz) spectrum expansion of denigrin E (**6**)............................54
**Figure S7.** $^{1}$H NMR (methanol-*d4*, 600 MHz) spectrum of dictyodendrin C..........................................55
**Figure S8.** $^{1}$H NMR (methanol-*d4*, 600 MHz) spectrum of dictyodendrin D ..........................................56
**Figure S9.** $^{1}$H NMR (methanol-*d4*, 600 MHz) spectrum of dictyodendrin F ...........................................57
**Figure S10.** $^{1}$H NMR (methanol-*d4*, 600 MHz) spectrum of dactypyrrole A...........................................58
**Figure S11**. $^{1}$H NMR (methanol-*d4*, 600 MHz) spectrum of lamellarin O1 ............................................59
**Table S6**. Cytotoxicity assay - $IC_{50}$ values for compounds **1**-**6**, dictyodendrin C, D, and F, dactylpyrrole A and lamellarin O1 ..........................................................................................................60

**Table S1**. $^{1}H$ and $^{13}C$ NMR data of denigrin H (**1**)

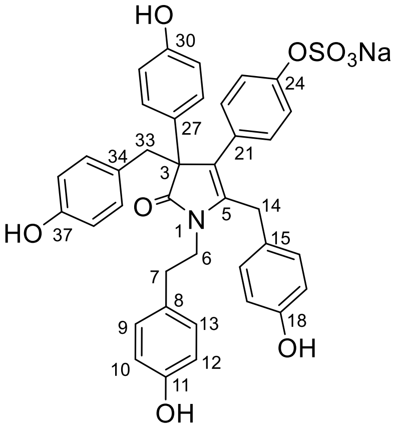

| **No.** | **$\delta_C$ [a]** | **$^{1}H$ $\delta_H$, (mult, *J* in Hz) [b]** | **HMBC ($^{1}H$ to $^{13}C$)** | **COSY** |
|---|---|---|---|---|
| **2** | 183.6 | | | |
| **3** | 62.3 | | | |
| **4** | 124.8 | | | |
| **5** | 139.9 | | | |
| **6** | 44.4 | 3.41 (1H, m)<br>3.11 [c] (1H, m) | <br>C-2 | H-6, H-7<br>H-7 |
| **7** | 34.9 | 2.70 (1H, m)<br>2.19 (1H, m) | C-6, C-9/13<br>C-8, C-9/13 | H-7 |
| **8** | 130.8 | | | |
| **9/13** | 130.9 | 6.712 [d] (2H, d, 8.5) | C-7, C-11 | H-9/13 |
| **10/12** | 116.2 | 6.65 (2H, d, 8.5) | C-8 | |
| **11** | 157.1 | | | |
| **14** | 31.0 | 3.77 (1H, d, 17.0)<br>3.28 [e] (1H, m) | C-5, C-15, C-16/20<br>C-4, C-5, C-15, C-16/20 | H-14 |
| **15** | 128.2 | | | |
| **16/20** | 130.1 | 6.34 (2H, d, 8.4) | C-14, C-18 | |
| **17/19** | 116.9 | 6.705 [d] (2H, d, 8.6) | C-15 | H-16/20 |
| **18** | 157.3 | | | |
| **21** | 131.7 | | | |
| **22/26** | 130.0 | 6.79 (2H, d, 8.9) | C-4, C-24 | |
| **23/25** | 122.1 | 7.07 (2H, d, 8.9) | C-24, C-21 | H-22/26 |
| **24** | 152.8 | | | |
| **27** | 132.0 | | | |
| **28/32** | 129.0 | 7.25 (2H, d, 8.8) | C-3, C-30 | H-29/31 |
| **29/31** | 116.8 | 6.87 (2H, d, 8.8) | C-27 | |
| **30** | 158.1 | | | |
| **33** | 38.5 | 3.64 (1H, d, 12.9)<br>3.09 [c] (1H, d (12.7) | C-3, C-4, C-34, C-35/39<br>C-2, C-3, C-34, C-35/39 | H-33 |
| **34** | 128.4 | | | |
| **35/39** | 132.5 | 6.72 (2H, d, 8.7) | C-33, C-37 | H-36/38 |
| **36/38** | 115.8 | 6.64 (2H, d, 8.6) | C-34 | |
| **37** | 157.6 | | | |

[a] recorded at 150 MHz in $CD_3OD$, [b] recorded at 600 MHz in $CD_3OD$, [c] signal overlap, [d] values given to 3 decimal places to separate signals, [e] signal partially obscured

**Table S2.** $^{1}H$ and $^{13}C$ NMR data of denigrin I (**2**)

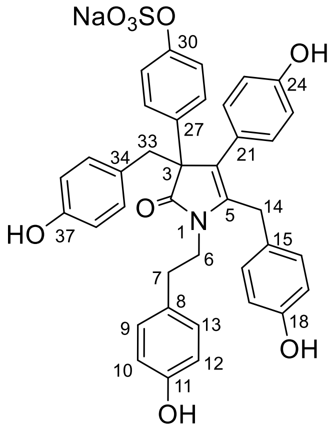


| No. | $\delta_C$ [a] | $^{1}H$ <br> $\delta_H$, (mult, *J* in Hz) [b] | HMBC ($^{1}H$ to $^{13}C$) | COSY |
|---|---|---|---|---|
| **2** | 182.9 | | | |
| **3** | 62.4 | | | |
| **4** | 124.9 | | | |
| **5** | 138.7 | | | |
| **6** | 44.3 | 3.41 (1H, m)<br>3.08 [c] (1H, m) | <br>C-2 | H-6, H-7<br>H-7 |
| **7** | 34.9 | 2.69 (1H, m)<br>2.18 (1H, ddd) | C-9/13<br>C-6, C-9/13 | H-7 |
| **8** | 130.6 | | | |
| **9/13** | 131.0 | 6.706 [d] (2H, d, 8.4) | C-7, C-11 | H-9/13 |
| **10/12** | 116.2 | 6.65 (2H, d, 8.6) | C-8 | |
| **11** | 157.1 | | | |
| **14** | 31.1 | 3.79 (1H, d, 16.9)<br>3.29 [e] (1H, m) | C-5, C-15, C-16/20<br>C-4, C-5, C-15, C-16/20 | H-14 |
| **15** | 128.40 [f, g] | | | |
| **16/20** | 130.1 | 6.34 (2H, d, 8.3) | C-14, C-18 | |
| **17/19** | 116. 8 | 6.714 [d] (2H, d, 8.4) | C-15 | H-16/20 |
| **18** | 157.2 | | | |
| **21** | 126.1 | | | |
| **22/26** | 130.3 | 6.66 (2H, d, 8.9) | C-4, C-24 | H-22/26 |
| **23/25** | 116.0 | 6.56 (2H, d, (8.7) | C-21, C-24 | |
| **24** | 157.63 [g] | | | |
| **27** | 137.9 | | | |
| **28/32** | 128.8 | 7.42 (2H, d, 9.1) | C-3, C-30 | H-29/31 |
| **29/31** | 122.8 | 7.39 (2H, d, 9.0) | C-27, C-30 | |
| **30** | 153.5 | | | |
| **33** | 38.6 | 3.68 (1H, d, 12.9)<br>3.09 [c] (1H, d, 12.7) | C-3, C-4, C-34, C-35/39<br>C-2, C-3, C-35/39, C-34 | H-33 |
| **34** | 128.41 [f, g] | | | |
| **35/39** | 132.6 | 6.68 (2H, d, 8.6) | C-33 | H-36/38 |
| **36/38** | 115.7 | 6.63 (2H, d, 8.7) | C-34, C-37 | |
| **37** | 157.58 [g] | | | |

[a] recorded at 150 MHz in $CD_3OD$, [b] recorded at 600 MHz in $CD_3OD$, [c] signal overlap, [d] values given to 3 decimal places to separate signals, [e] signal partially obscured, [f] assignment interchangeable, [g] values given to 2 decimal places to separate signals

**Table S3.** $^{1}H$ and $^{13}C$ NMR data of denigrin J (**3**)

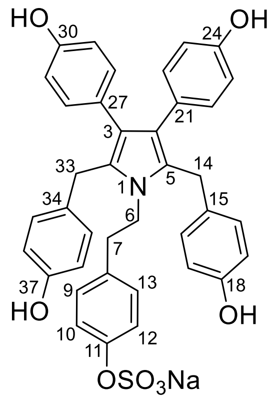


| No. | $\delta_C$ [a] | $^{1}H$ $\delta_H$, (mult, *J* in Hz) [b] | HMBC ($^{1}H$ to $^{13}C$) | COSY |
|---|---|---|---|---|
| **2/5** | 128.3 | | | |
| **3/4** | 123.4 | | | |
| **6** | 47.1 | 3.59 (2H, m) | C-7, C-2/5 | H-7 |
| **7** | 37.7 | 2.45 (2H, m) | C-6, C-9/13, C-8 | |
| **8** | 136.6 | | | |
| **9/13** | 130.6 | 6.78 (2H, d, 8.5) | C-7, C-11 | |
| **10/12** | 122.5 | 7.13 (2H, d 8.4) | C-8, C-11 | H-9/13 |
| **11** | 152.6 | | | |
| **14/33** | 30.7 | 3.85 (4H, s) | C-3/4, C-2/5, C-16/20–35/39, C-15/34 | |
| **15/34** | 132.8 | | | |
| **16/20–35/39** | 130.0 | 6.93 (4H, d, 8.6) | C-14/33, C-18/37 | H16/20-35/39 |
| **17/19–36/38** | 116.3 | 6.69 (4H, d, 8.5) | C-15/34, C-18/37 | |
| **18/37** | 156.7 | | | |
| **21/27** | 129.7 | | | |
| **22/26–28/32** | 132.5 | 6.92 (4H, d, 8.6) | C-3/5, C-24/30 | H23/25-29/31 |
| **23/25–29/31** | 115.6 | 6.61 (4H, d, 8.7) | C-21/27, C-24/30 | |
| **24/30** | 156.1 | | | |

[a] recorded at 150 MHz in $CD_3OD$, [b] recorded at 600 MHz in $CD_3OD$

**Table S4.** $^{1}$H and $^{13}$C NMR data of denigrin K (**4**)

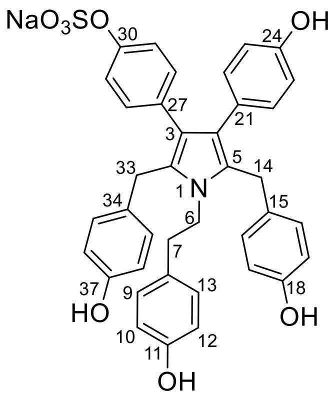


| **No.** | **δ$_C$ [a]** | **$^{1}$H δ$_H$ (mult, *J* in Hz) [b]** | **HMBC ($^{1}$H to $^{13}$C)** | **COSY** |
|---|---|---|---|---|
| **2** | 128.73 [c, d] | | | |
| **3** | 122.9 | | | |
| **4** | 123.4 | | | |
| **5** | 128.68 [c, d] | | | |
| **6** | 47.3 | 3.58 (2H, m) | C-2, C-7, C-5 | H-7 |
| **7** | 37.6 | 2.45 (2H, m) | C-6, C-9/13 | |
| **8** | 130.94 [c] | | | |
| **9/13** | 130.92 [c] | 6.66 (2H, d, 9.0) | C-7, C-11 | |
| **10/12** | 116.1 | 6.63 (2H, d, 8.3) | C-8, C-11 | |
| **11** | 157.0 | | | |
| **14/33** | 30.57<br>30.63 | 3.80 (4H, s) | C-2, C-3, C-4, C-5, C-15,<br>C-16/20, C-35/39, C-34 | |
| **15** | 132.7 [e] | | | |
| **16/20** | 129.92 [c, f] | 6.89 [g] (2H, d, 8.3) | C-14/33, C-18 | H17/19 |
| **17/19** | 116.32 [c, h] | 6.70 [i] (2H, d, 9.0) | C-15, C-18 | |
| **18** | 156.68 [c, j] | | | |
| **21** | 129.4 | | | |
| **22/26** | 132.5 | 6.92 (2H, d 9.0) | C-4, C-24 | H-23/25 |
| **23/25** | 115.7 | 6.62 (2H, d, 8.3) | C-21, C-24 | |
| **24** | 156.2 | | | |
| **27** | 135.2 | | | |
| **28/32** | 132.0 | 7.06 (2H, d, 8.6) | C-3, C-30 | |
| **29/31** | 121.9 | 7.12 (2H, d, 8.3) | C-27, C-30 | H-28/32 |
| **30** | 151.6 | | | |
| **34** | 132.8 [e] | | | |
| **35/39** | 129.87 [c, f] | 6.91 [g] (2H, d, 8.3) | C-14/33, C-37 | H-36/38 |
| **36/38** | 116.30 [c, h] | 6.69 [i] (2H, d, 8.3) | C-14/33, C-34, C-37 | |
| **37** | 156.70 [c, j] | | | |

[a] recorded at 150 MHz in $CD_3OD$, [b] recorded at 600 MHz in $CD_3OD$, [c] values given to 2 decimal places to separate signals, [d-j] assignment may be interchanged

**Table S5**. $^{1}$H and $^{13}$C NMR data of denigrin L (**5**)

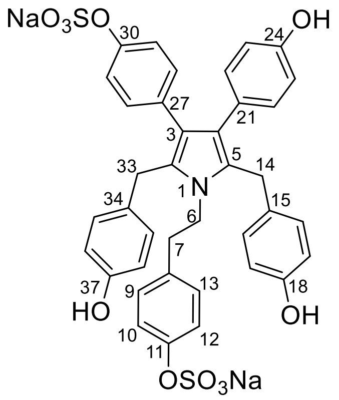

| No. | $\delta_C$ [a] | $^{1}$H $\delta_H$ (mult, *J* in Hz) [b] | HMBC ($^{1}$H to $^{13}$C) | COSY |
|---|---|---|---|---|
| **2** | 128.6 [c] | | | |
| **3** | 123.0 | | | |
| **4** | 123.5 | | | |
| **5** | 128.7 [c] | | | |
| **6** | 47.2 | 3.61 (2H, m) | C-2, C-5 | H-7 |
| **7** | 37.6 | 2.45 (2H, m) | C-9/13, C-8 | |
| **8** | 136.5 | | | |
| **9/13** | 130.4 | 6.80 (2H, d, 8.6) | C-7, C-11 | |
| **10/12** | 122.5 | 7.14 [d] (2H, d, 8.6) | C-7, C-8 | H-9/13 |
| **11** | 152.5 | | | |
| **14** | 30.7 | 3.94 (2H, s) | C-2, C-3, C-15 | |
| **15** | 132.7 [e, f] | | | |
| **16/20** | 130.00 [g, h] | 6.95 [i] (2H, d, 8.6) | C-18 | C-17-19 |
| **17/19** | 116.41 [g, j] | 6.710 [k, l] (2H, d, 8.6) | C-15, C-18 | |
| **18** | 156.83 [g, m] | | | |
| **21** | 129.3 | | | |
| **22/26** | 132.6 [e] | 6.95 [h] (2H, d, 8.6) | C-4, C-24 | C-23/25 |
| **23/25** | 115.8 | 6.63 (2H, d, 8.7) | C-21, C-24 | |
| **24** | 156.3 | | | |
| **27** | 135.1 [f] | | | |
| **28/32** | 132.0 | 7.09 (2H, d, 8.8) | C-3, C-30 | |
| **29/31** | 121.9 | 7.14 [d] (2H, d, 8.6) | C-30 | H-28/32 |
| **30** | 151.7 | | | |
| **33** | 30.7 | 3.87 (2H, s) | C-4, C-5, C-34 | |
| **34** | 132.6 [e, f] | | | |
| **35/39** | 129.96 [g, h] | 6.95 [i] (2H, d, 8.6) | C-37 | C-36/38 |
| **36/38** | 116.37 [g, j] | 6.708 [k, l] (2H, d, 8.6) | C-34, C37 | |
| **37** | 156.78 [g, m] | | | |

[a] recorded at 150 MHz in $CD_3OD$, [b] recorded at 600 MHz in $CD_3OD$, [c-e, h-j, l-m] assignment may be interchanged, [f] assigned based on denigrin J (**3**) and K (**4**), [g] values given to 2 decimal places to separate signals, [k] values given to 3 decimal places to separate signals

**Figure S1a.** $^{1}$H NMR (methanol-d4, 600 MHz) spectrum of denigrin H (**1**)

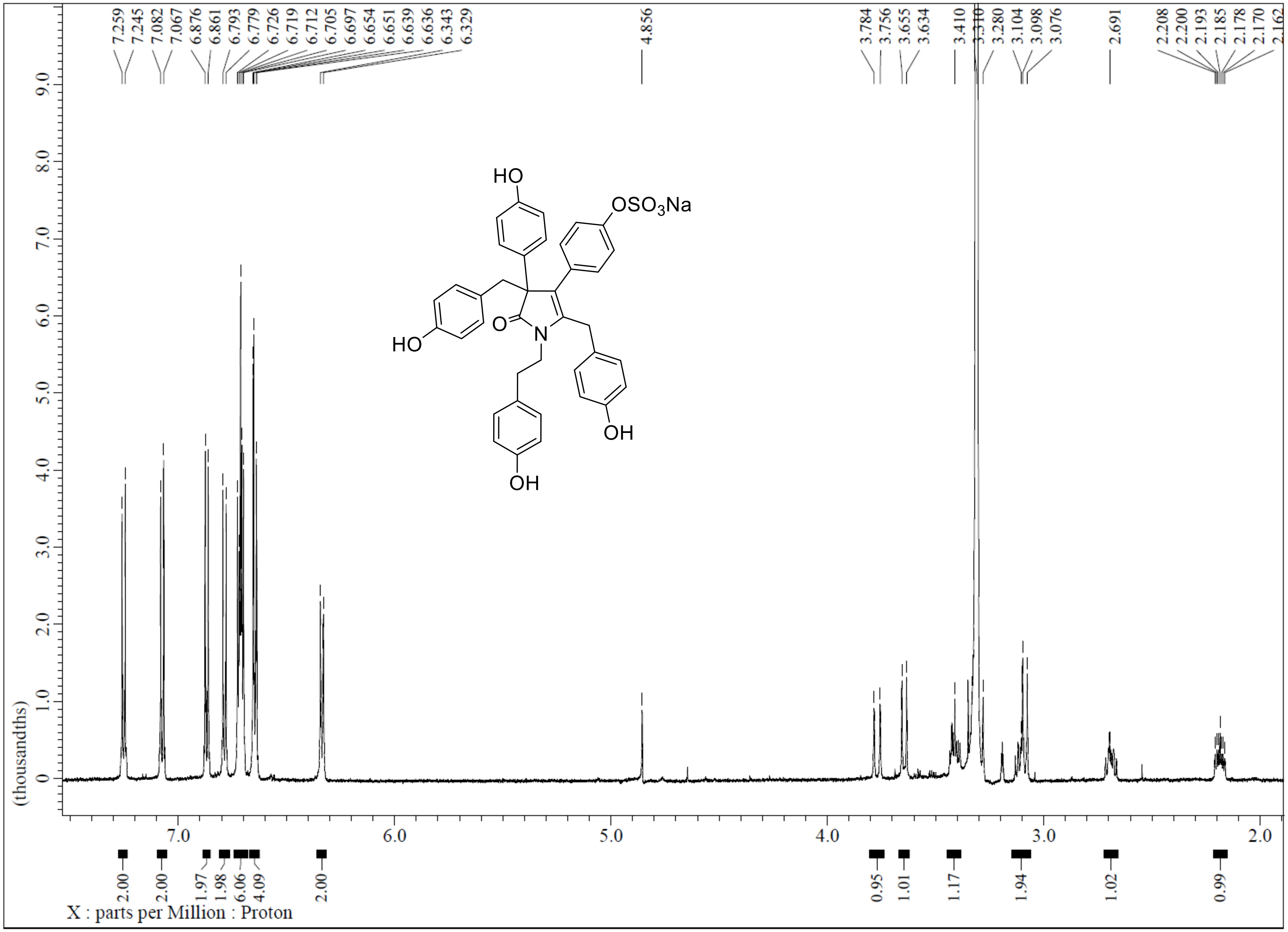

**Figure S1b.** $^{13}$C NMR (methanol-d4, 150 MHz) spectrum of denigrin H (**1**)

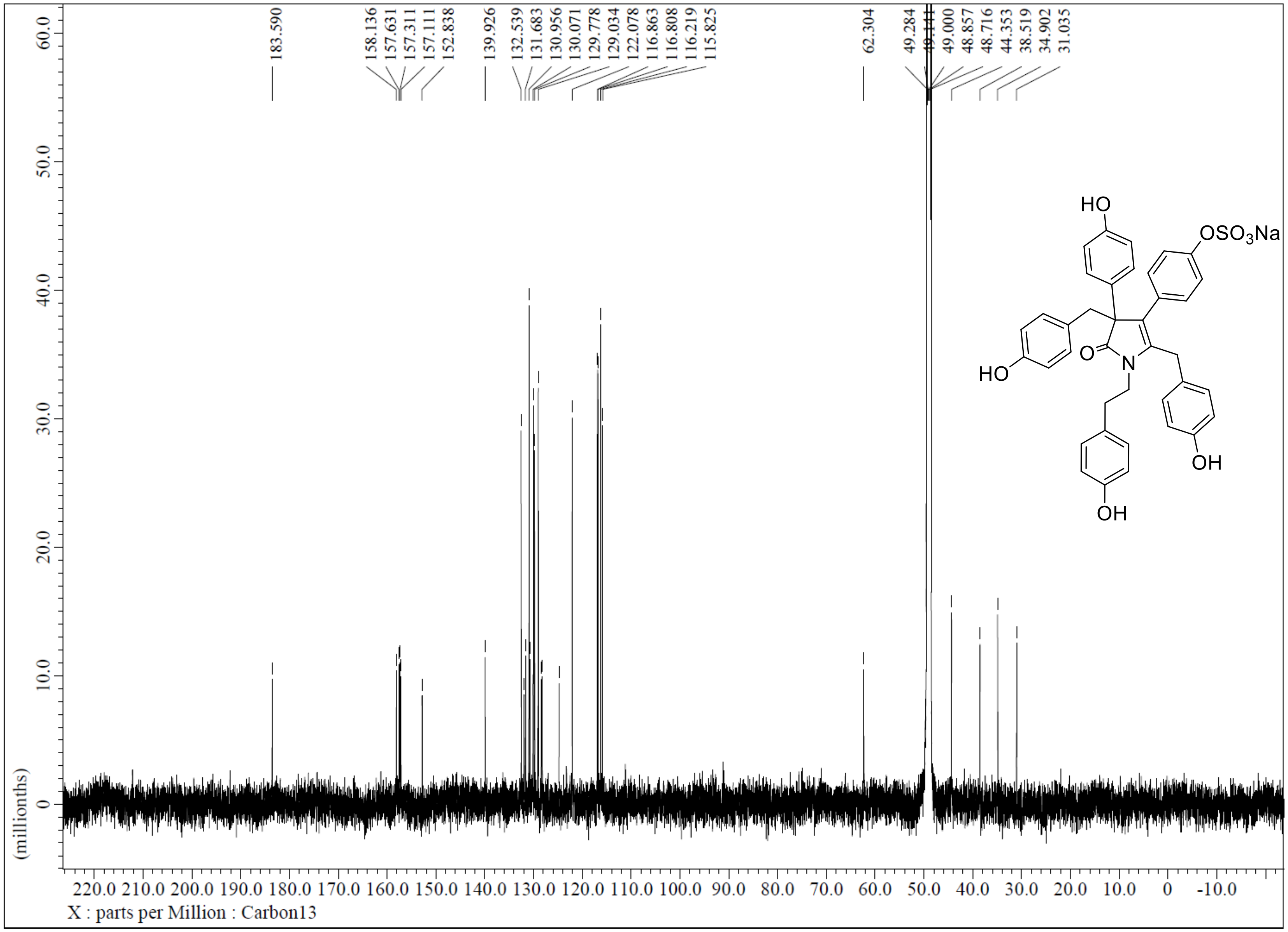

**Figure S1c.** $^{13}$C NMR (methanol-d4, 150 MHz) spectrum expansion 1 of denigrin H (**1**)

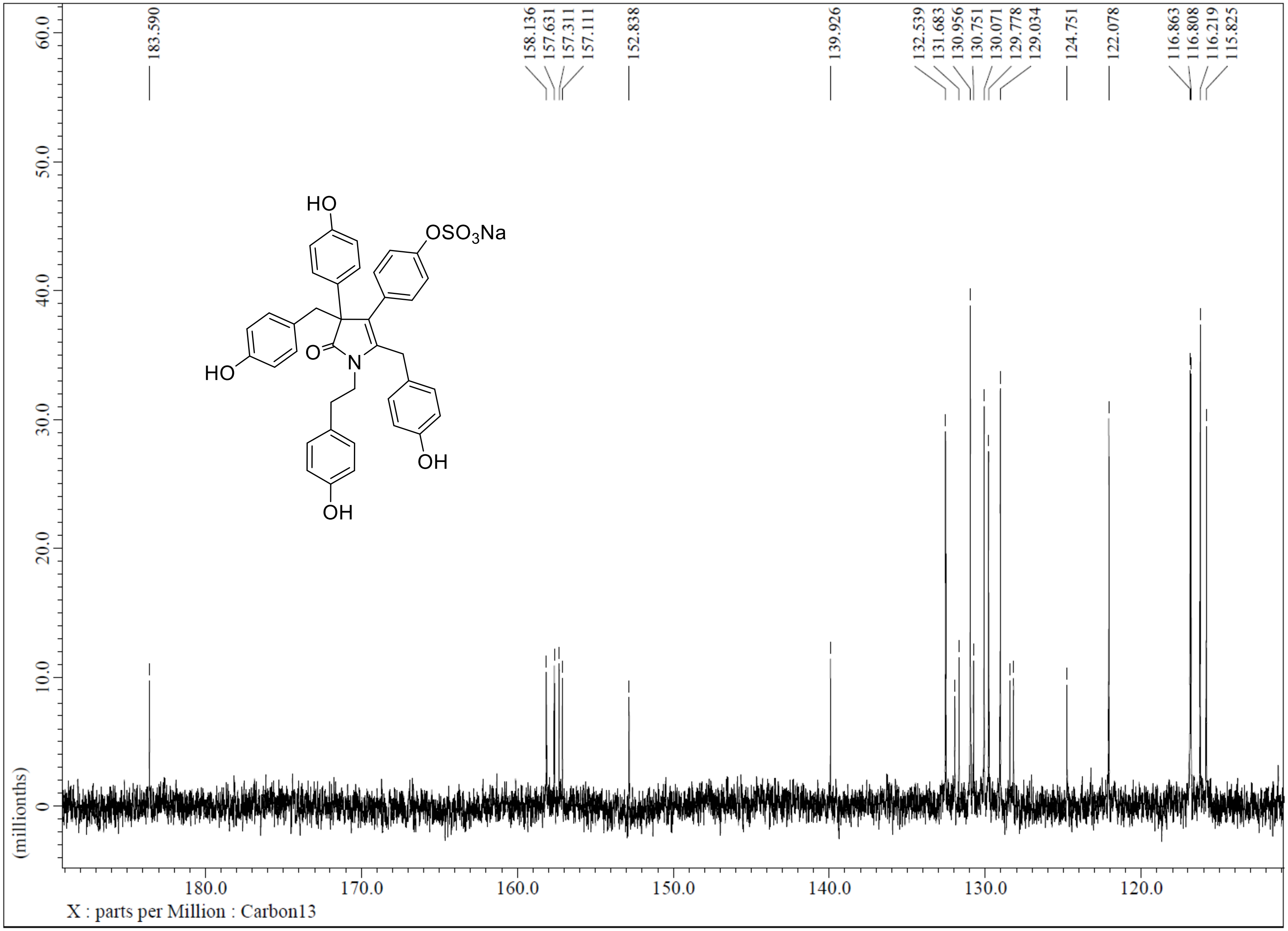

**Figure S1d.** $^{13}$C NMR (methanol-*d4*, 150 MHz) spectrum expansion 2 of denigrin H (**1**)

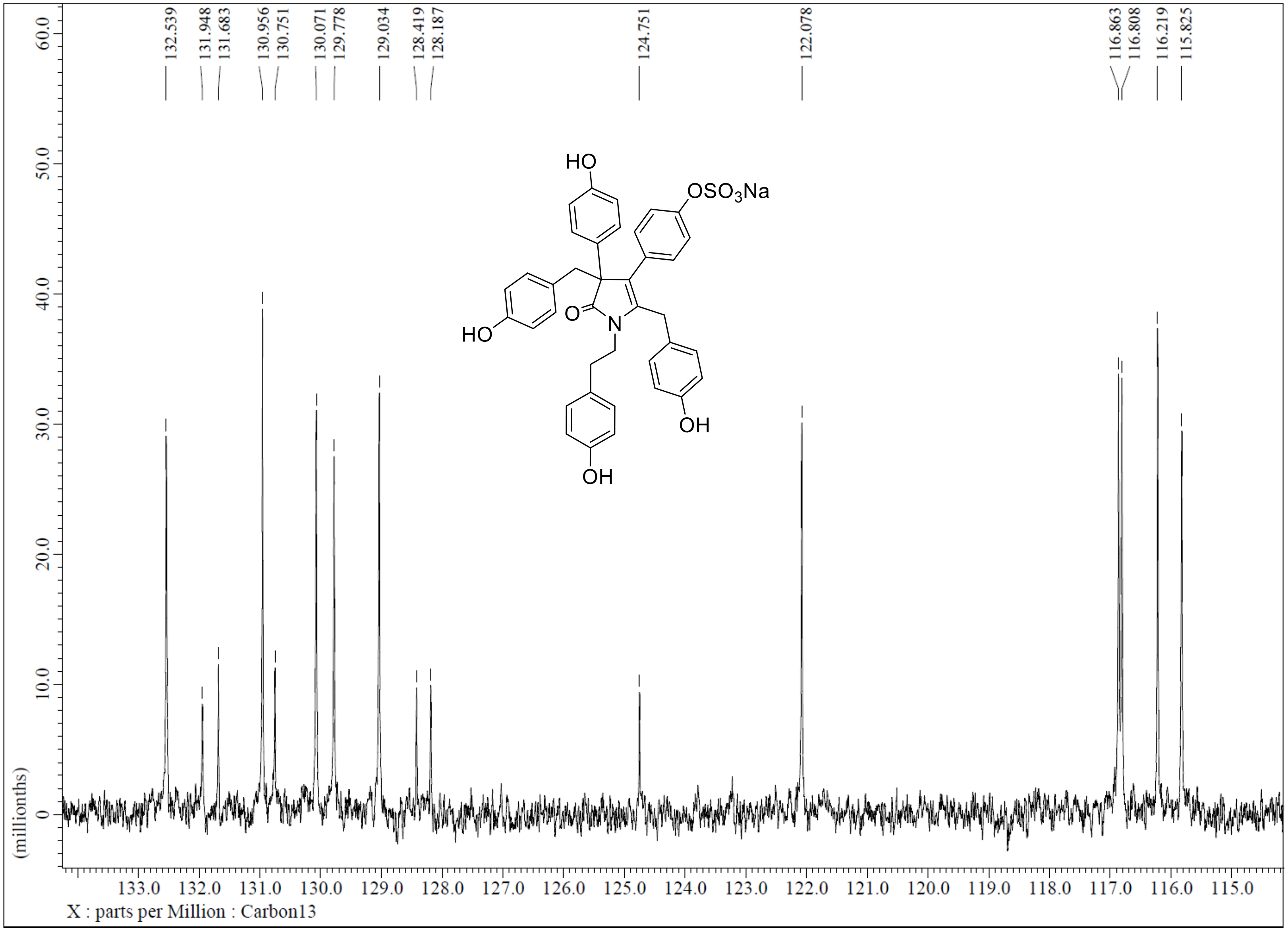

**Figure S1e.** COSY (methanol-*d4*) spectrum of denigrin H (**1**)

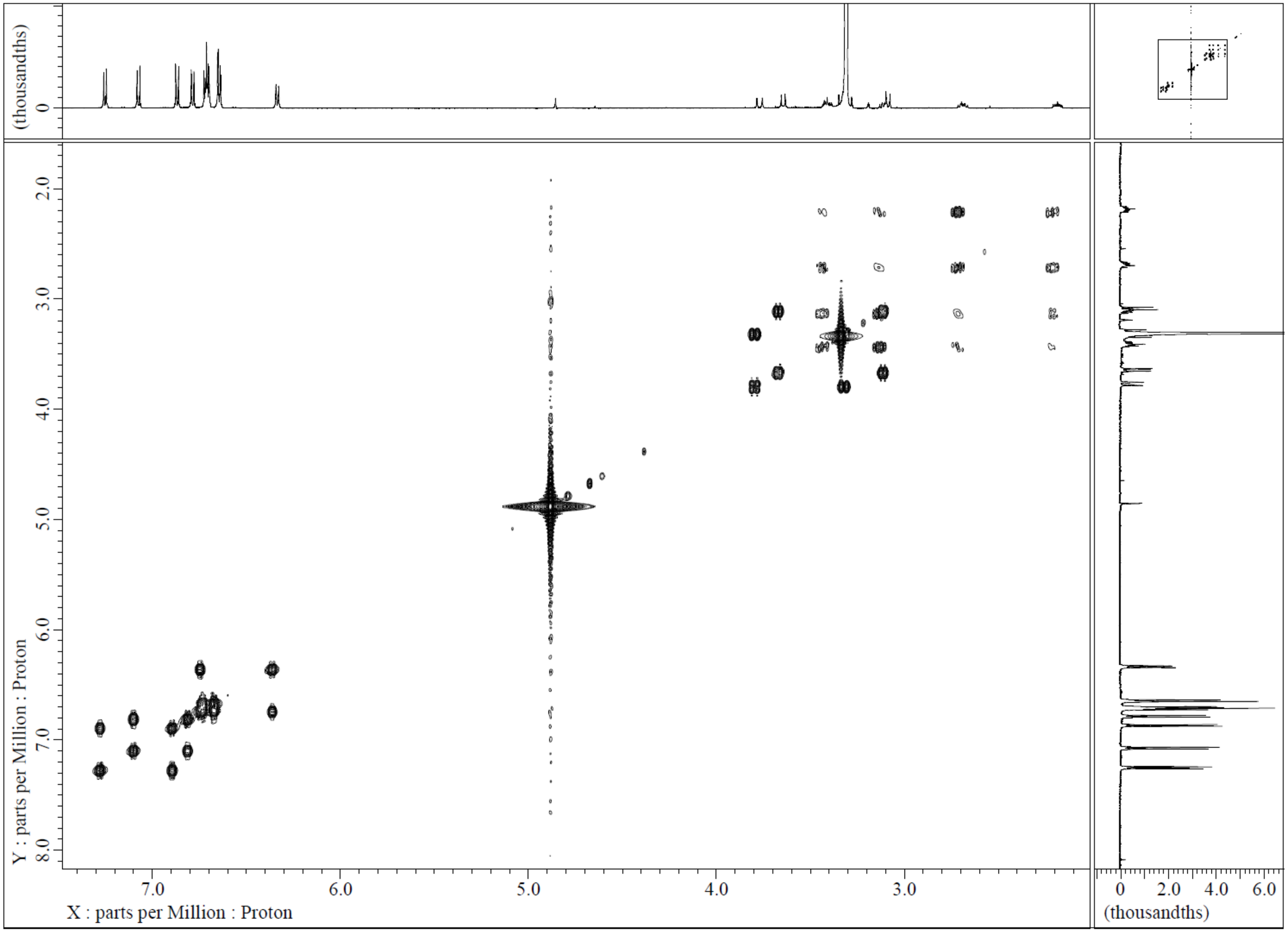

**Figure S1f.** COSY (methanol-*d4*) spectrum of denigrin H (**1**)

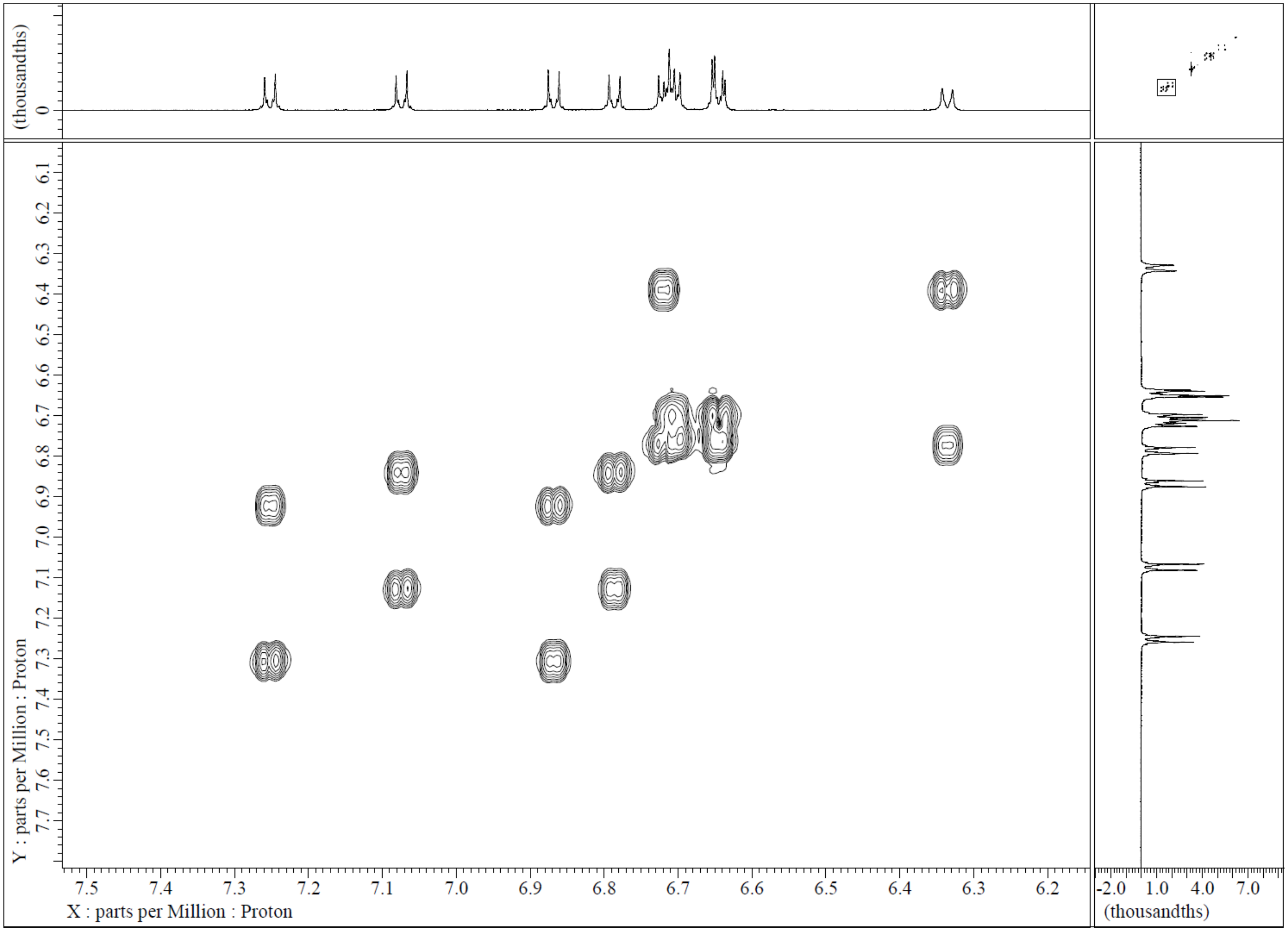

**Figure S1g.** gHSQC (methanol-*d4*) spectrum of denigrin H (**1**)

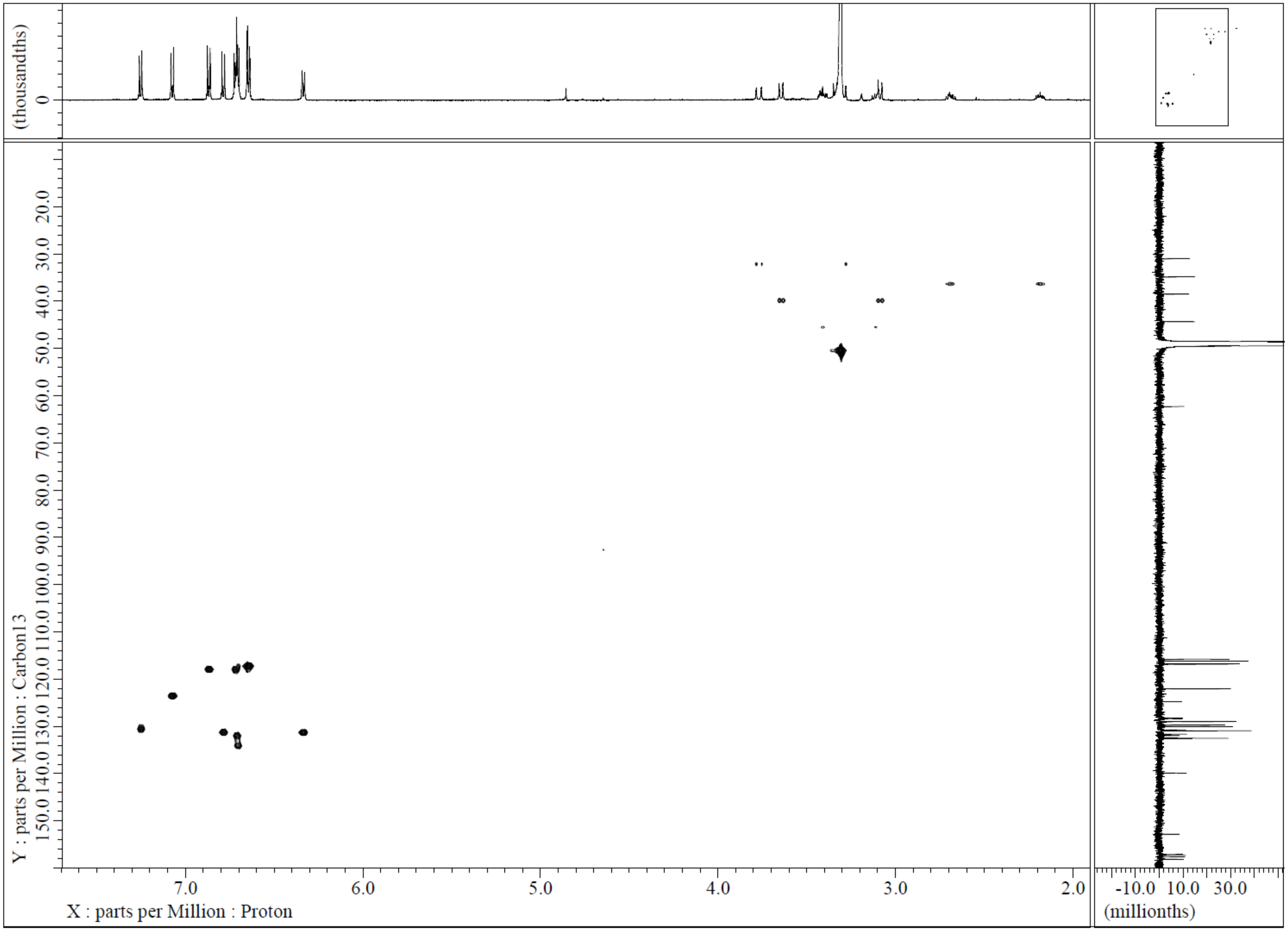

**Figure S1h.** gHMBC (methanol-*d4*) spectrum of denigrin H (**1**)

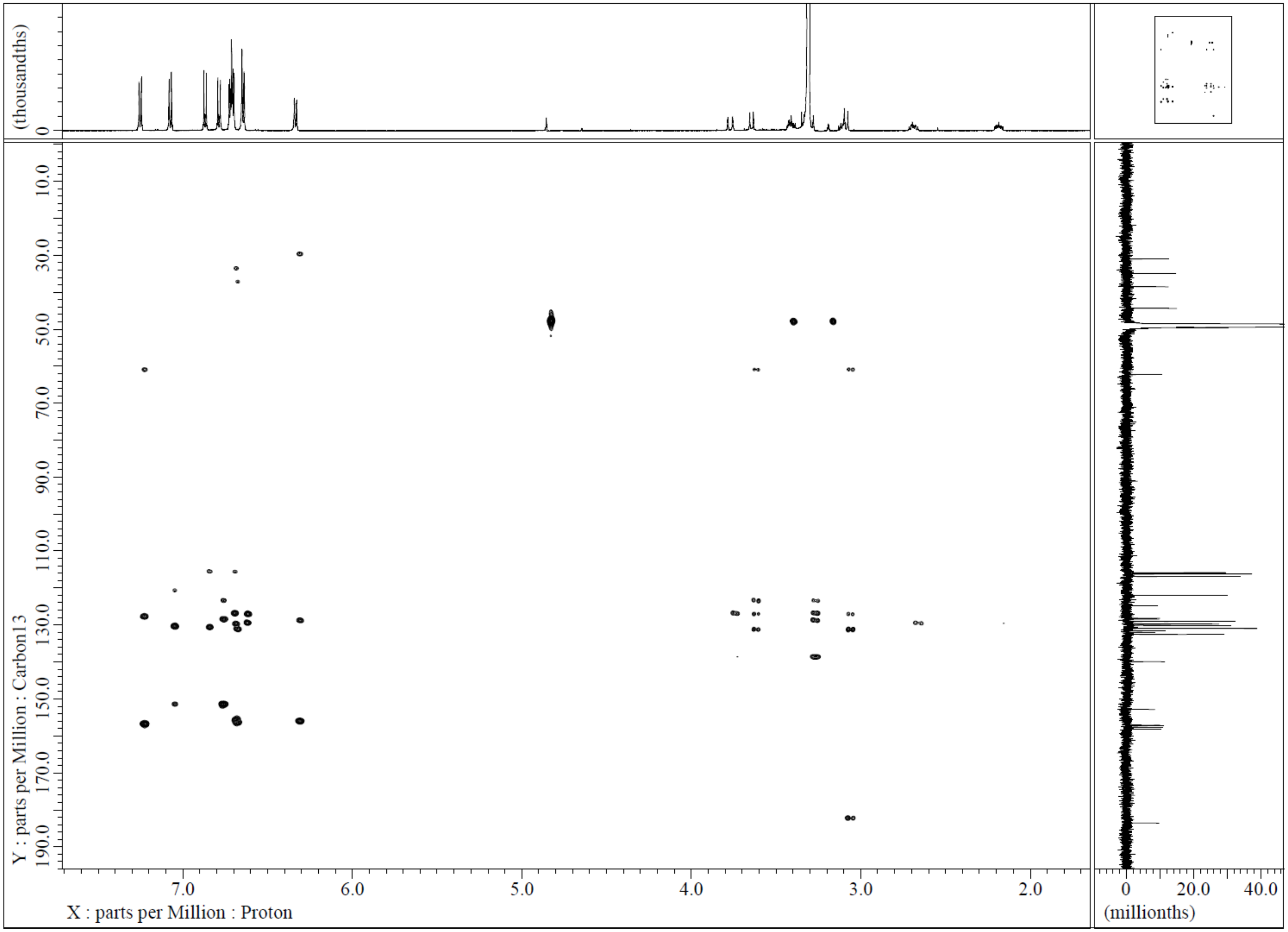

**Figure S1i.** gHMBC (methanol-*d4*) spectrum expansion of denigrin H (**1**)

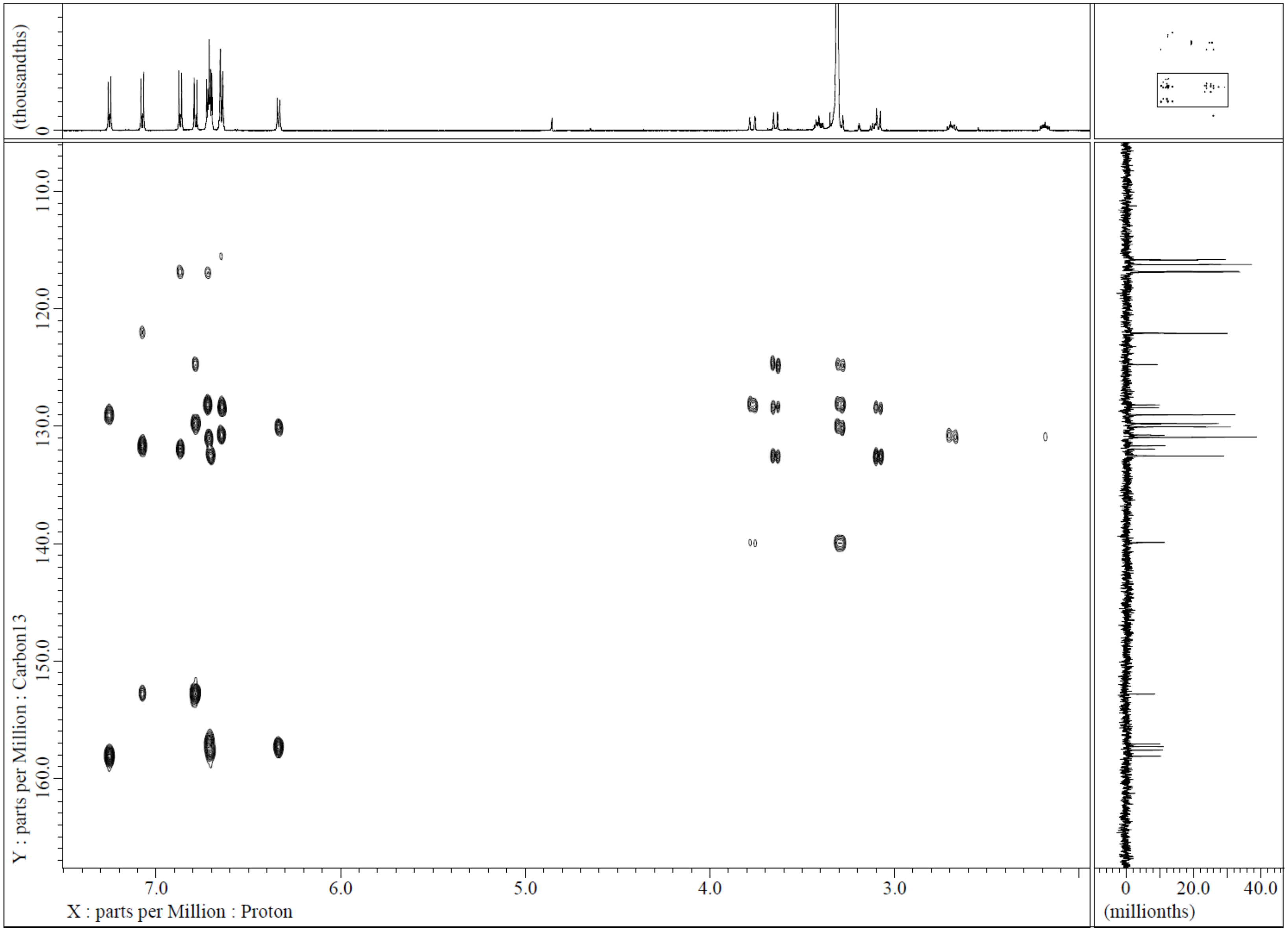

**Figure S1j**. HRESIMS spectrum of denigrin H (**1**) in negative ion mode

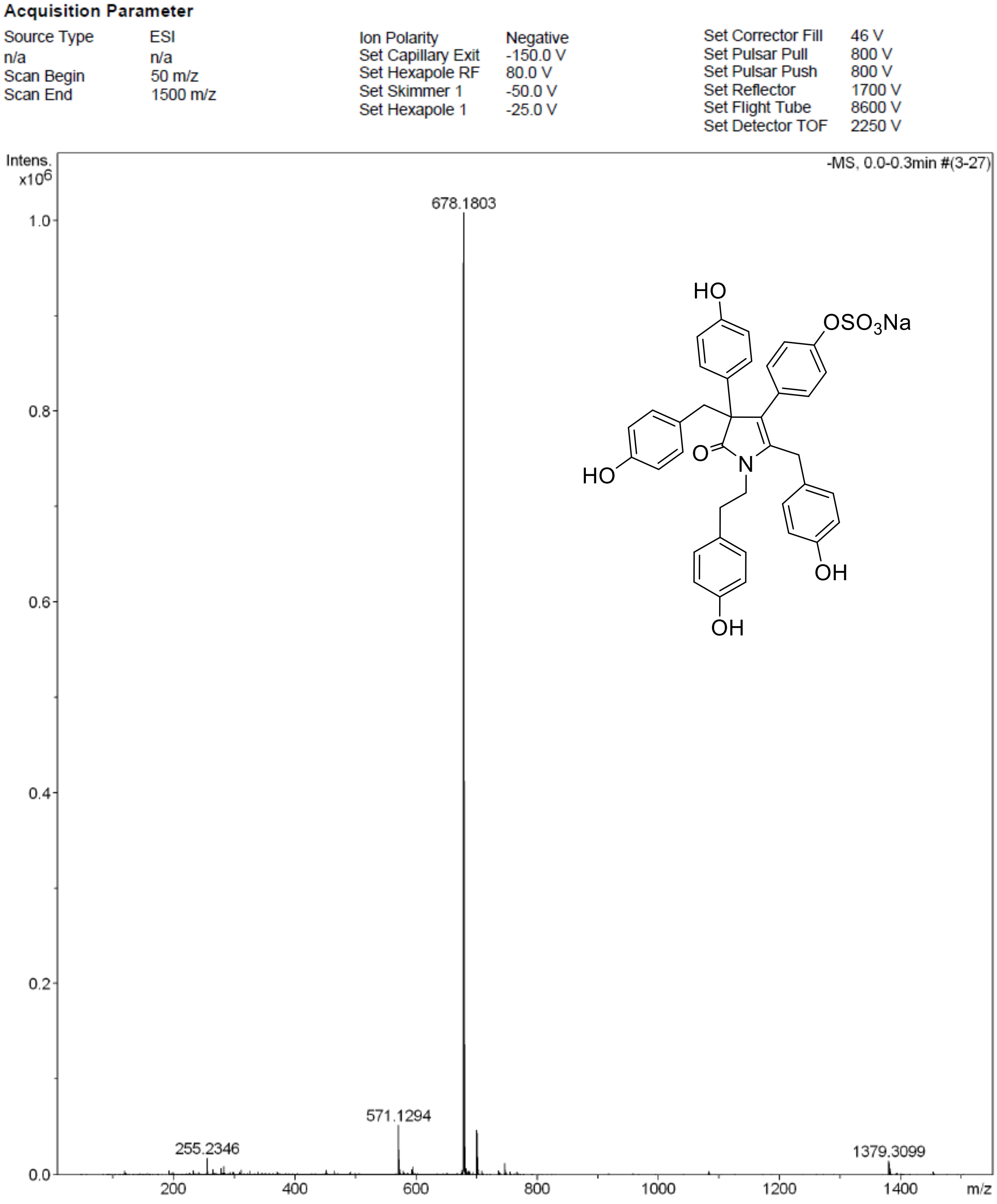

**Figure S2a.** $^{1}$H NMR (methanol-*d4*, 600 MHz) spectrum of denigrin I (**2**)

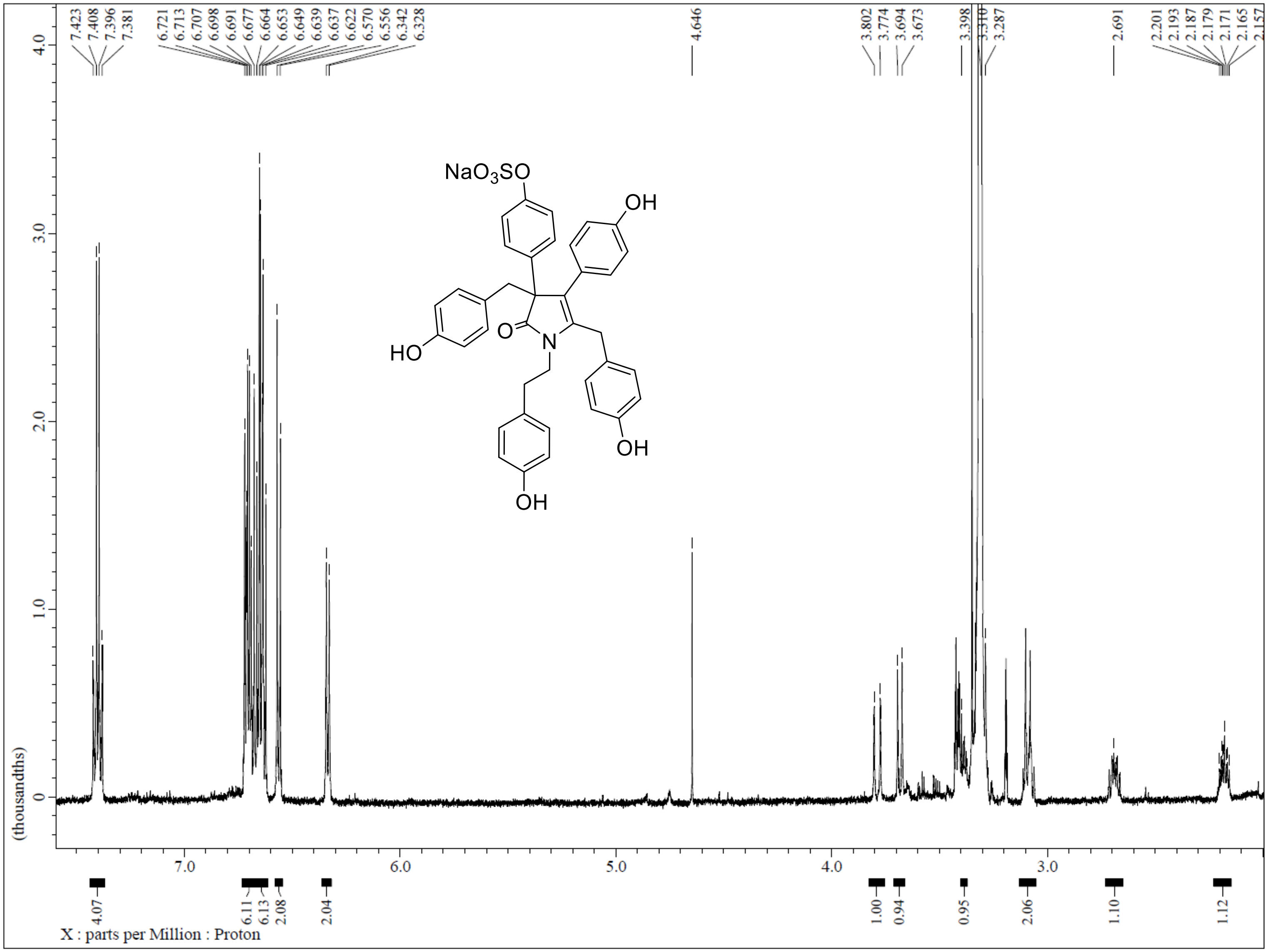

**Figure S2b.** $^{13}$C NMR (methanol-*d4*, 150 MHz) spectrum of denigrin I (**2**)

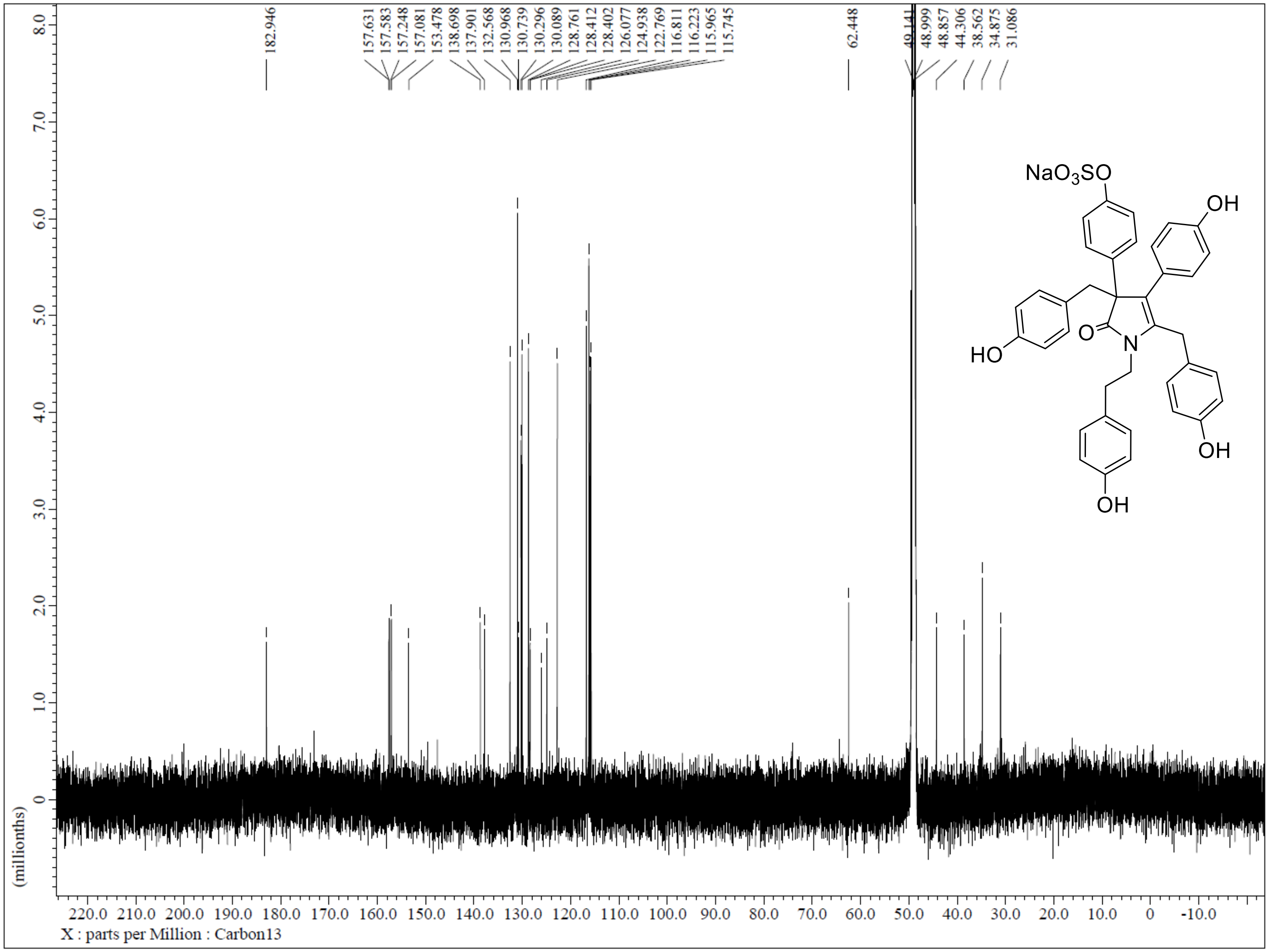

**Figure S2c.** $^{13}$C NMR (methanol-*d4*, 150 MHz) spectrum expansion of denigrin I (**2**)

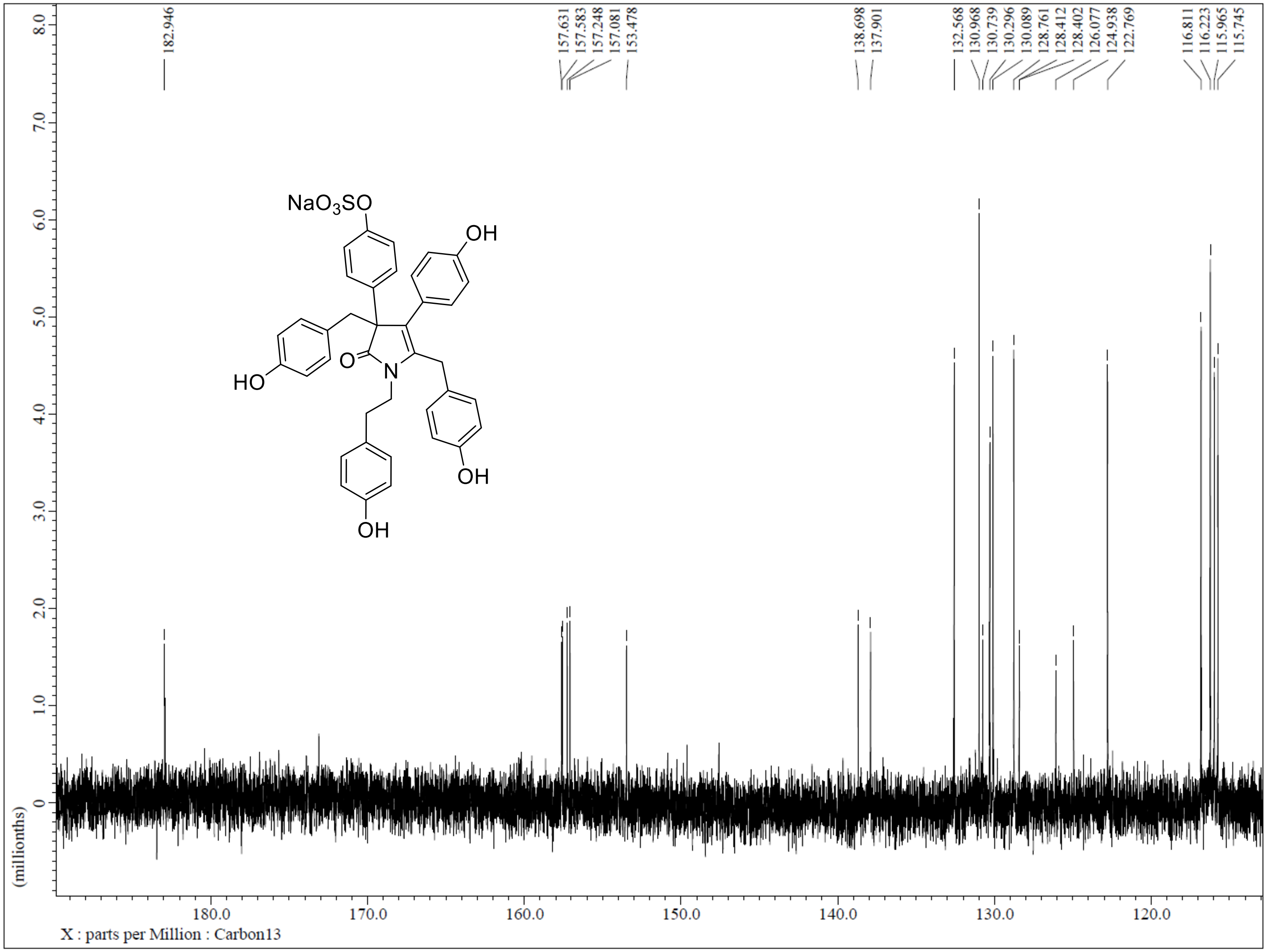

**Figure S2d.** COSY (methanol-*d4*) spectrum of denigrin I (**2**)

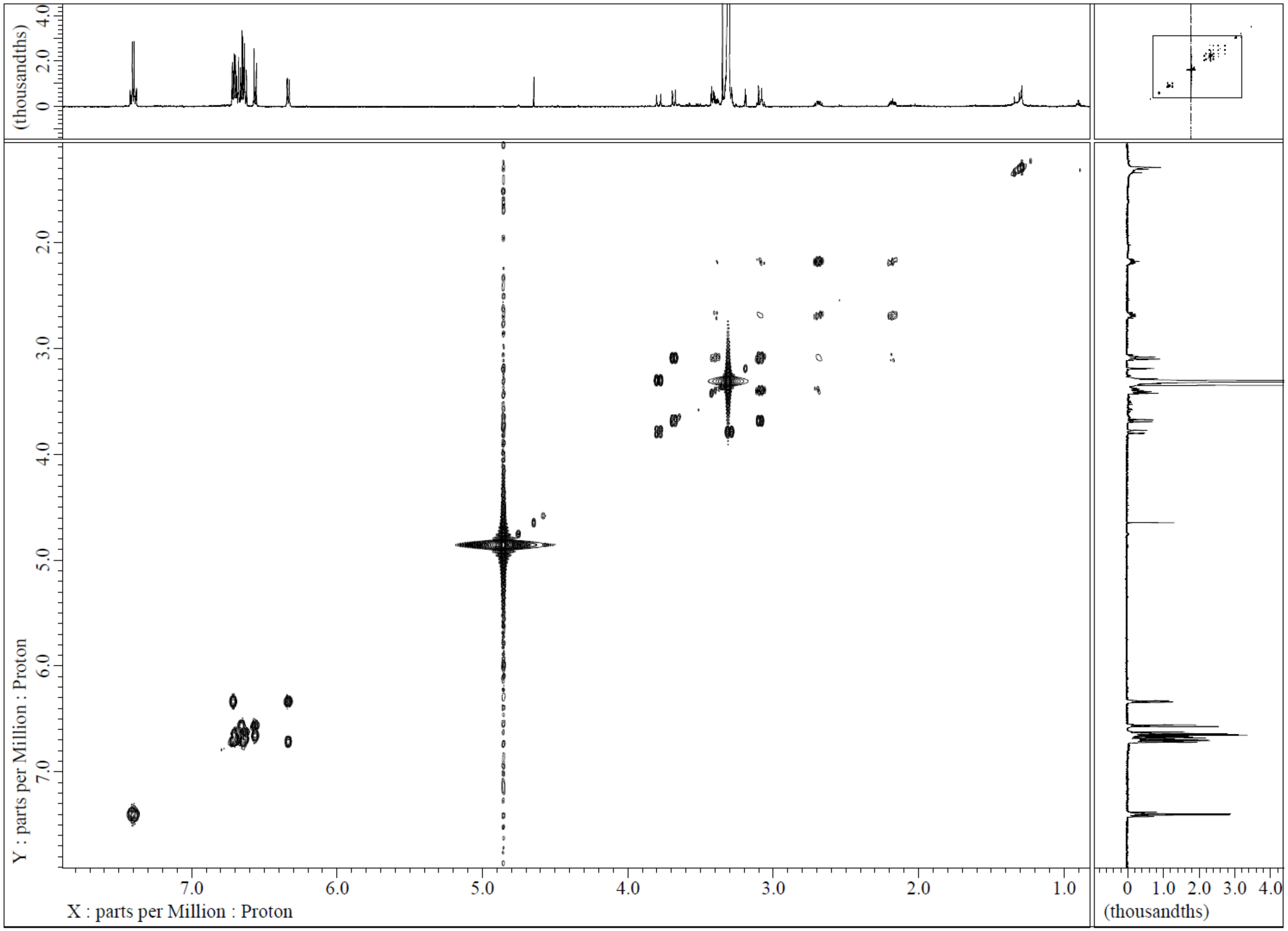

**Figure S2e**. gHSQC (methanol-d4) spectrum of denigrin I (**2**)

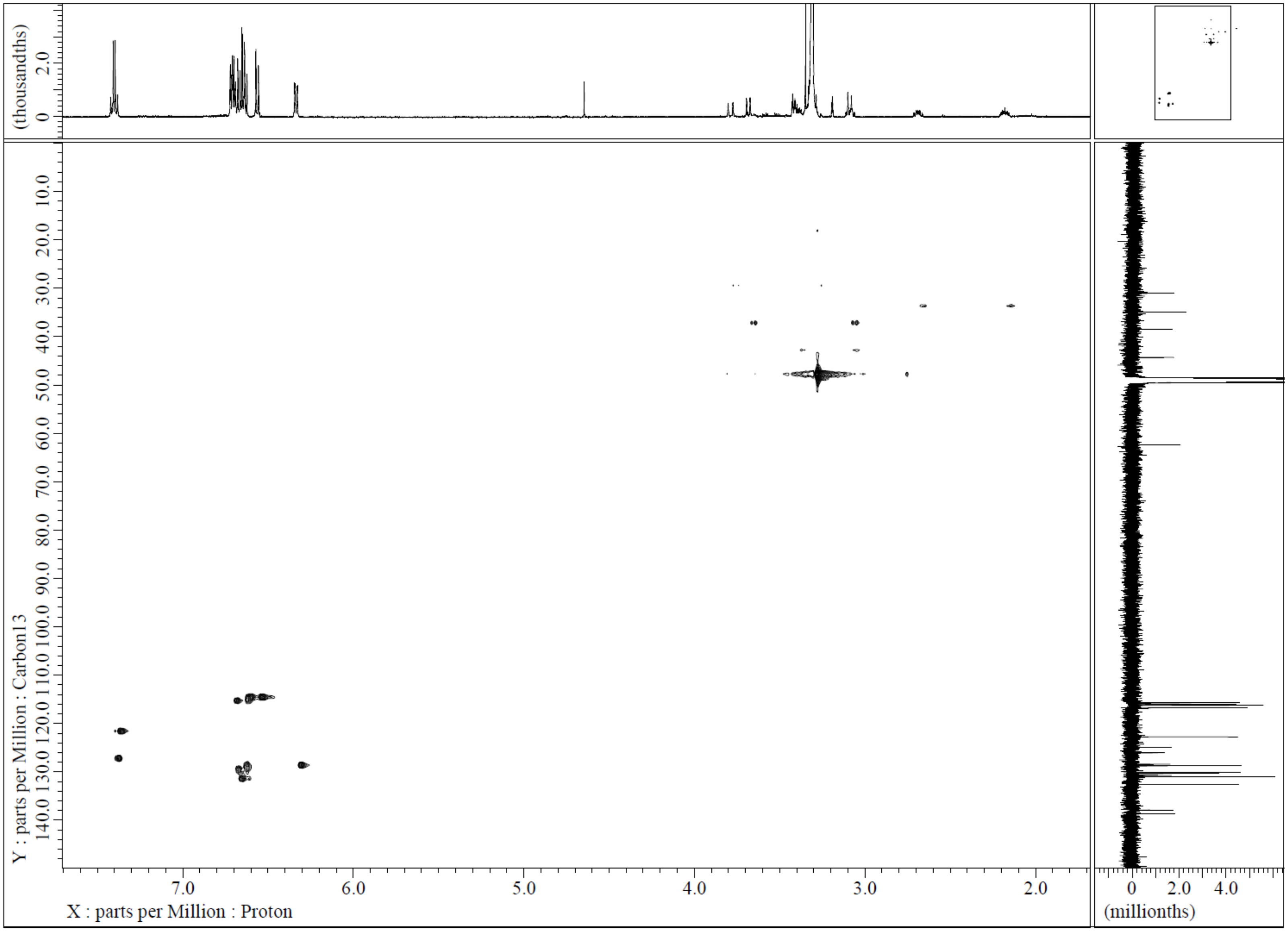

**Figure S2f.** gHMBC (methanol-*d4*) spectrum of denigrin I (**2**)

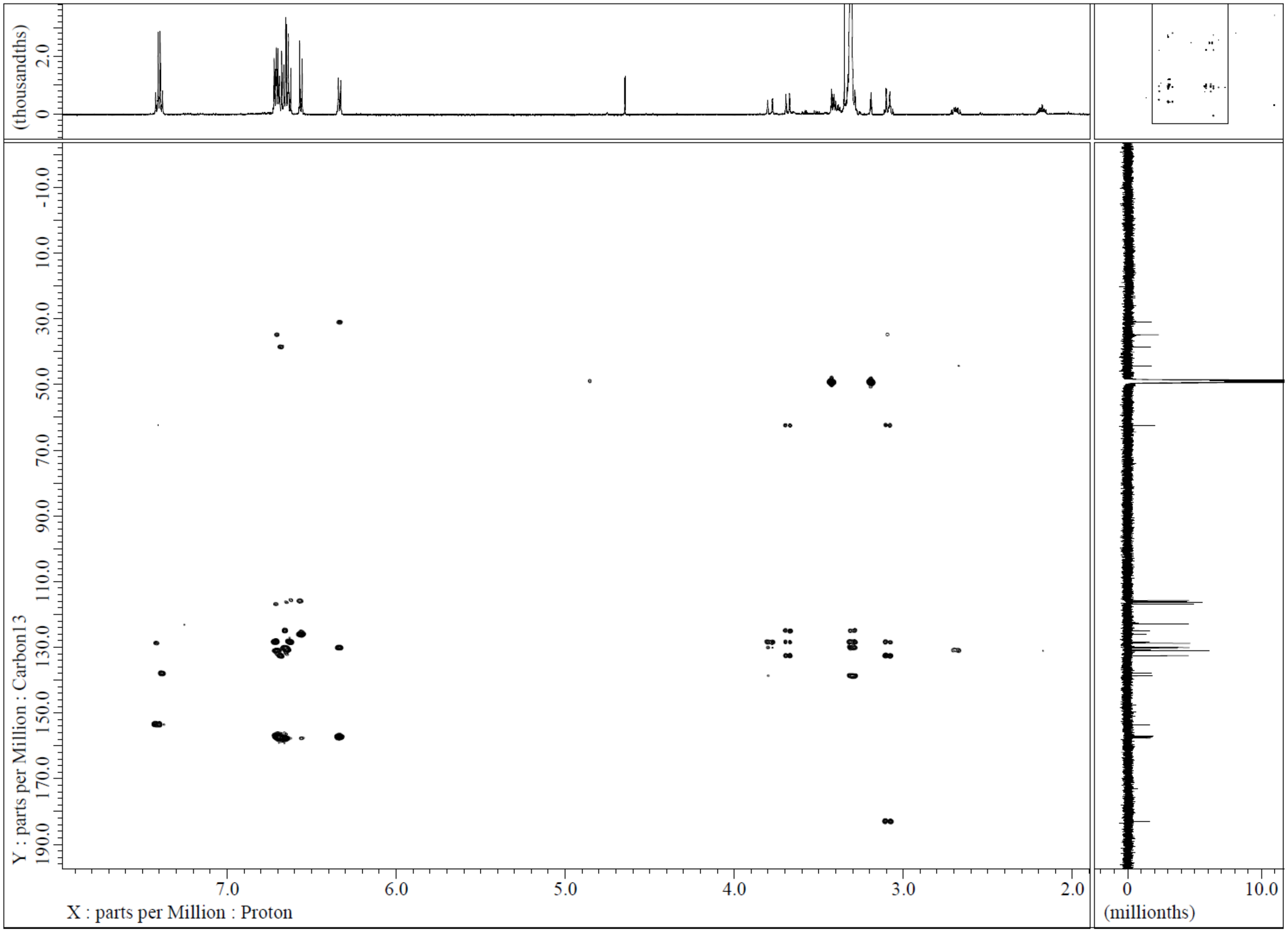

**Figure S2g.** gHMBC (methanol-*d4*) spectrum expansion of denigrin I (**2**)

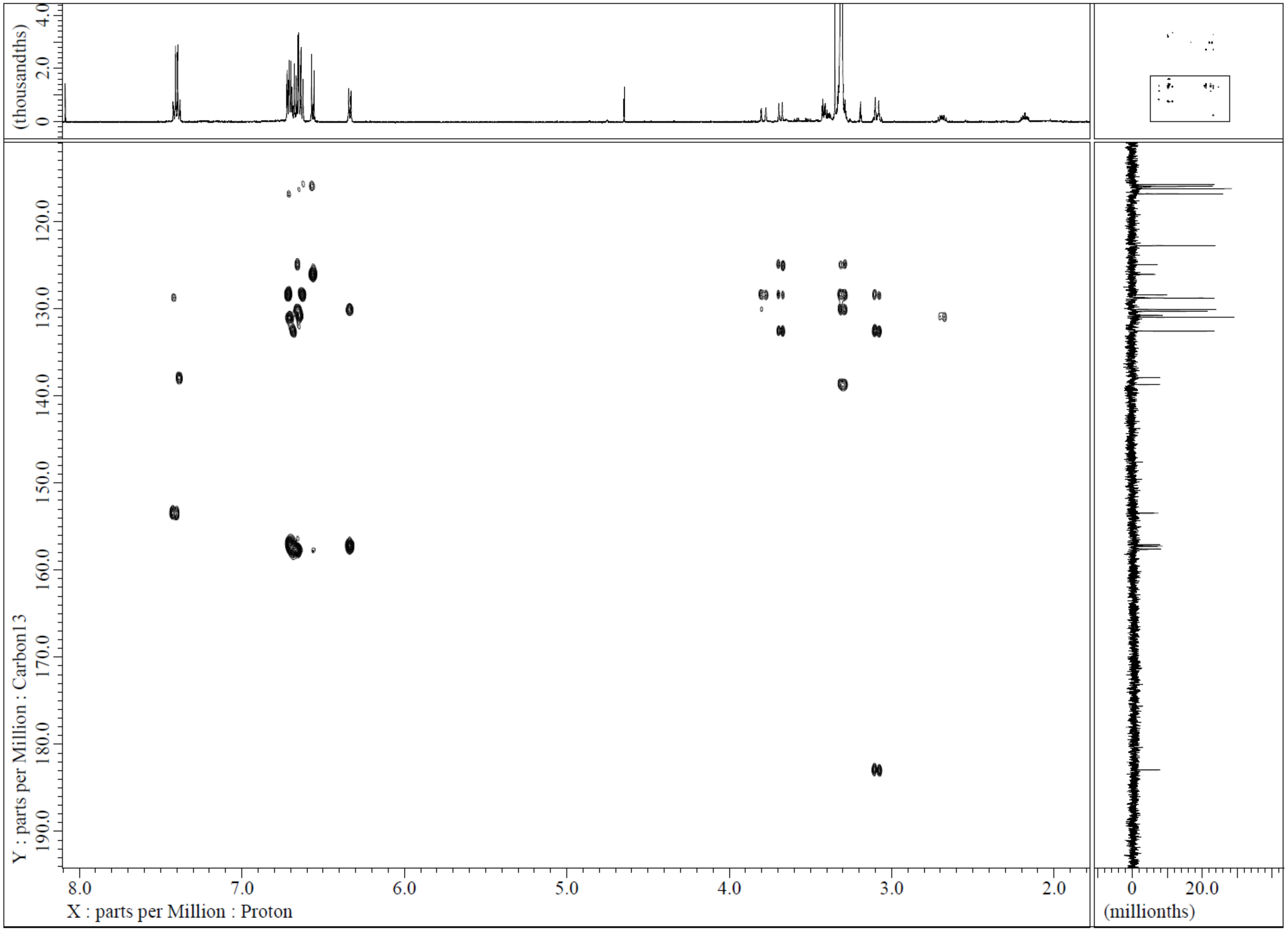

**Figure S2h**. HRESIMS spectrum of denigrin I (**2**) in negative ion mode

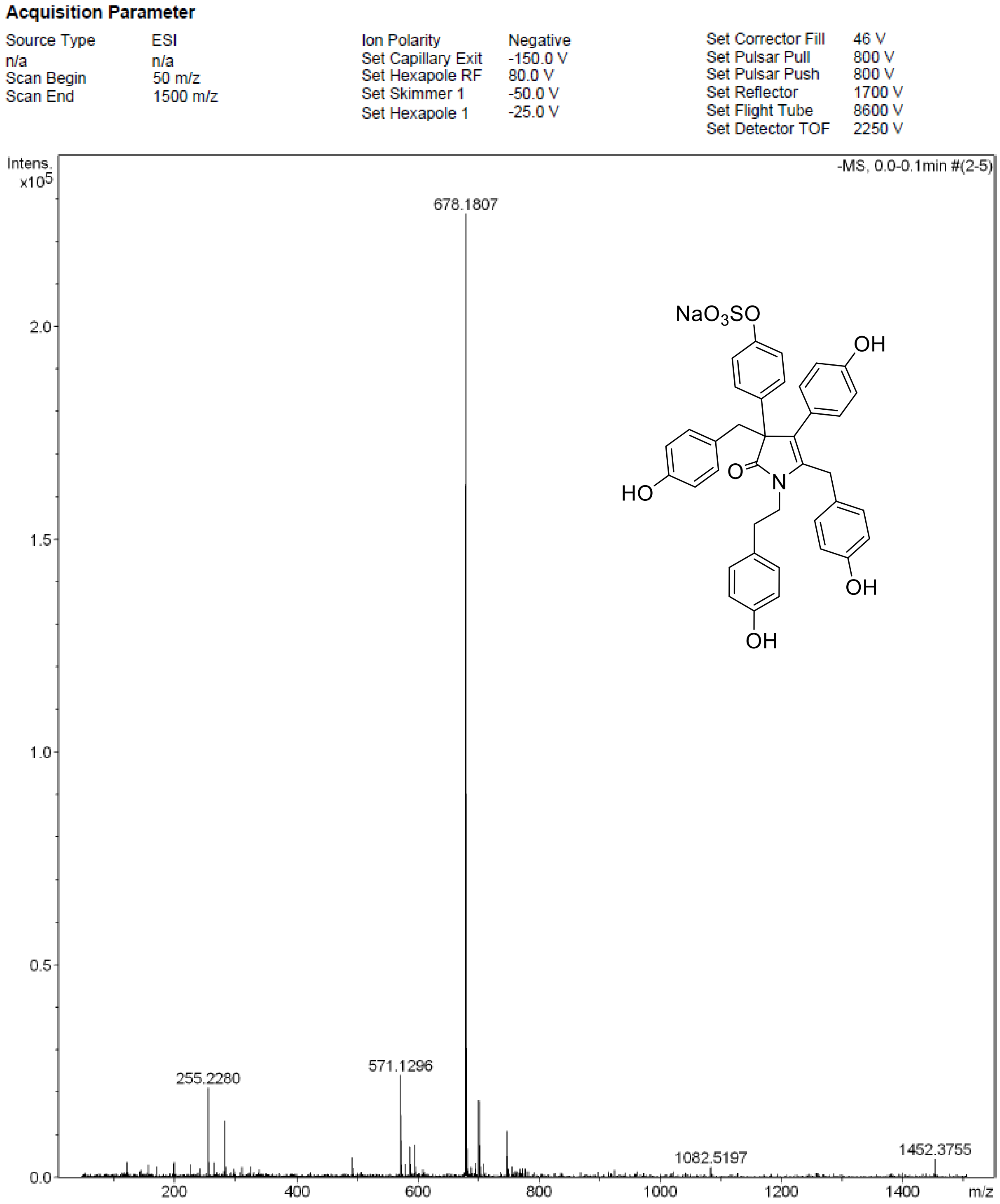

**Figure S3a.** $^{1}$H NMR (methanol-*d4*, 600 MHz) spectrum of denigrin J (**3**)

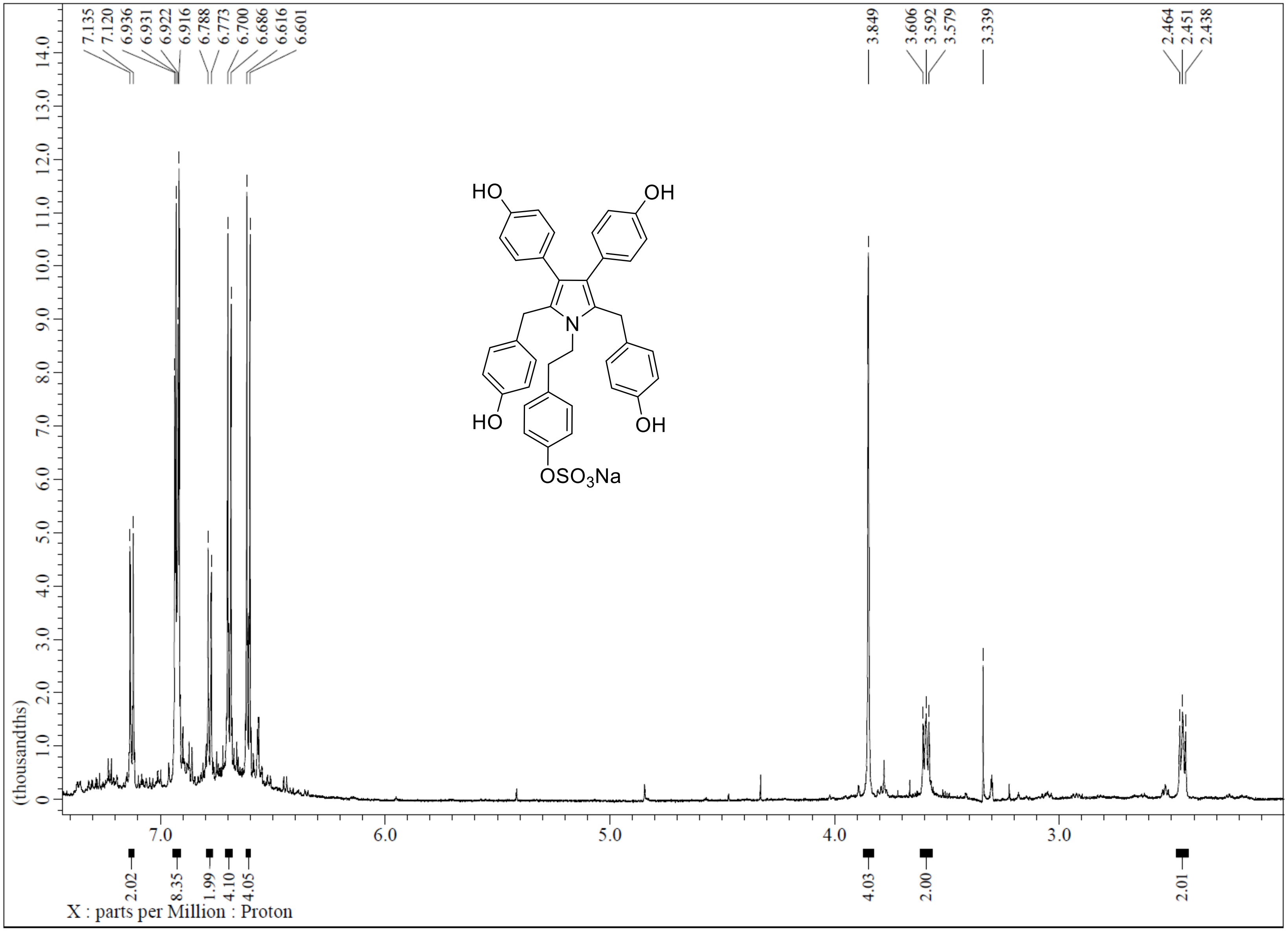

**Figure S3b.** $^{13}$C NMR (methanol-*d4*, 150 MHz) spectrum of denigrin J (**3**)

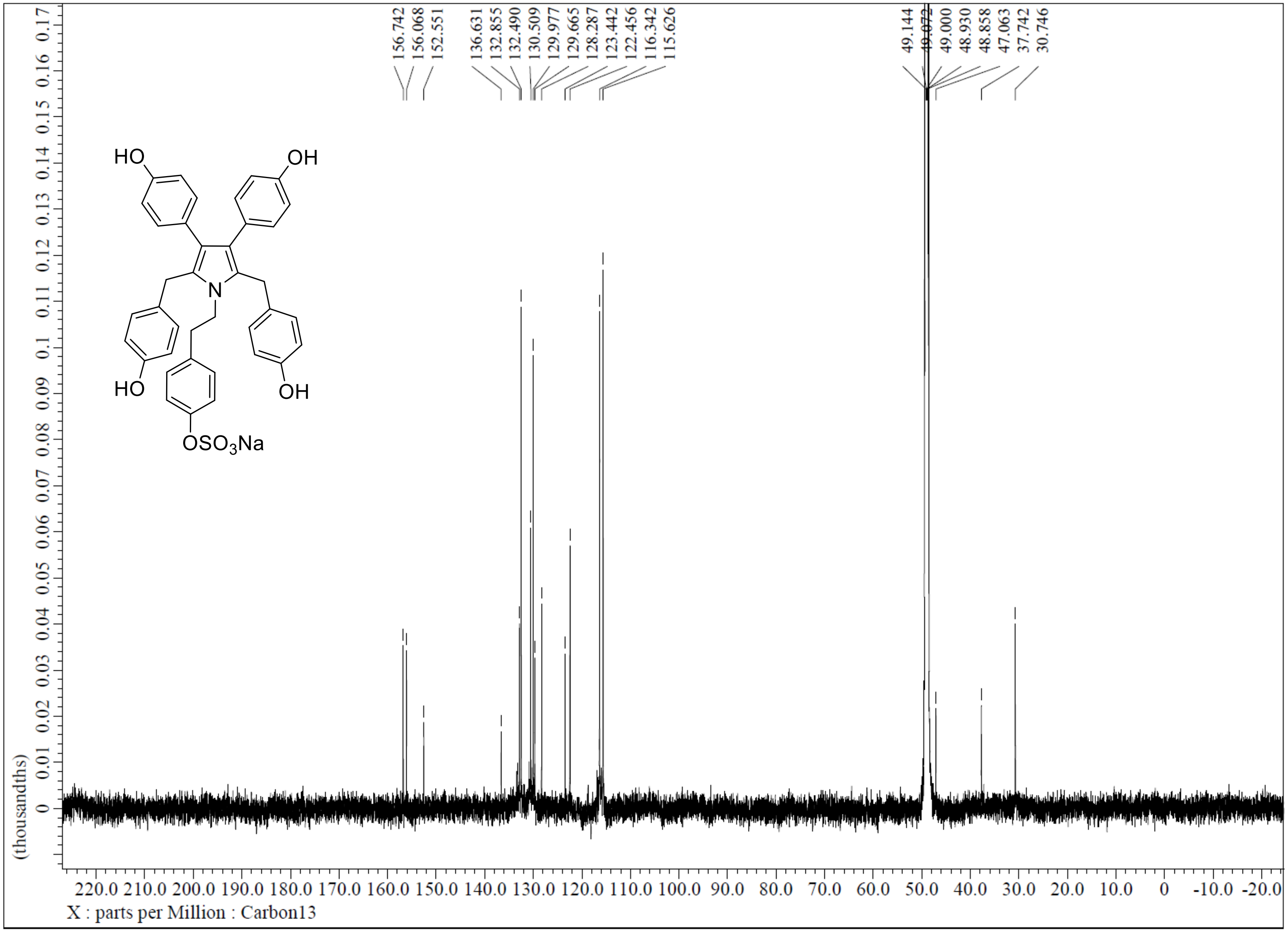

**Figure S3c.** $^{13}$C NMR (methanol-*d4*, 150 MHz) spectrum expansion of denigrin J (**3**)

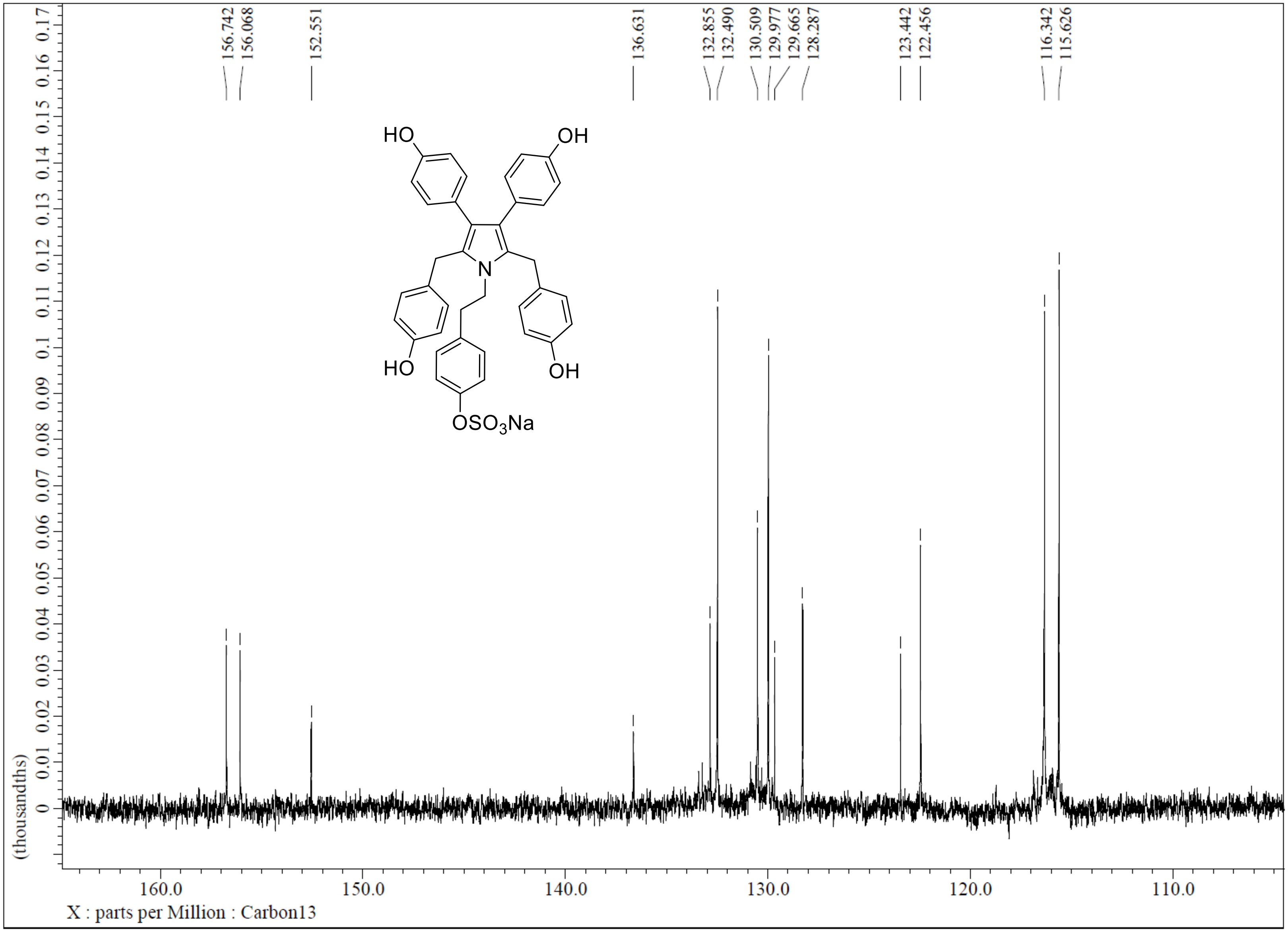

**Figure S3d.** COSY (methanol-*d4*) spectrum of denigrin J (**3**)

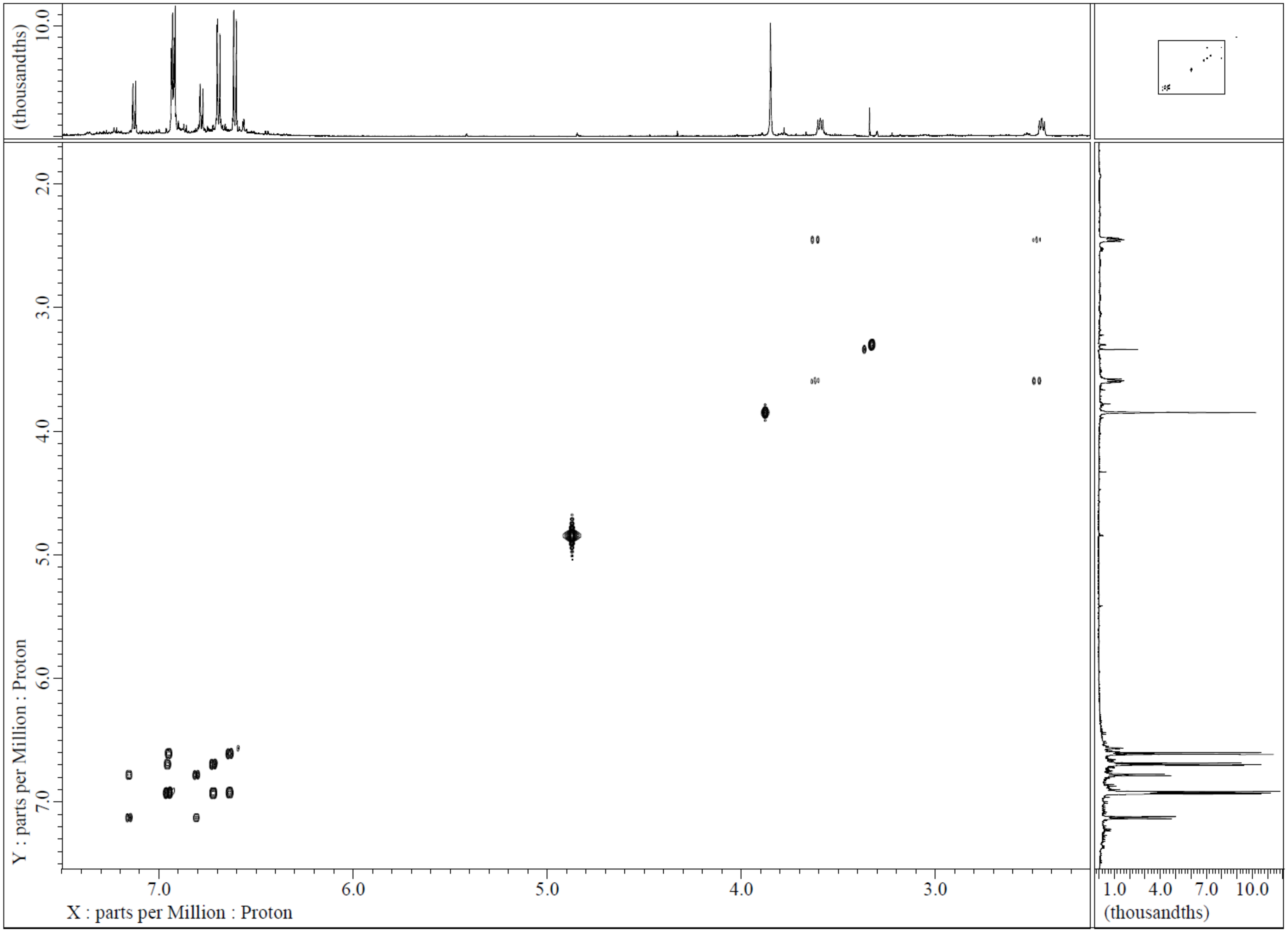

**Figure S3e.** gHSQC (methanol-*d4*) spectrum of denigrin J (**3**)

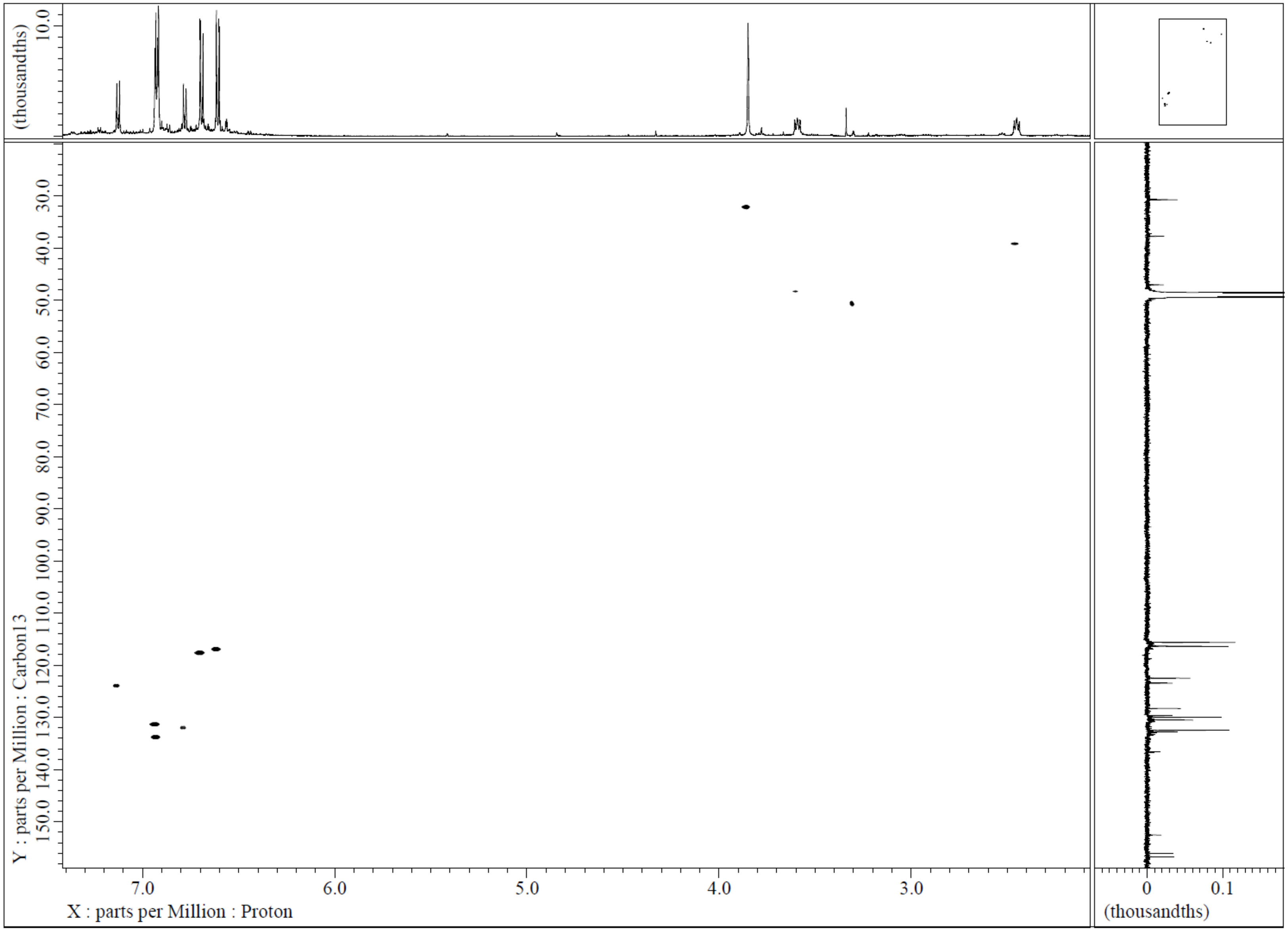

**Figure S3f.** gHMBC (methanol-*d4*) spectrum of denigrin J (**3**)

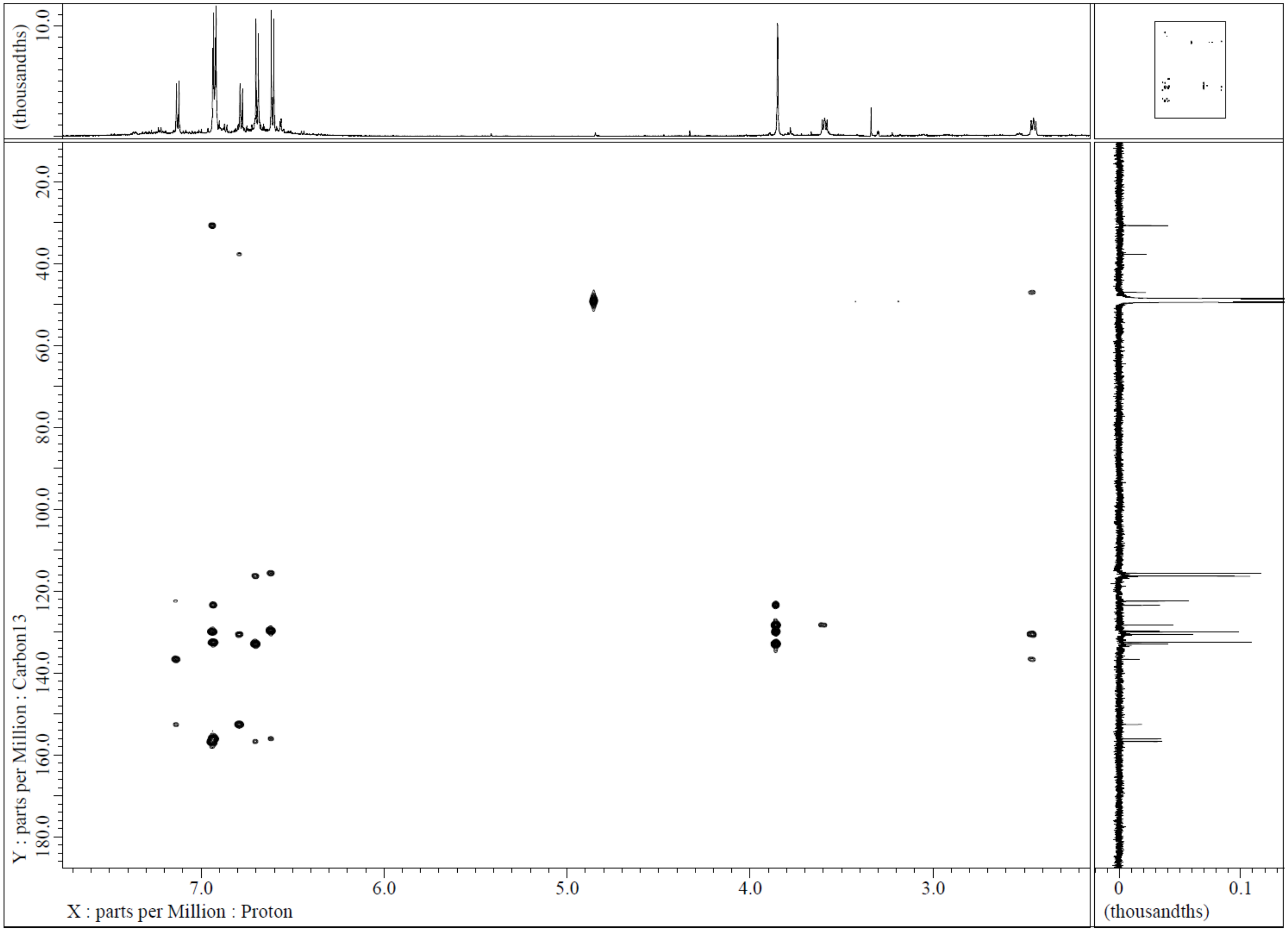

**Figure S3g.** gHMBC (methanol-*d4*) spectrum expansion of denigrin J (**3**)

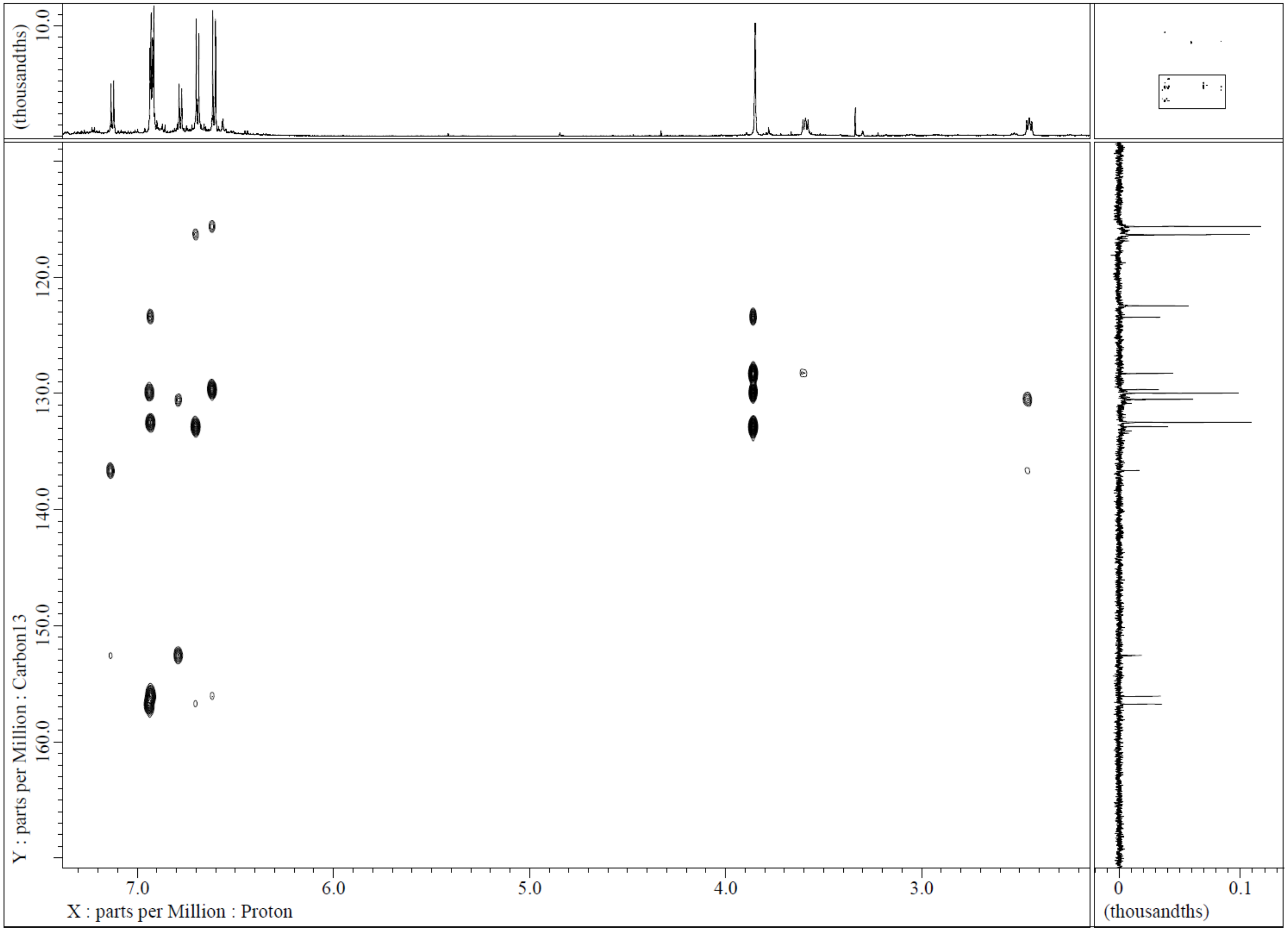

**Figure S3h**. HRESIMS spectrum of denigrin J (**3**) in negative ion mode

**Acquisition Parameter**

| | | | | | |
|---|---|---|---|---|---|
| Source Type | ESI | Ion Polarity | Negative | Set Corrector Fill | 46 V |
| n/a | n/a | Set Capillary Exit | -150.0 V | Set Pulsar Pull | 800 V |
| Scan Begin | 50 m/z | Set Hexapole RF | 80.0 V | Set Pulsar Push | 800 V |
| Scan End | 1500 m/z | Set Skimmer 1 | -50.0 V | Set Reflector | 1700 V |
| | | Set Hexapole 1 | -25.0 V | Set Flight Tube | 8600 V |
| | | | | Set Detector TOF | 2250 V |

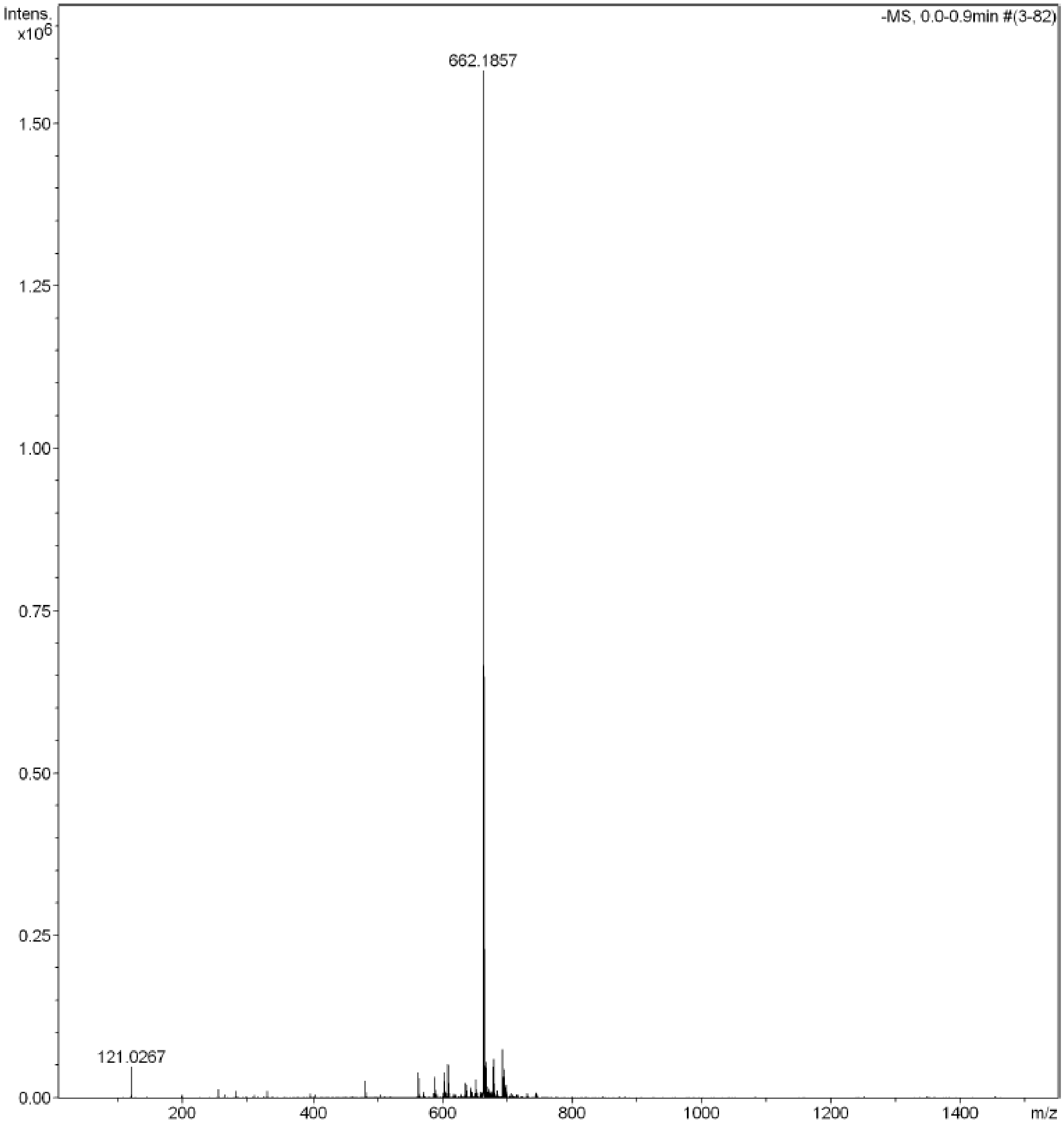

**Figure S4a.** $^{1}H$ NMR (methanol-*d4*, 600 MHz) spectrum of denigrin K (**4**)

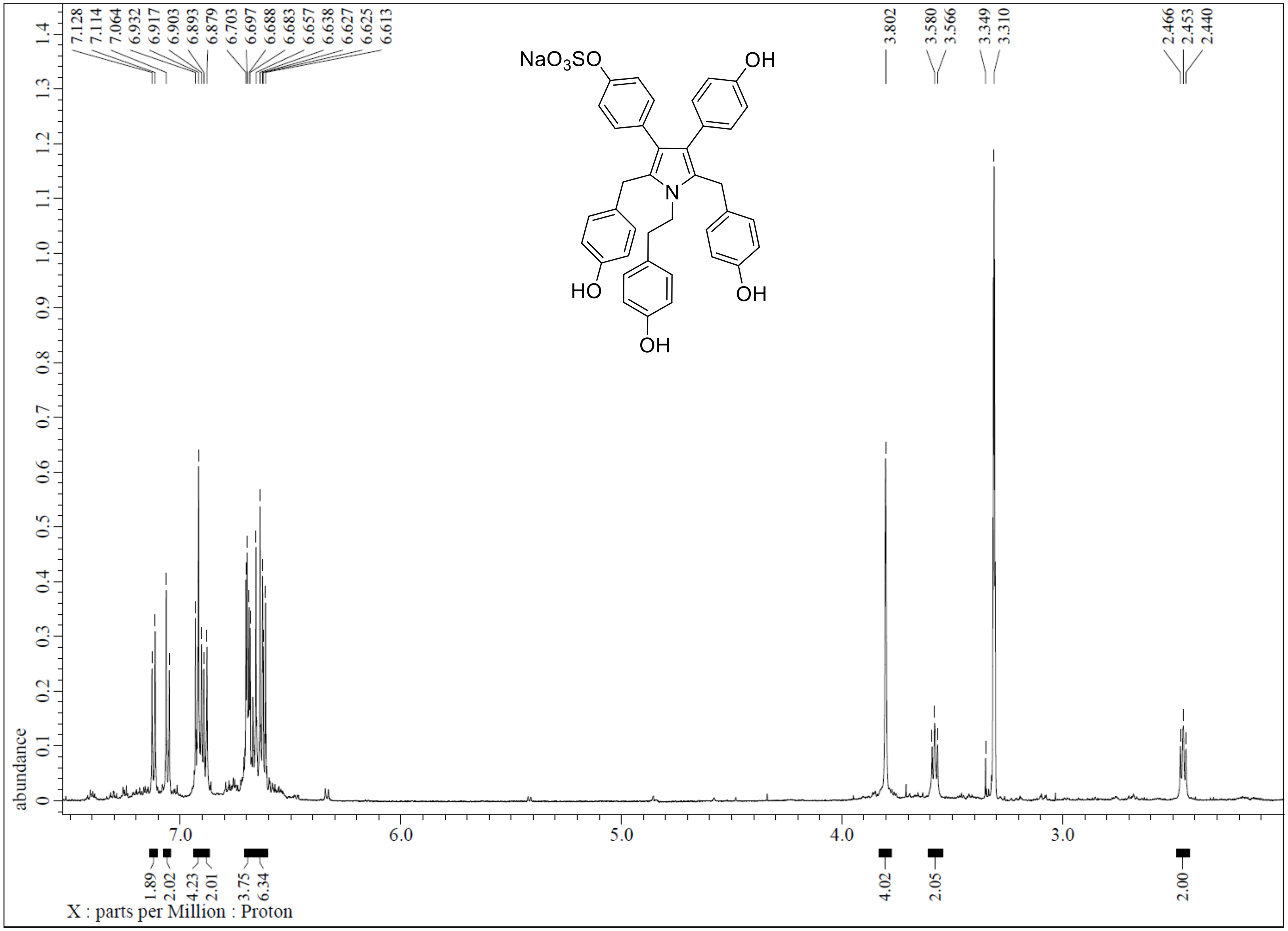

**Figure S4b.** $^{13}$C NMR (methanol-*d4*, 150 MHz) spectrum of denigrin K (**4**)

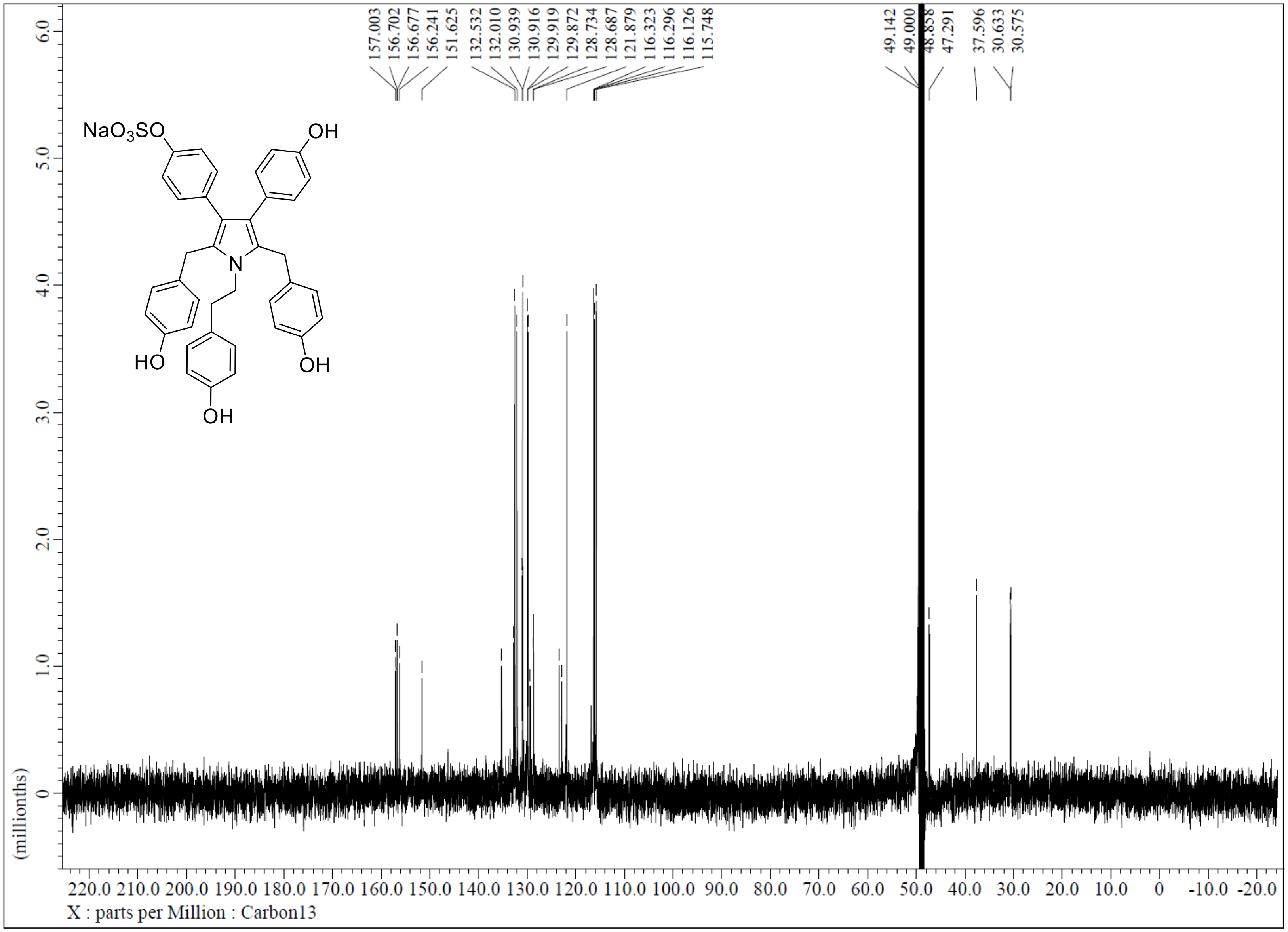

**Figure S4c.** $^{13}$C NMR (methanol-*d4*, 150 MHz) spectrum expansion 1 of denigrin K (**4**)

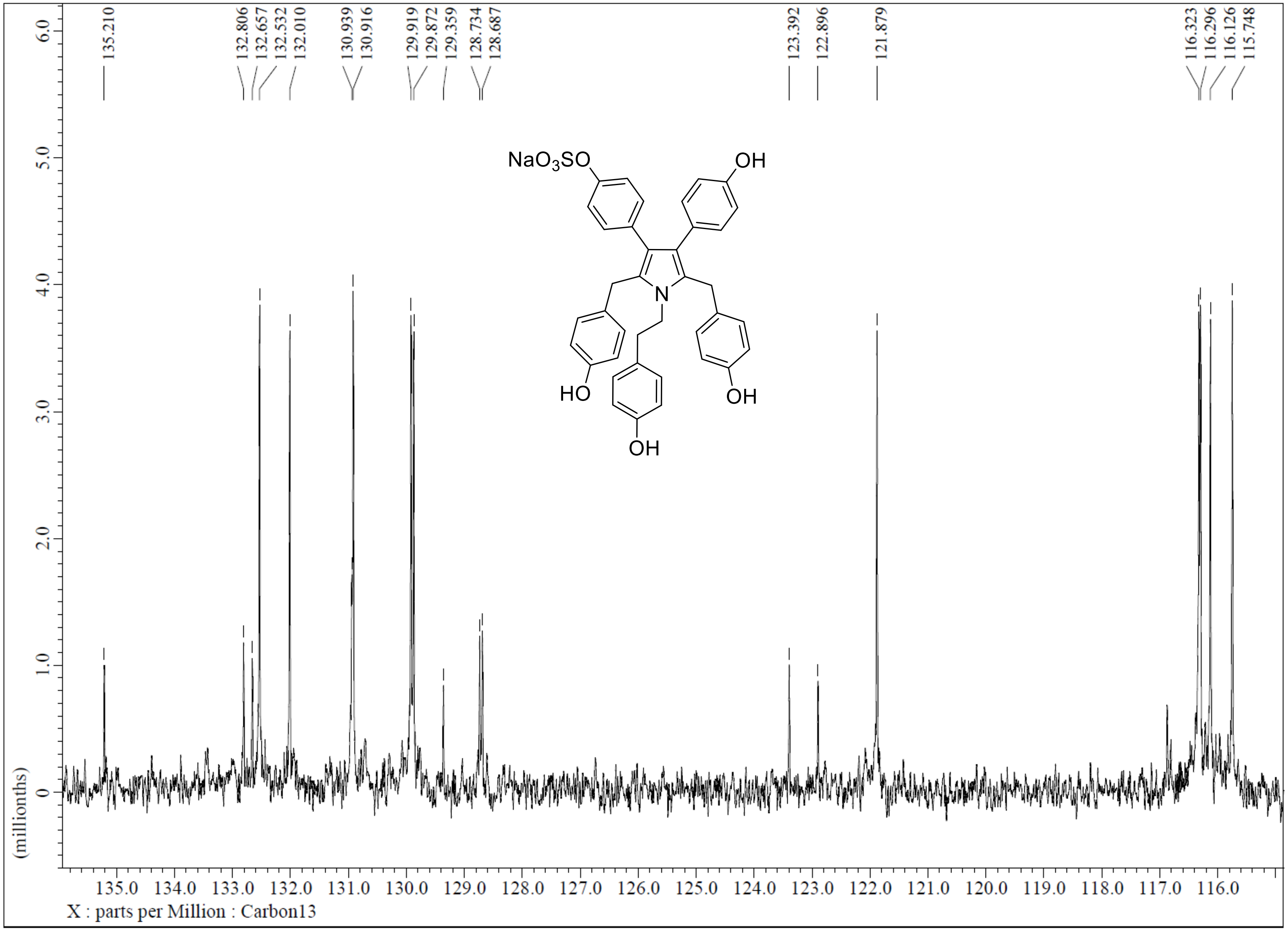

**Figure S4d.** $^{13}$C NMR (methanol-*d4*, 150 MHz) spectrum expansion 2 of denigrin K (**4**)

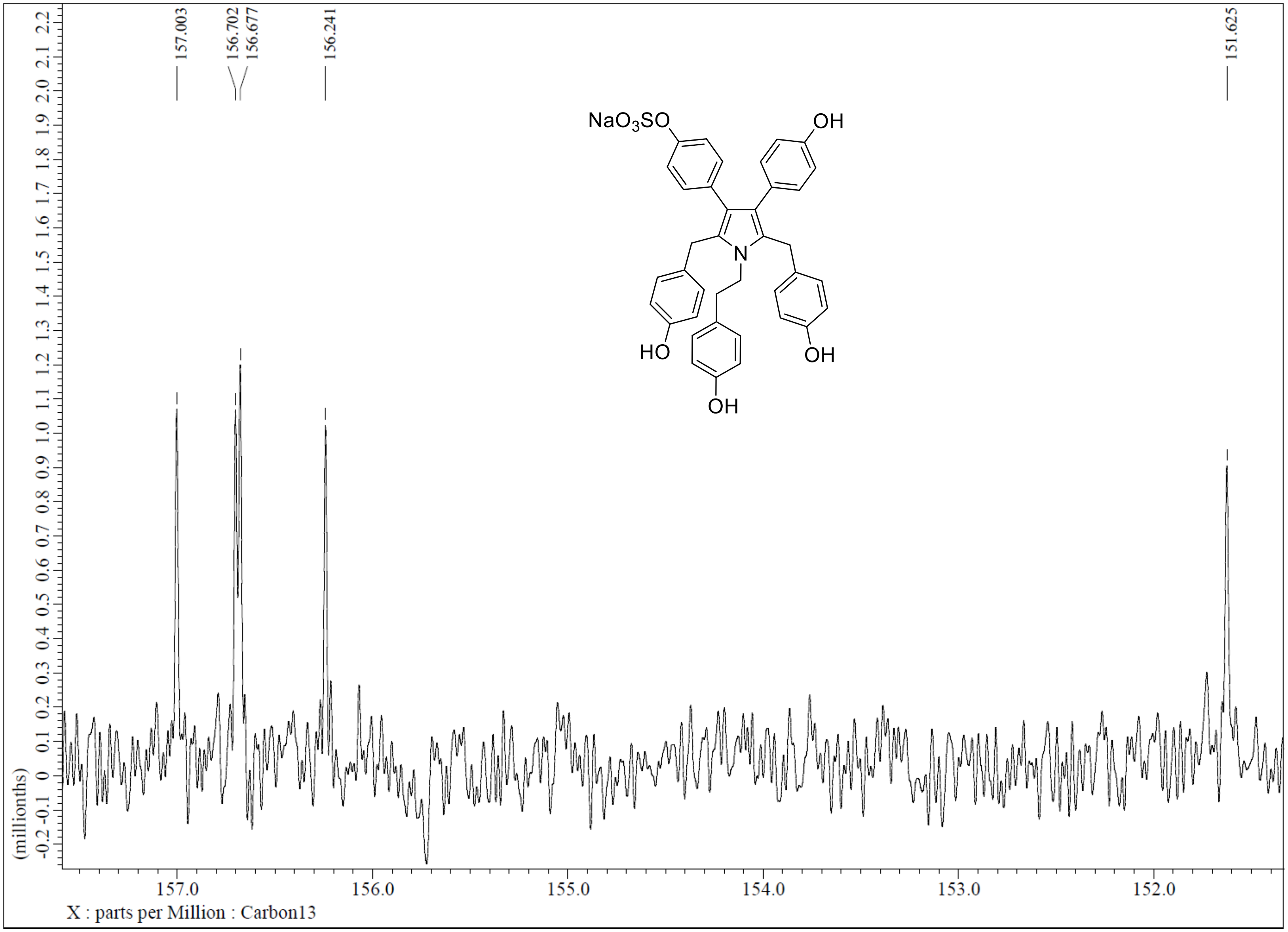

**Figure S4e.** COSY (methanol-*d4*) spectrum of denigrin K (**4**)

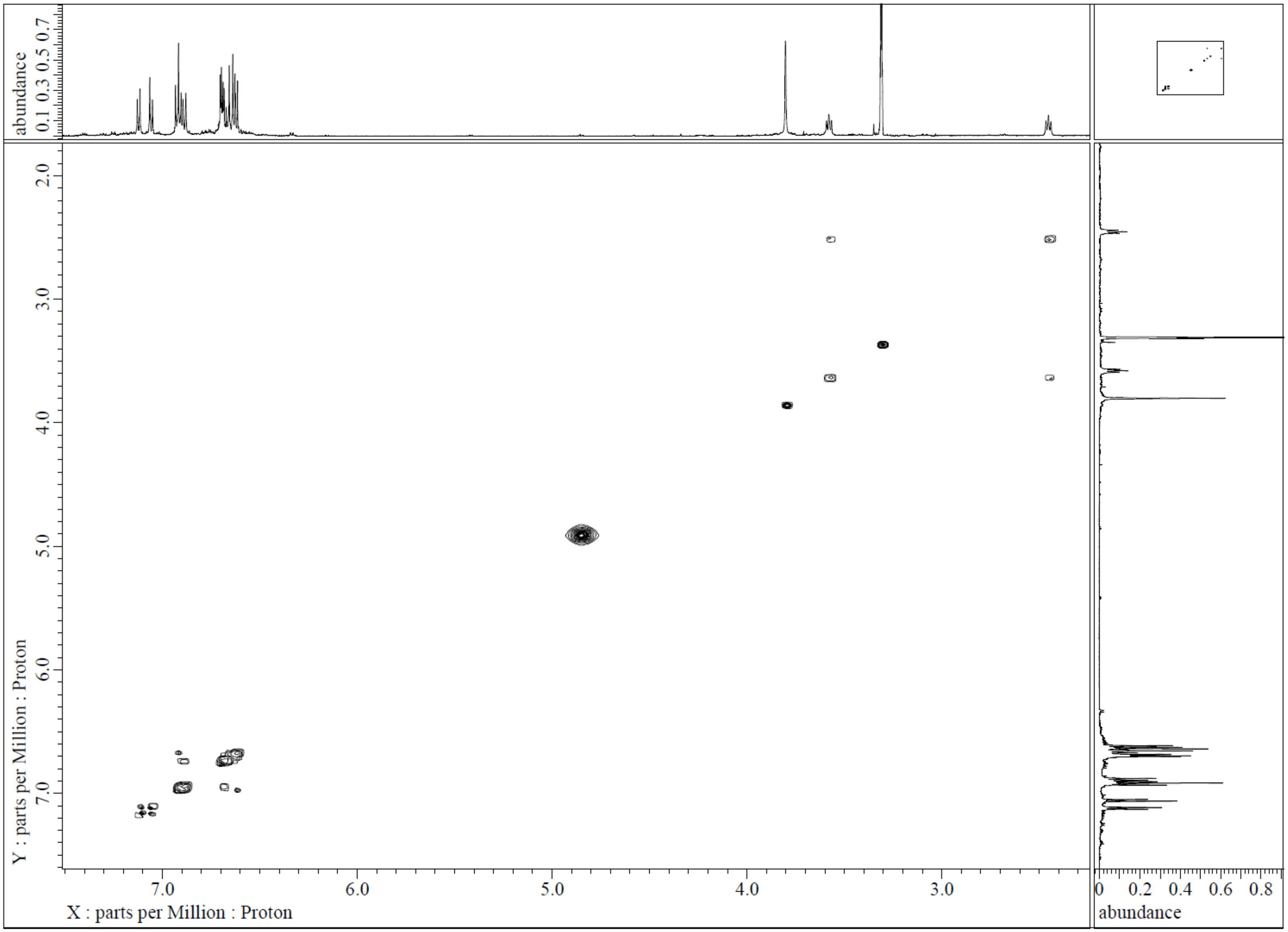

**Figure S4f.** gHSQC (methanol-*d4*) spectrum of denigrin K (**4**)

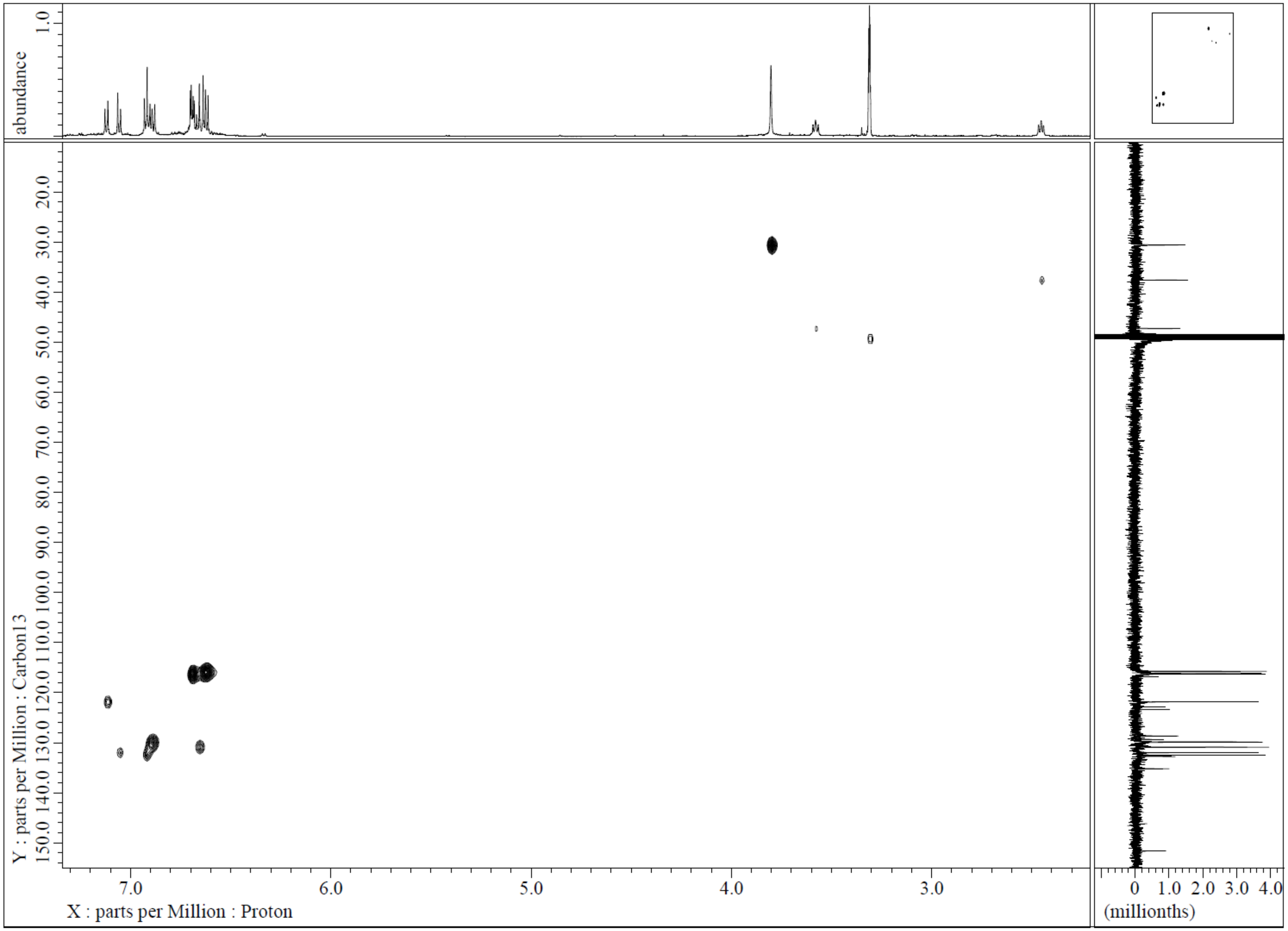

**Figure S4g.** gHMBC (methanol-*d4*) spectrum of denigrin K (**4**)

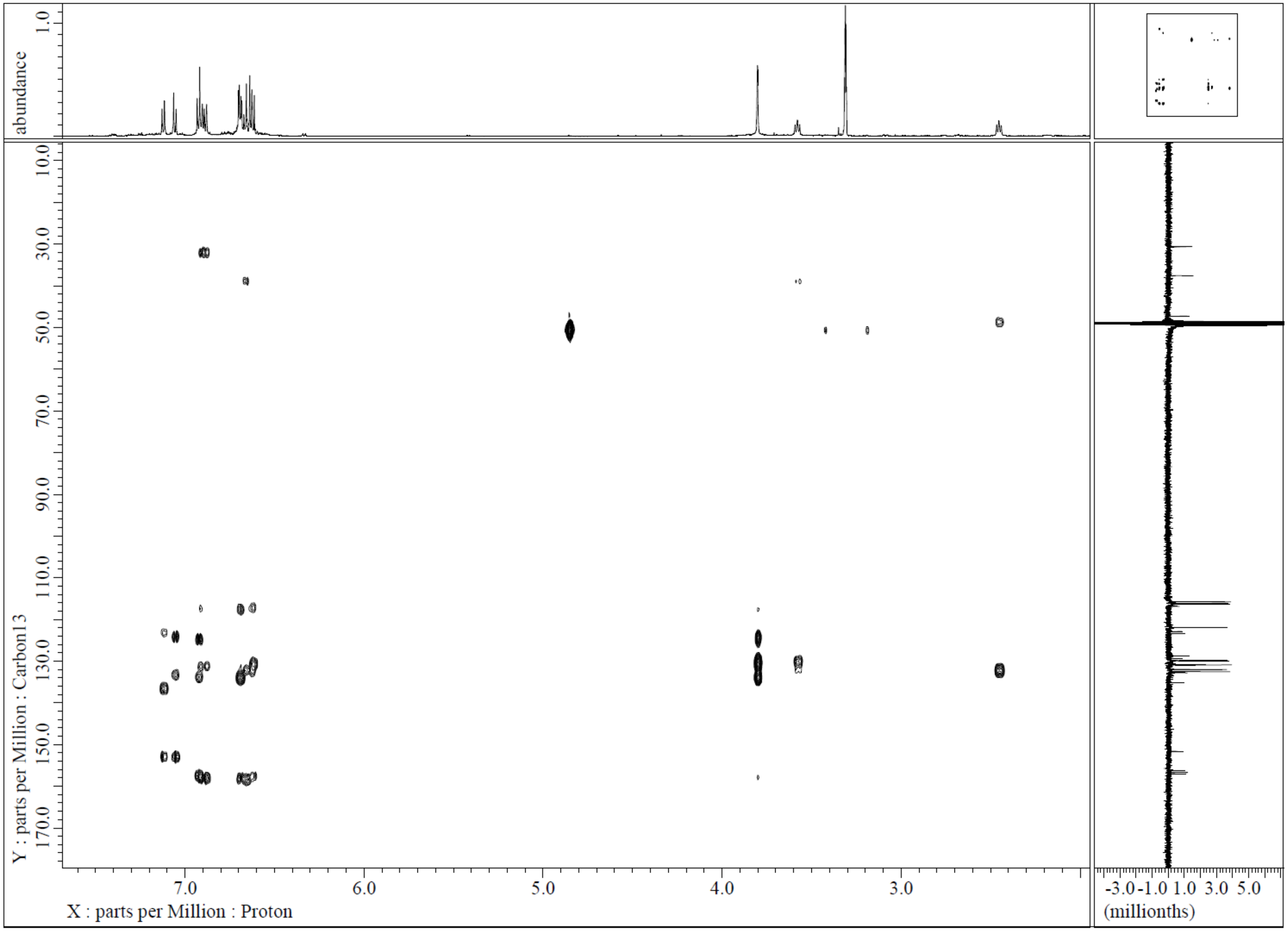

**Figure S4h.** gHMBC (methanol-*d4*) spectrum expansion of denigrin K (**4**)

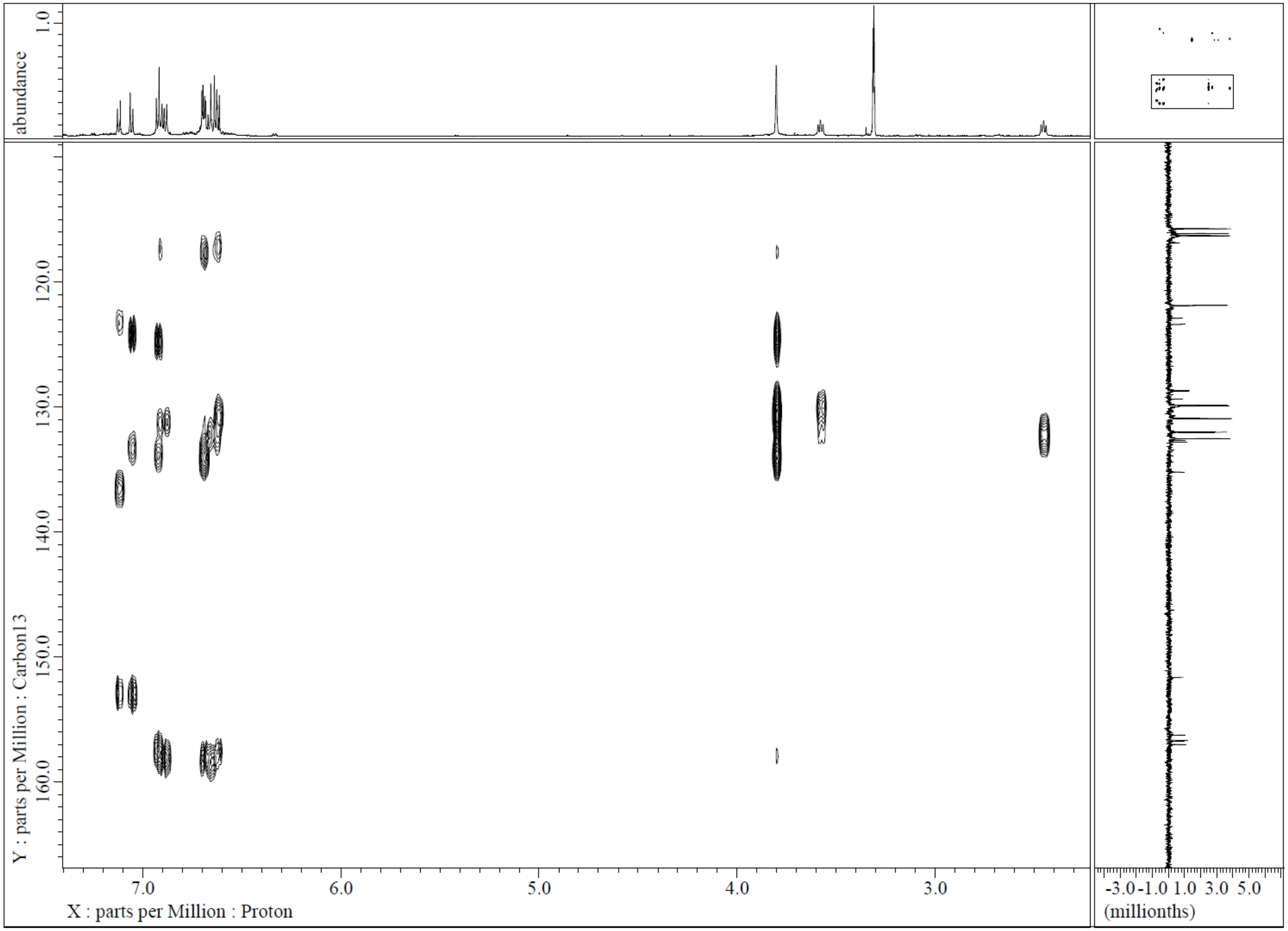

**Figure S4i**. HRESIMS spectrum of denigrin K (**4**) in negative ion mode

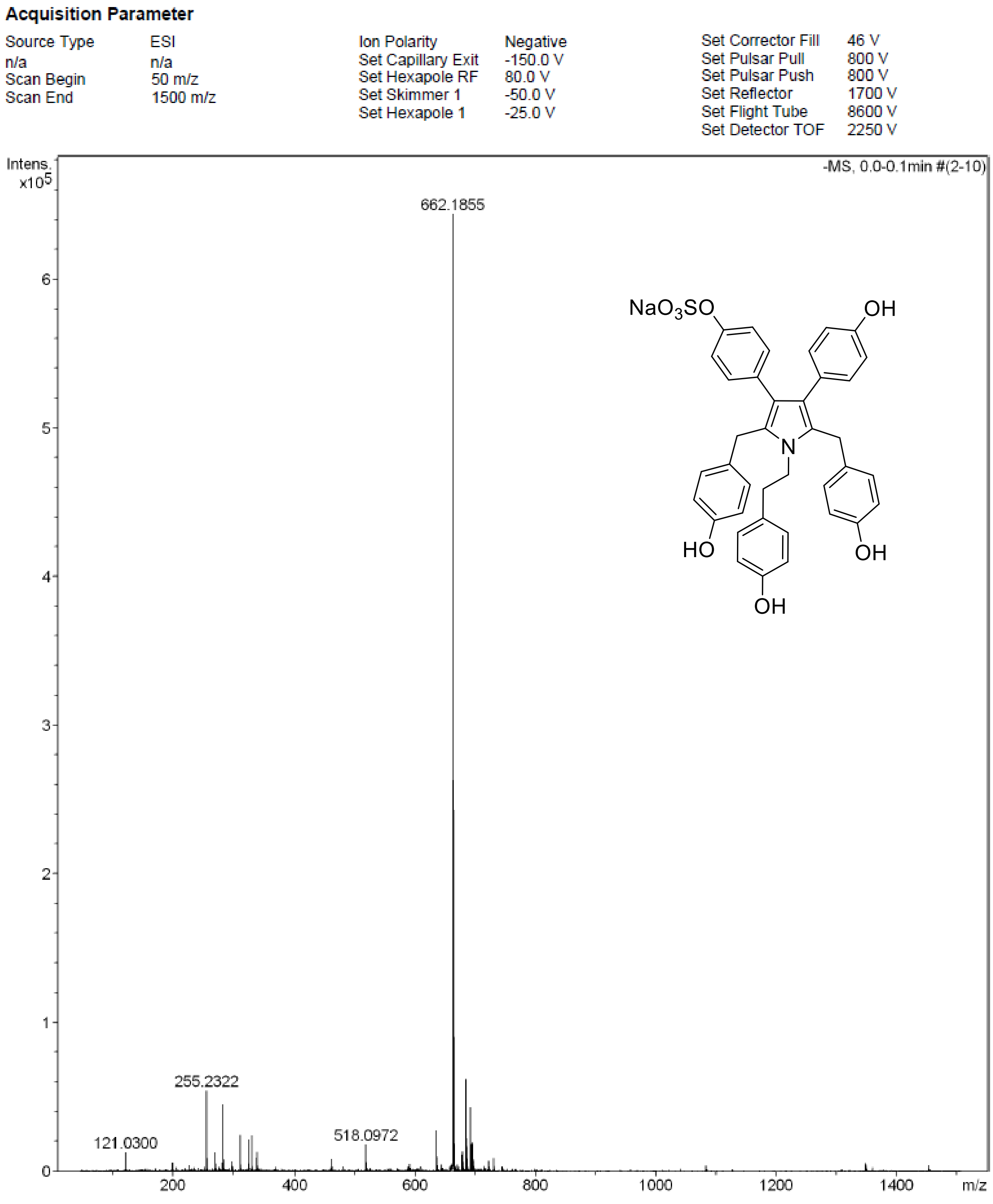

**Figure S5a.** $^1$H NMR (methanol-*d4*, 600 MHz) spectrum of denigrin L (**5**)

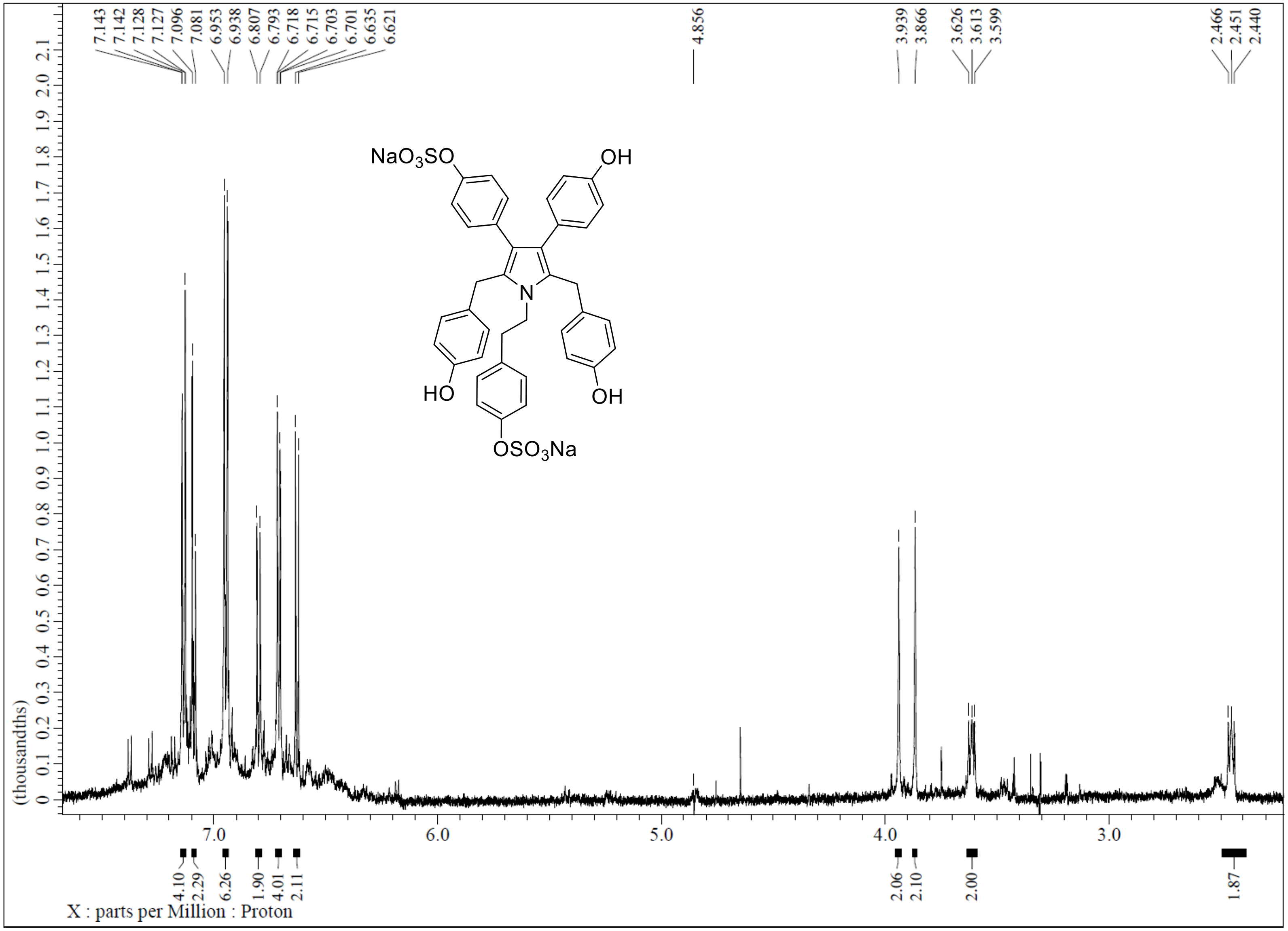

**Figure S5b.** $^{13}C$ NMR (methanol-*d4*, 150 MHz) spectrum of denigrin L (**5**)

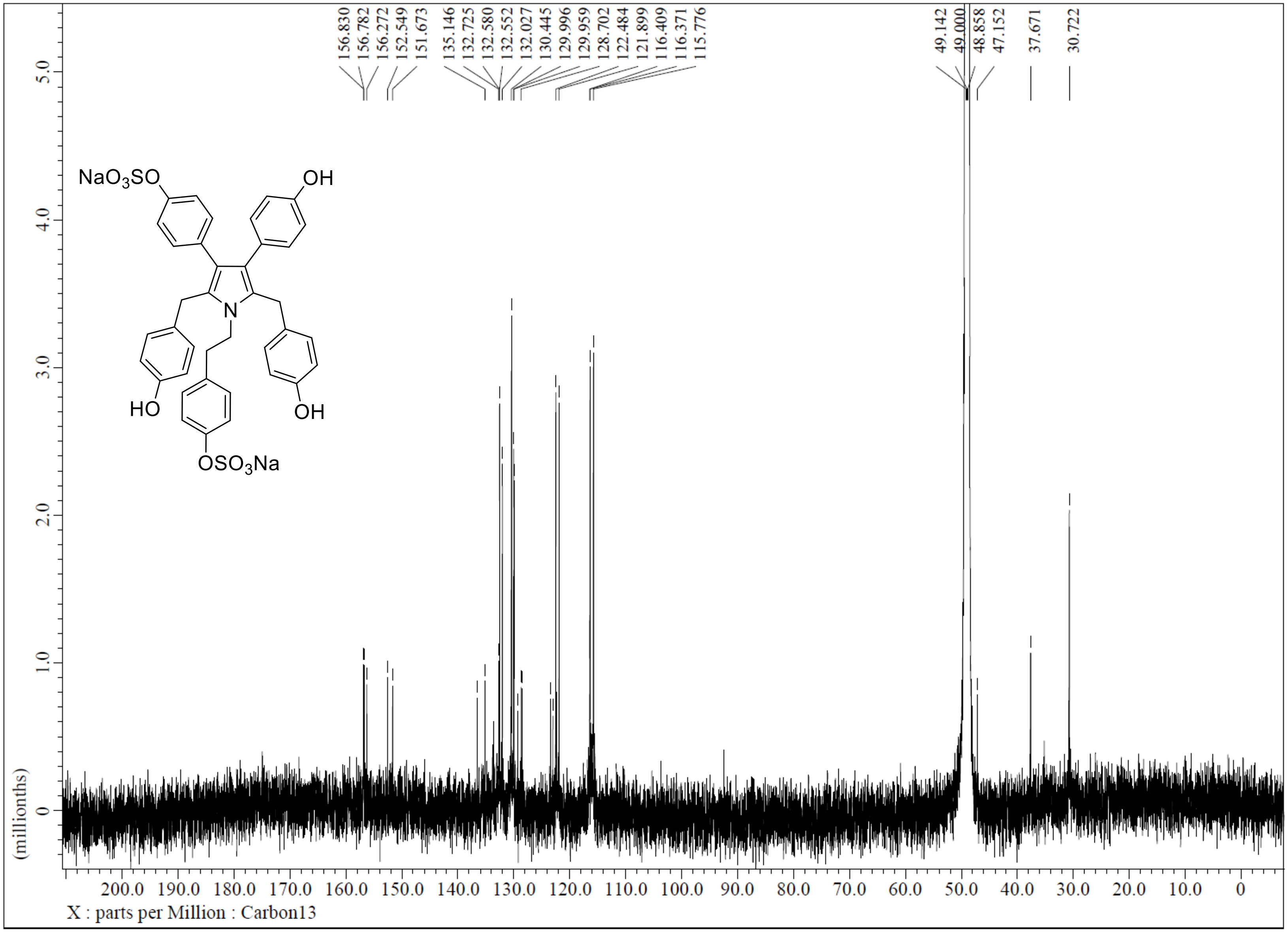

**Figure S5c.** $^{13}$C NMR (methanol-*d4*, 150 MHz) spectrum expansion of denigrin L (**5**)

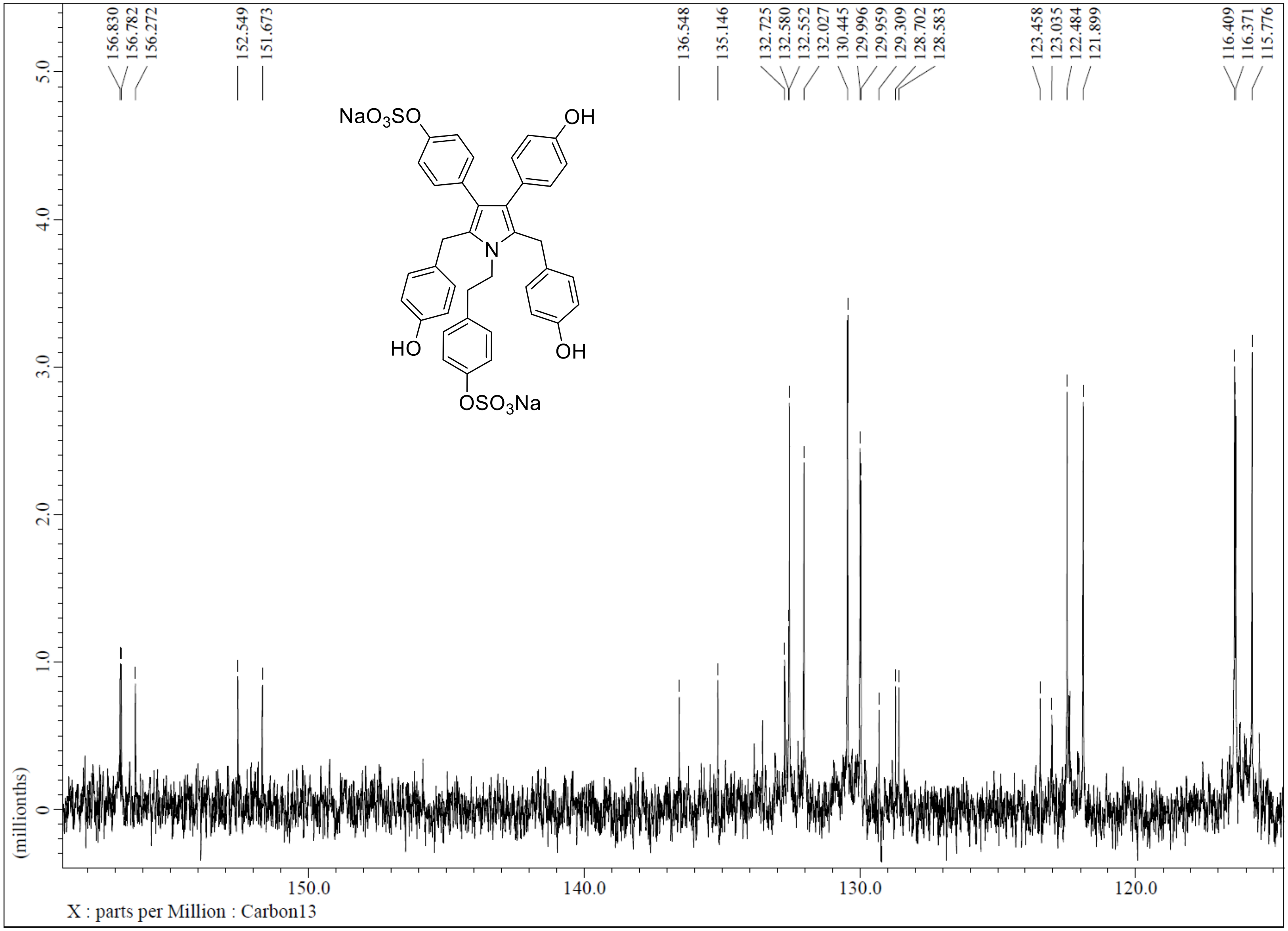

**Figure S5d.** COSY (methanol-*d4*) spectrum of denigrin L (**5**)

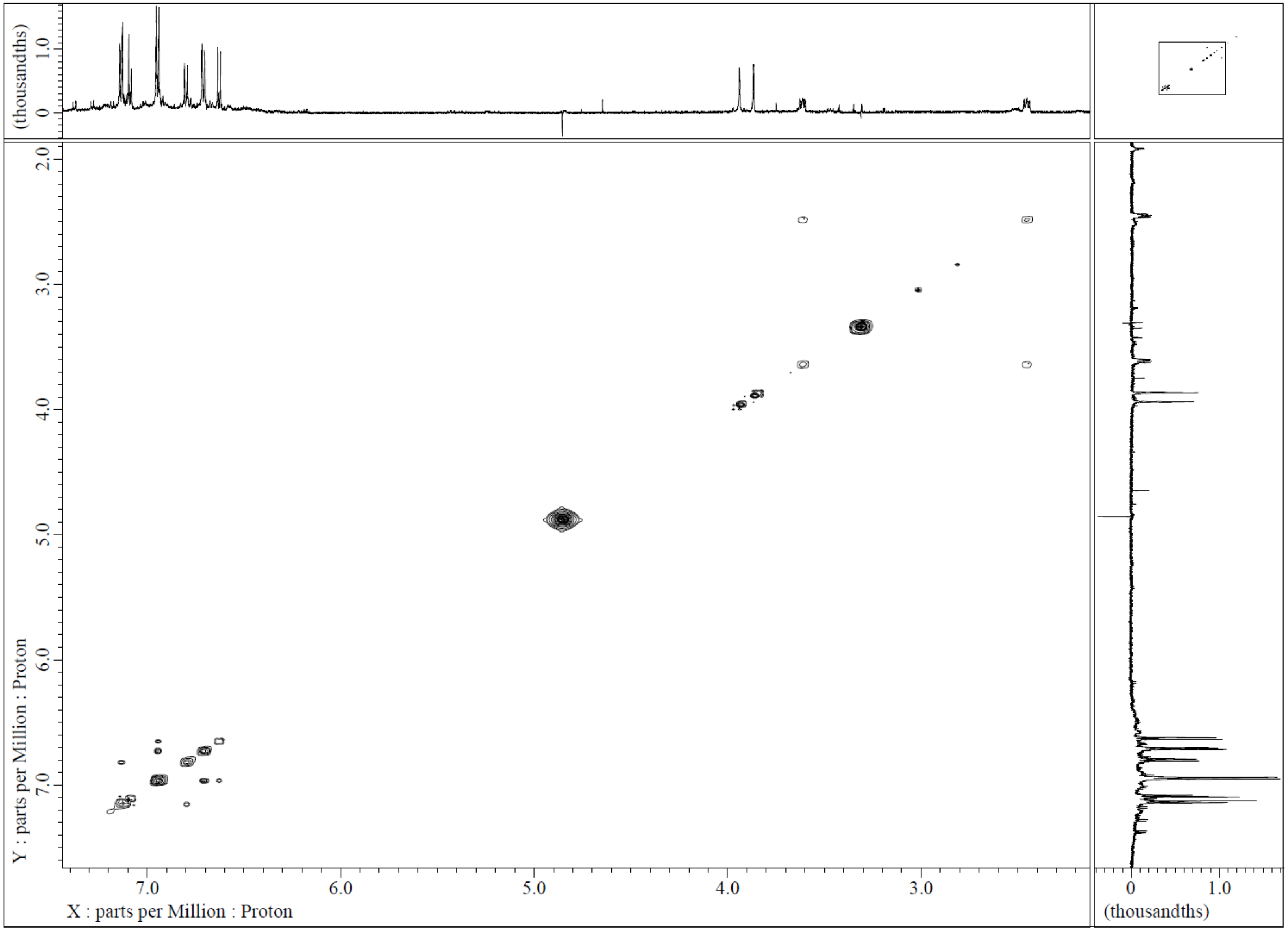

**Figure S5e.** gHSQC (methanol-*d4*) spectrum of denigrin L (**5**)

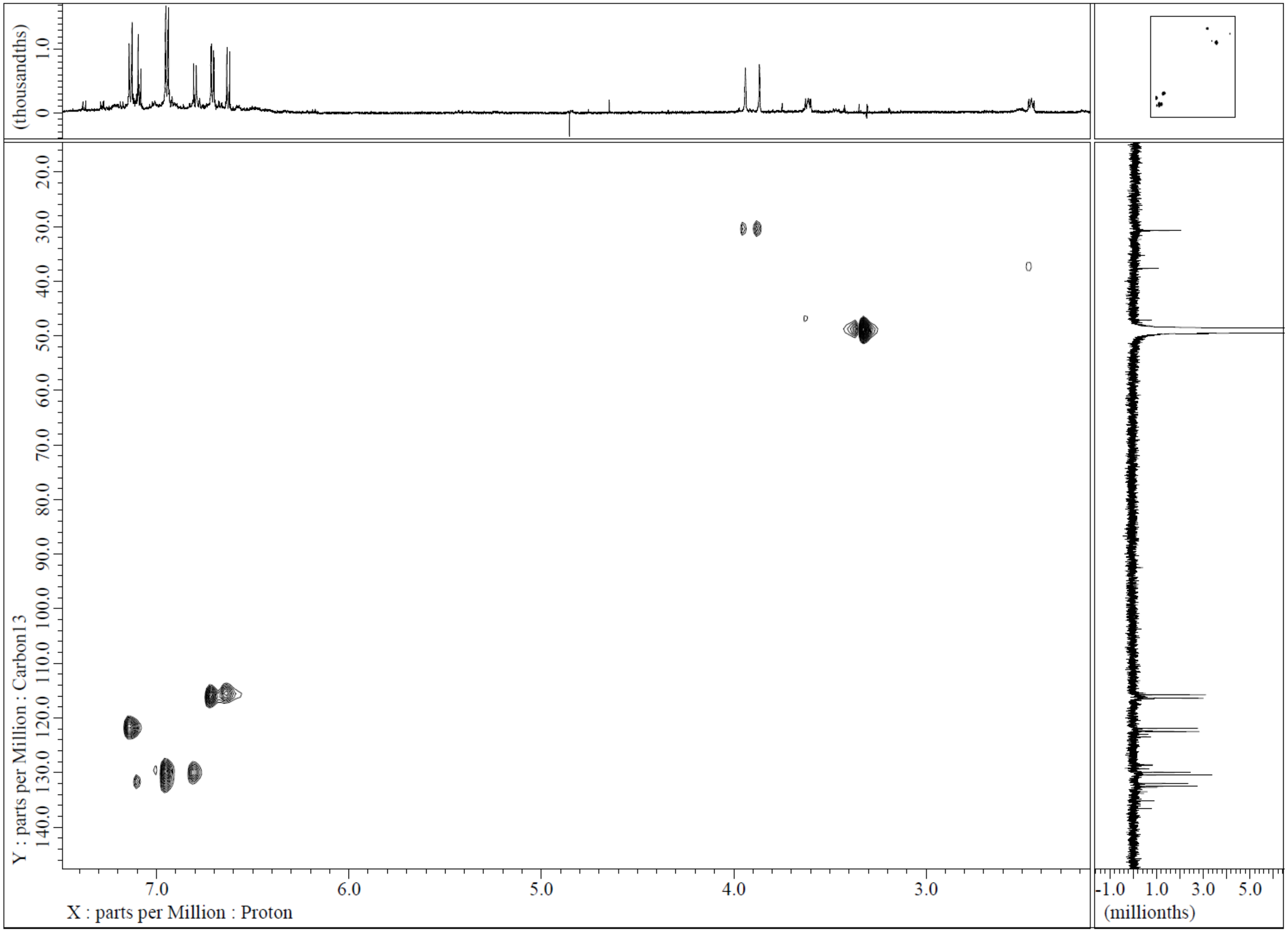

**Figure S5f.** gHMBC (methanol-*d4*) spectrum of denigrin L (**5**)

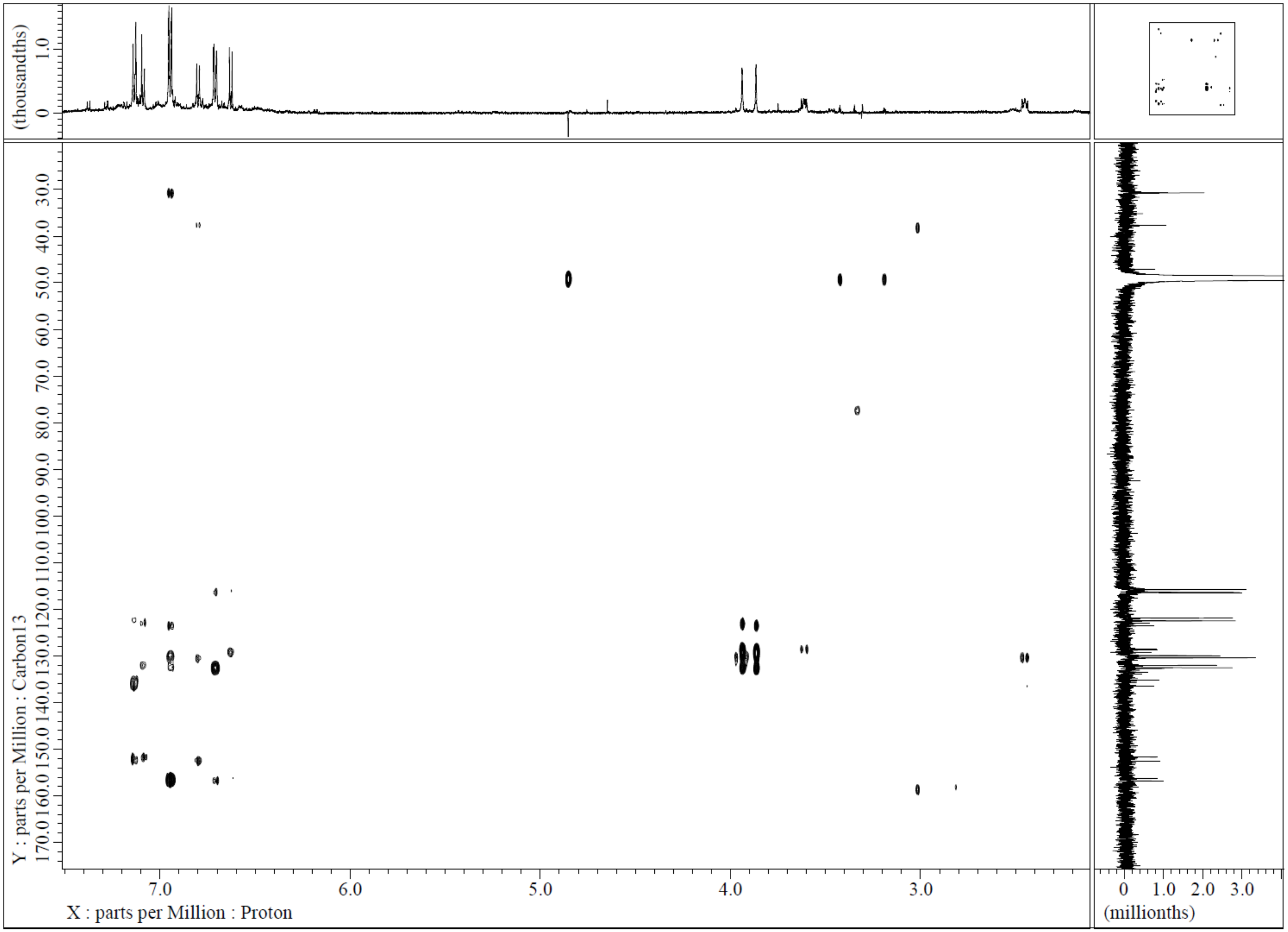

**Figure S5g.** gHMBC (methanol-*d4*) spectrum expansion of denigrin L (**5**)

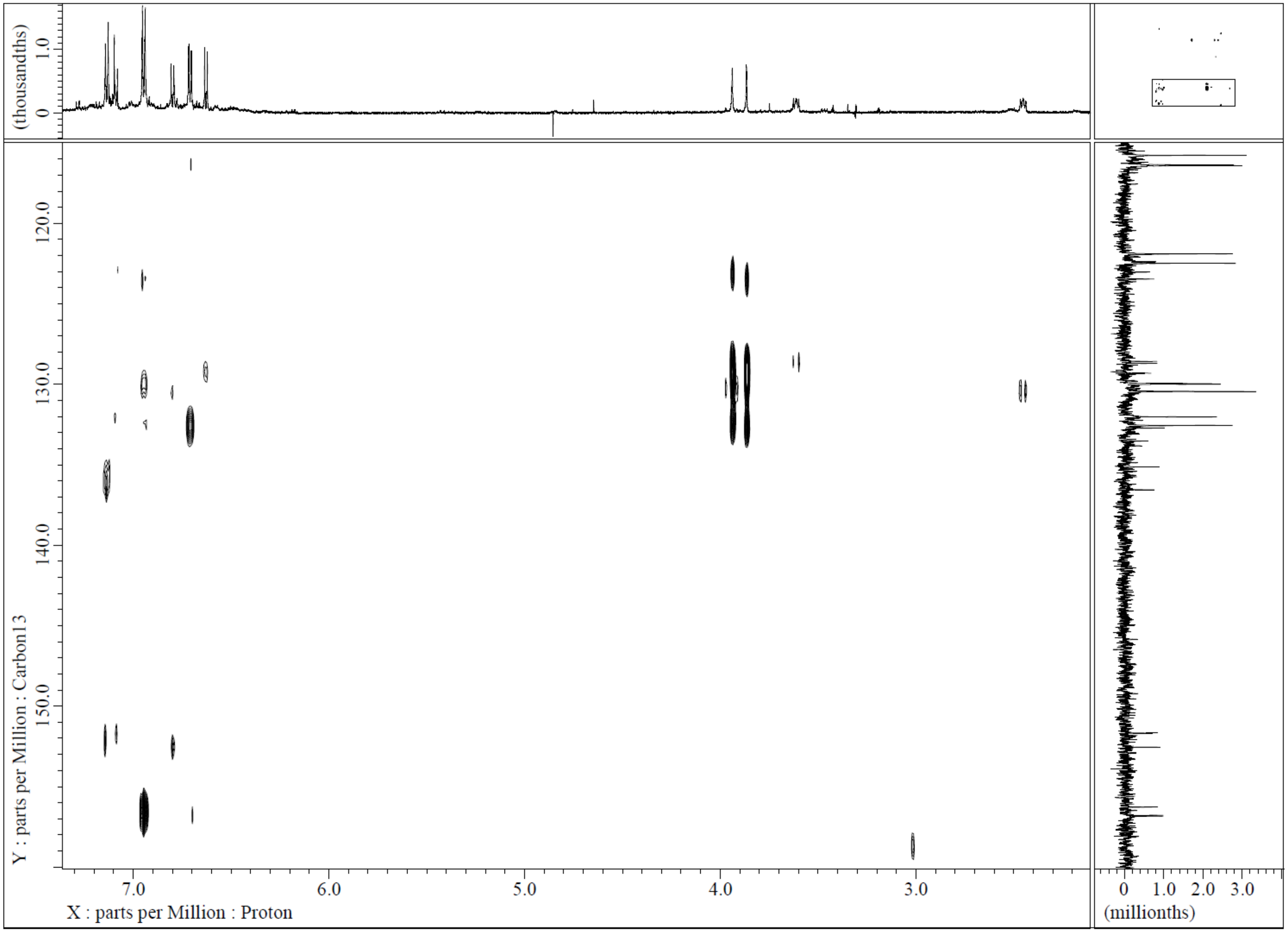

**Figure S5h**. HRESIMS spectrum of denigrin L (**5**) in negative ion mode

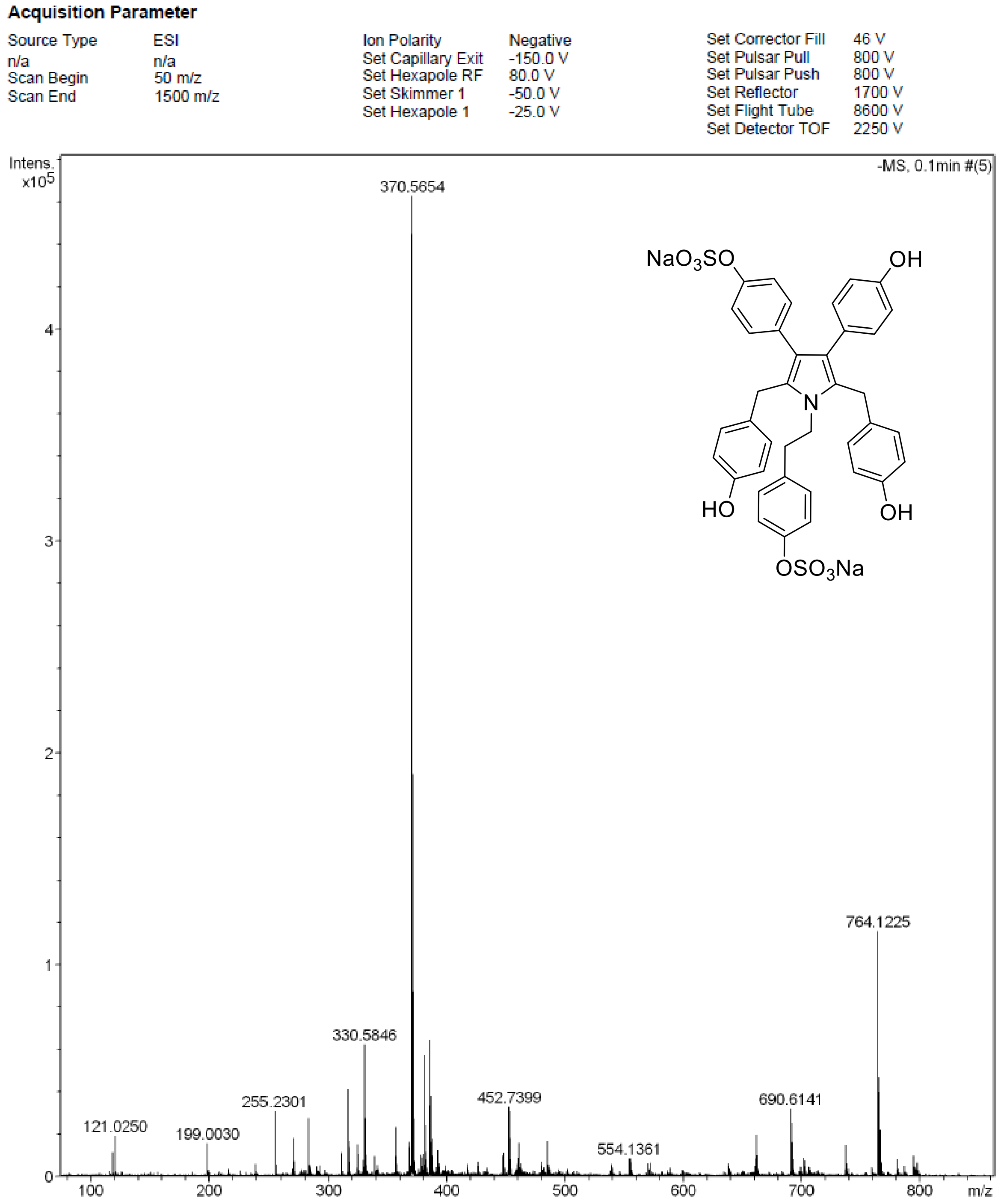

**Figure S6a.** $^{1}$H NMR (methanol-*d4*, 600 MHz) spectrum of denigrin E (**6**)

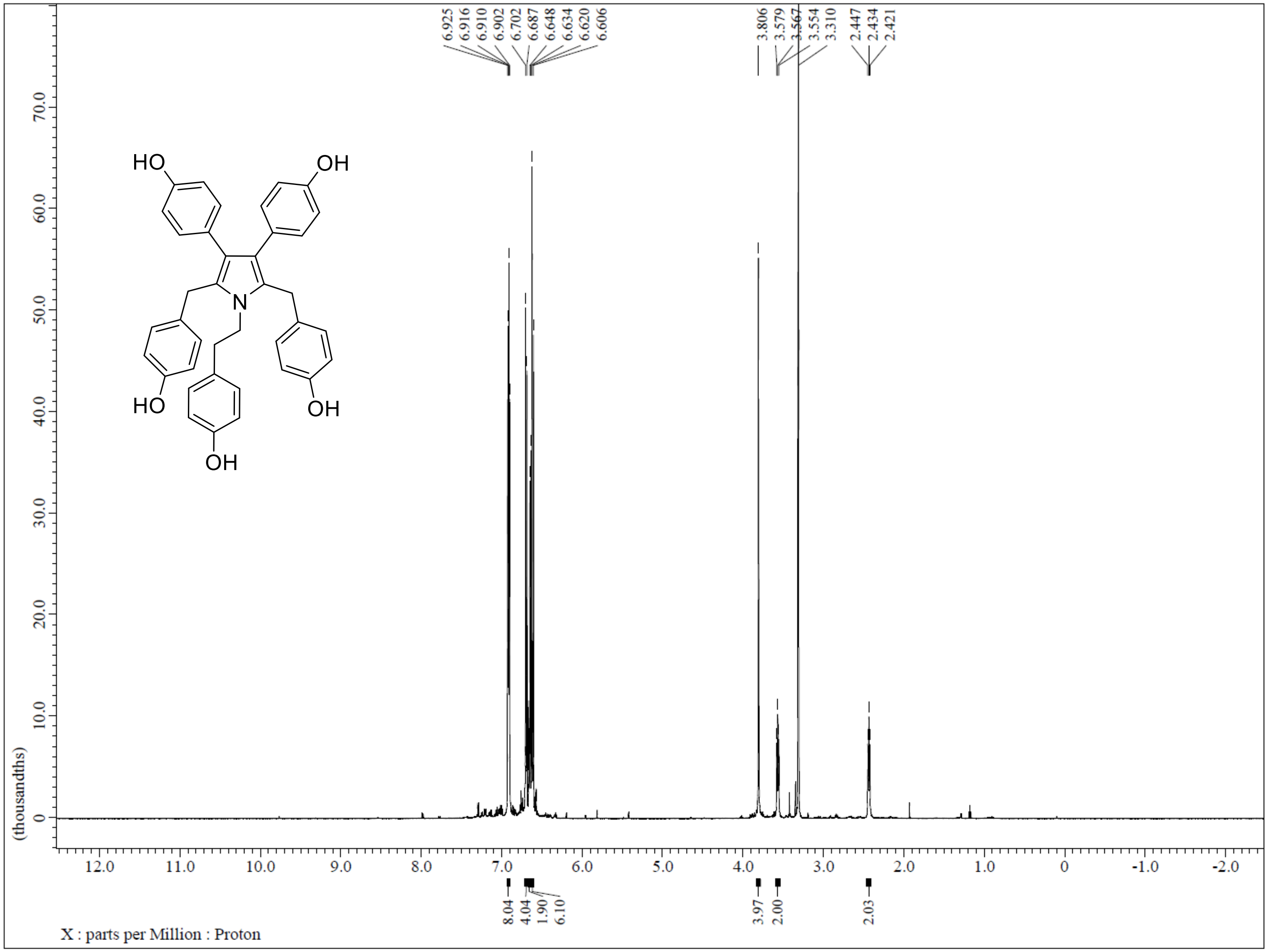

**Figure S6b.** $^{13}C$ NMR (methanol-*d4*, 150 MHz) spectrum of denigrin E (**6**)

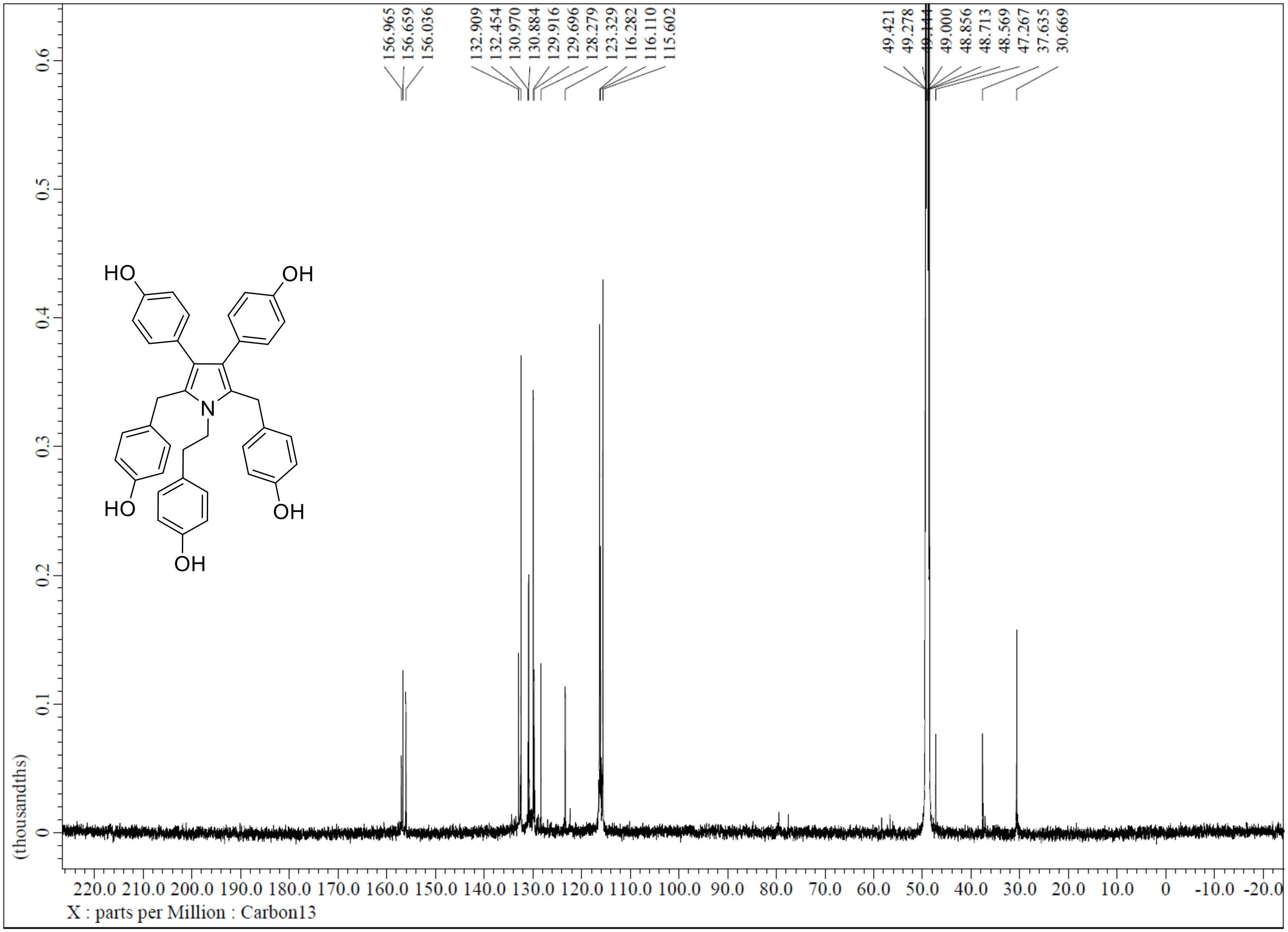

**Figure S6c.** $^{13}$C NMR (methanol-*d4*, 150 MHz) spectrum expansion of denigrin E (**6**)

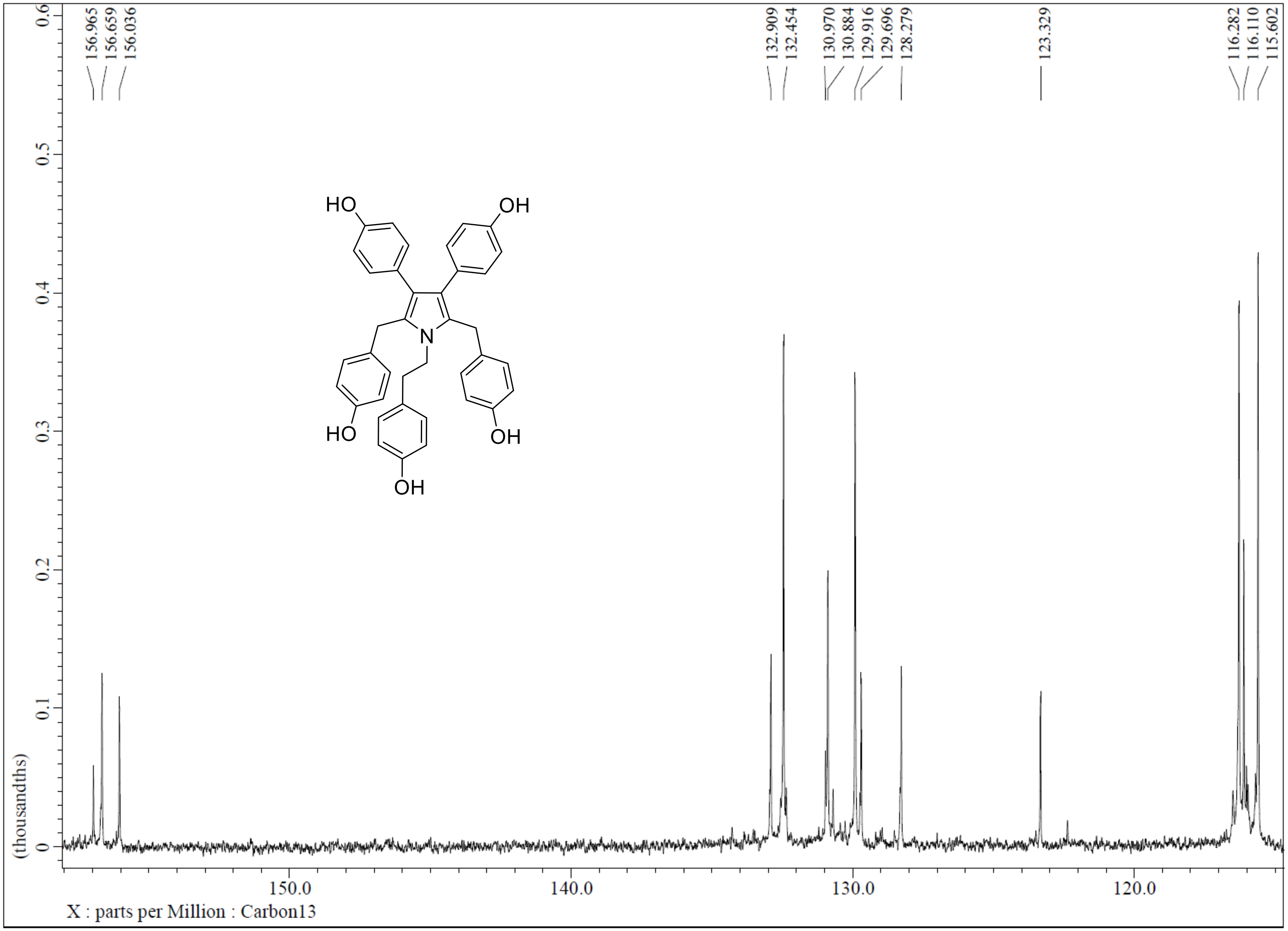

**Figure S7.** $^1$H NMR (methanol-*d4*, 600 MHz) spectrum of dictyodendrin C

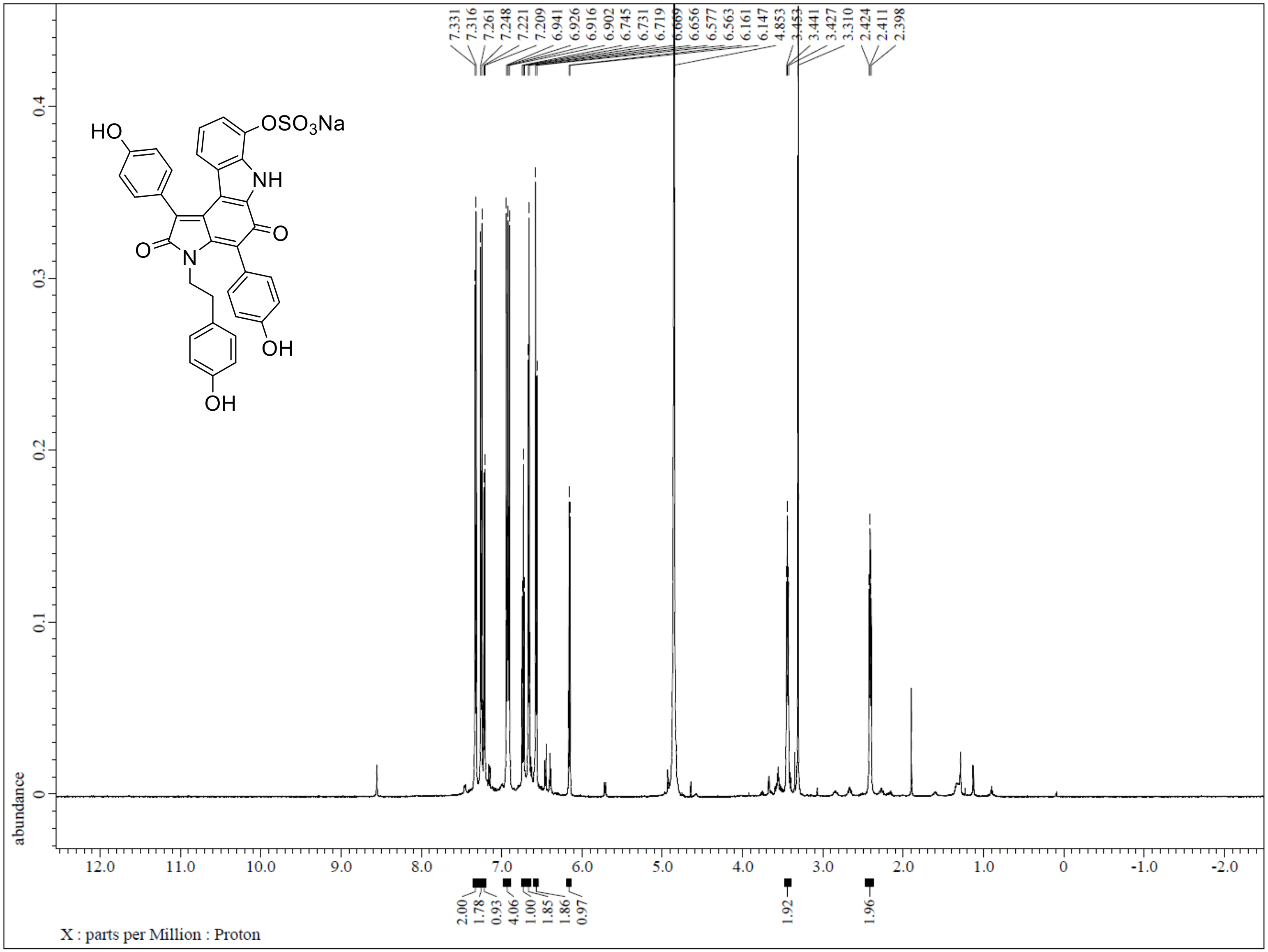

**Figure S8.** $^{1}$H NMR (methanol-*d4*, 600 MHz) spectrum of dictyodendrin D

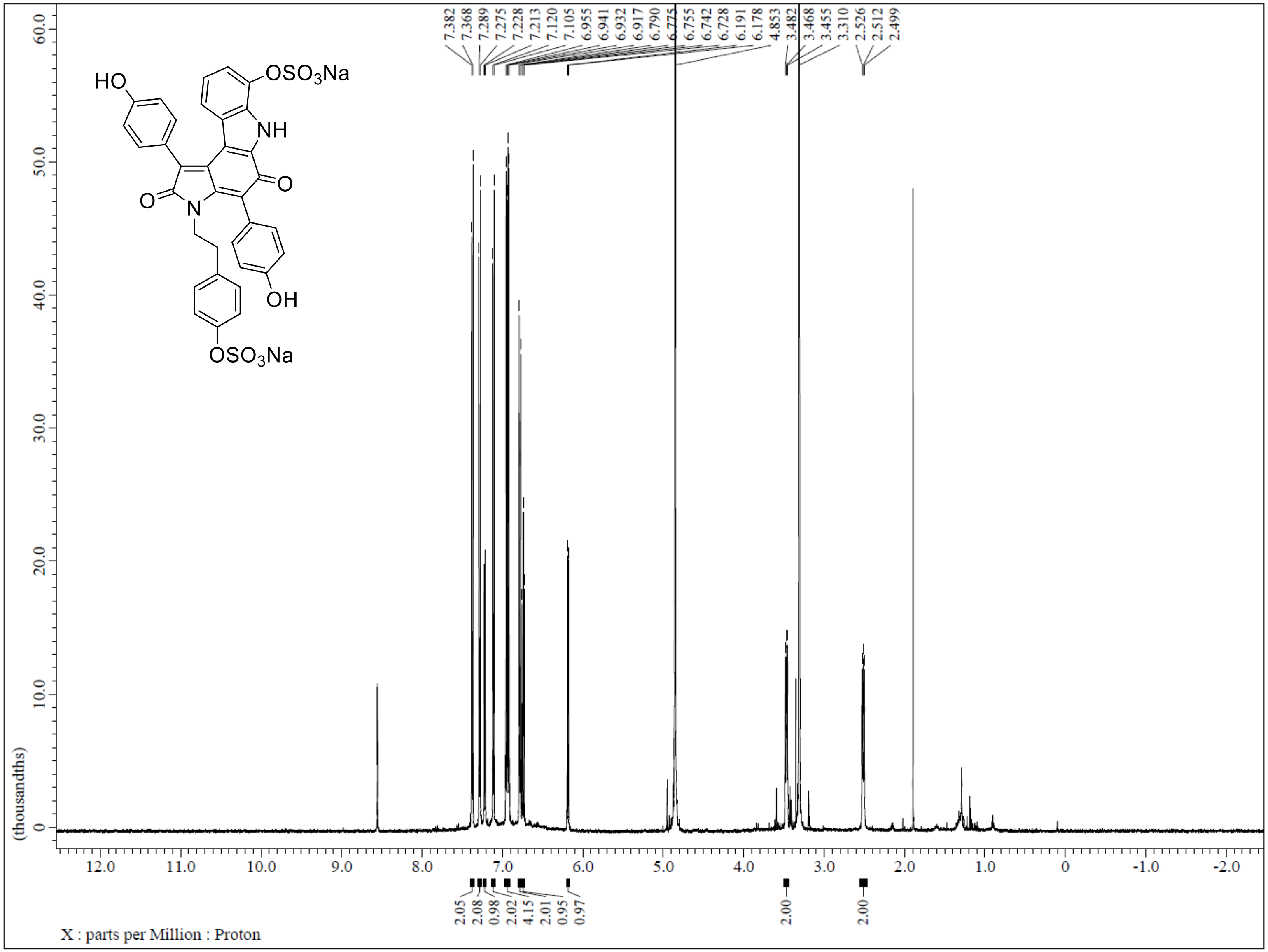

**Figure S9.** $^{1}H$ NMR (methanol-*d4*, 600 MHz) spectrum of dictyodendrin F

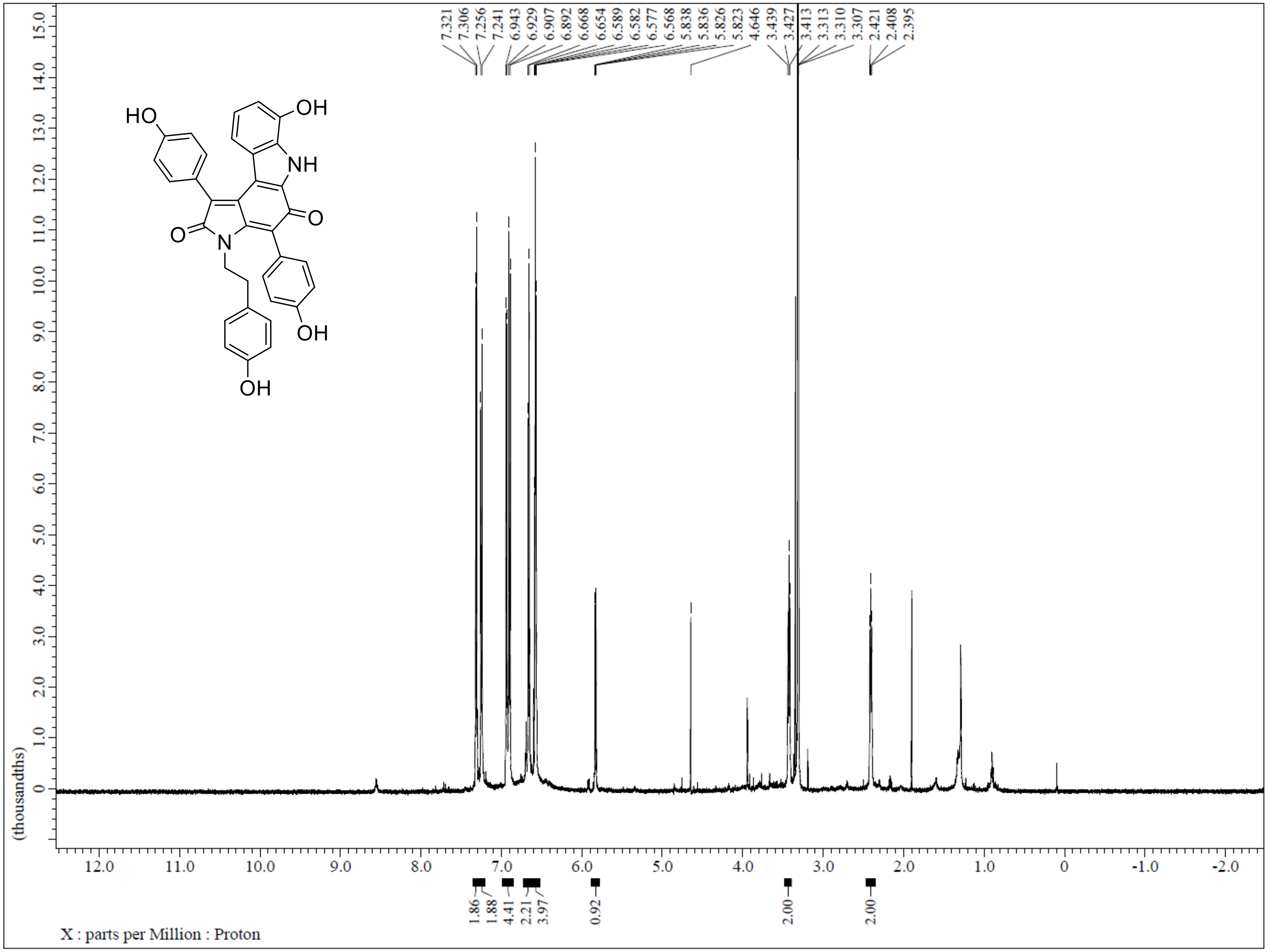

**Figure S10.** $^{1}$H NMR (methanol-*d4*, 600 MHz) spectrum of dactypyrrole A

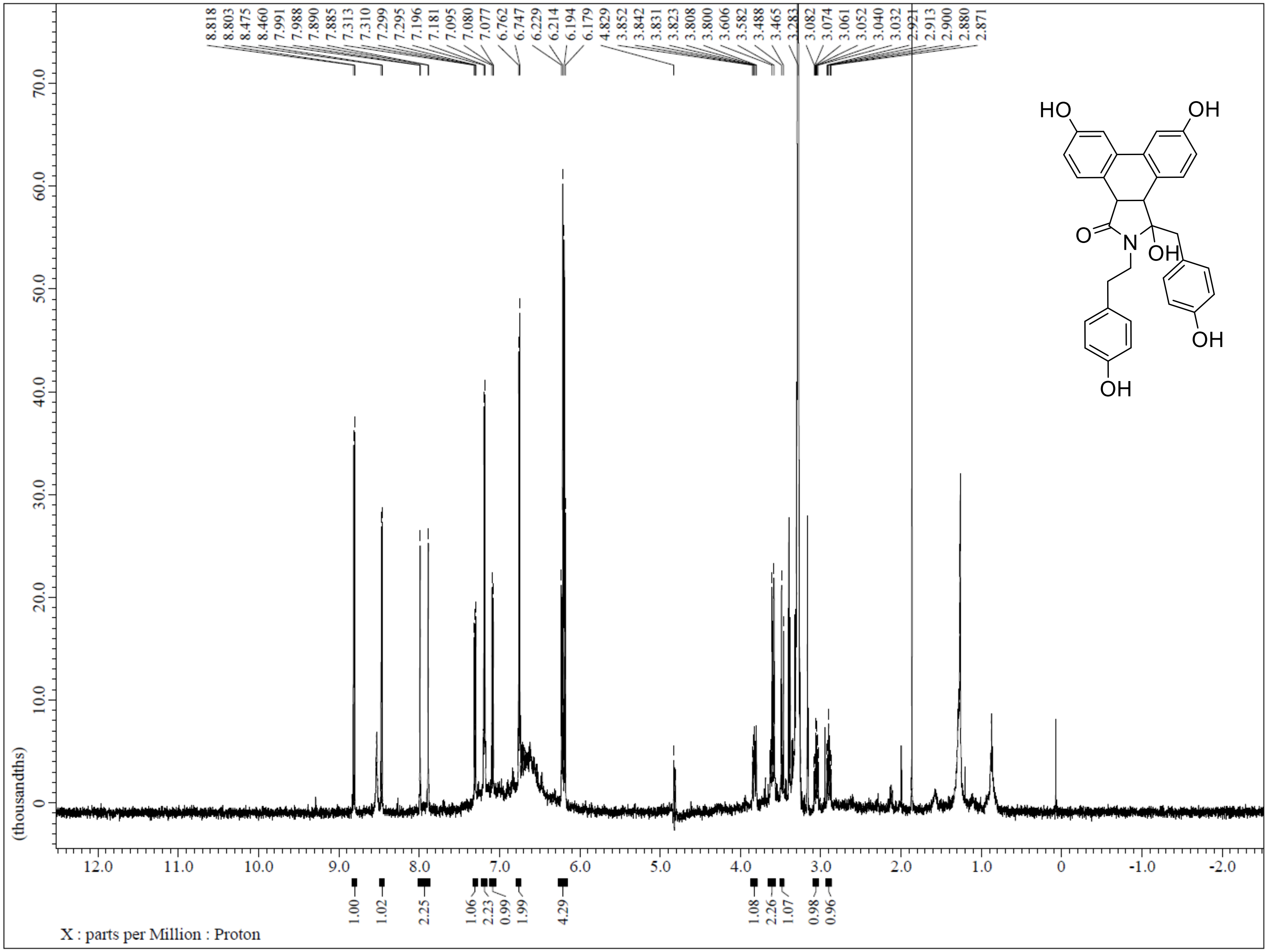

**Figure S11**. $^1$H NMR (methanol-*d4*, 600 MHz) spectrum of lamellarin O1

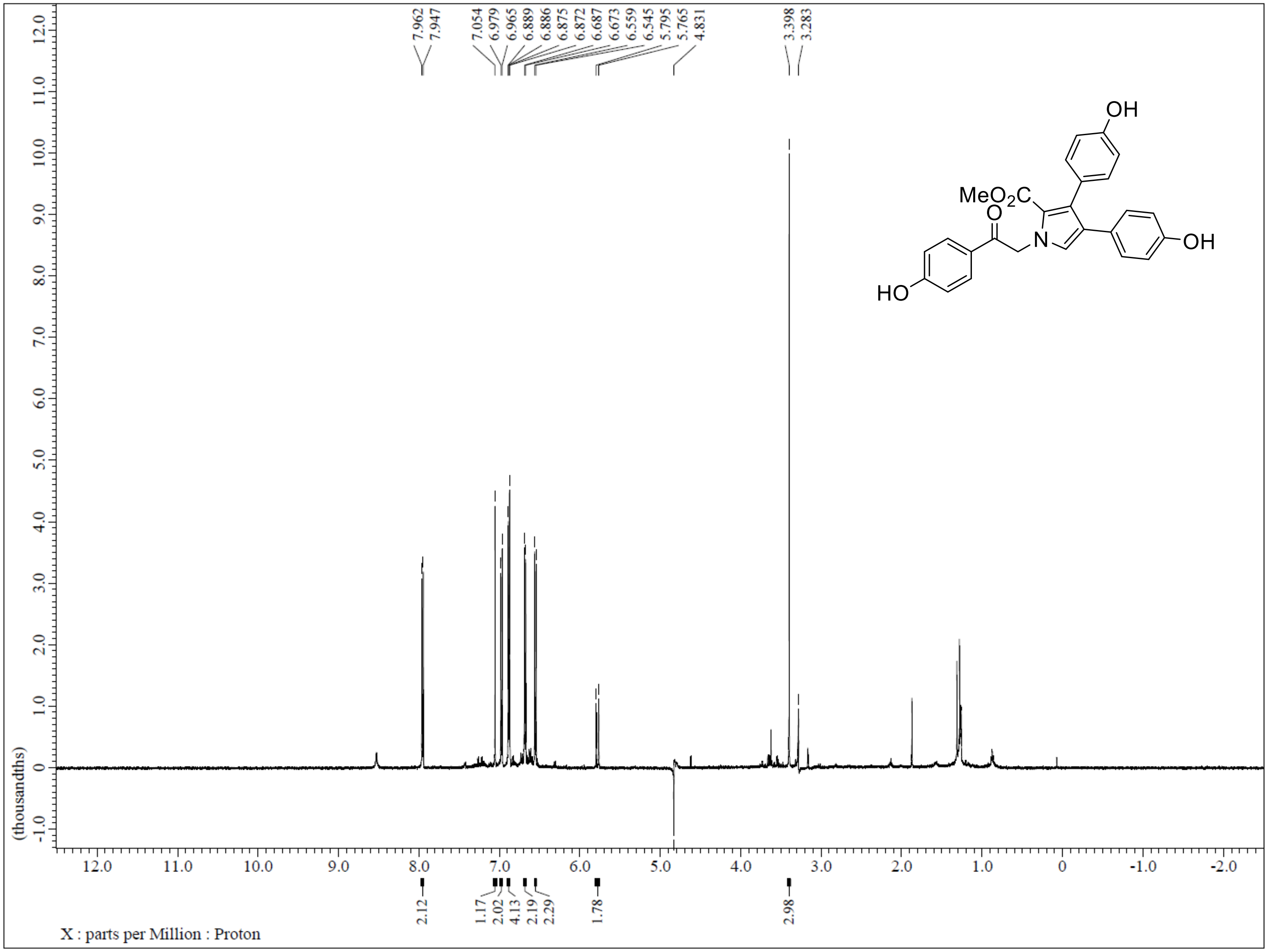

**Table S6**. Cytotoxicity assay - $IC_{50}$ values for compounds **1**-**6**, dictyodendrin C, D, and F, dactylpyrrole A and lamellarin O1

| **Compound** | **$IC_{50}$ (µM)** |
|---|---|
| 1 | > 100 |
| 2 | 78 |
| 3 | > 100 |
| 4 | > 100 |
| 5 | > 100 |
| 6 | > 100 |
| Dictyodendrin C | > 100 |
| Dictyodendrin D | 60 |
| Dictyodendrin F | 48 |
| Dactypyrrole A | > 100 |
| Lamellarin O1 | > 100 |
